# Global burden and forecast of infectious diseases attributable to drug use: evidence from GBD 2021

**DOI:** 10.3389/fpubh.2025.1706764

**Published:** 2025-12-15

**Authors:** Xueqing Gong, Yuanqian Yao, Lu Wang, Kairui Meng, Hui Li

**Affiliations:** 1 Hospital of Chengdu University of Traditional Chinese Medicine, Chengdu, China; 2 Clinical Medical College, Chengdu University of Traditional Chinese Medicine, Chengdu, China

**Keywords:** age-period-cohort analysis, drug use, forecasting, global burden of disease, health inequalities, infectious diseases

## Abstract

**Background:**

Drug use is a significant risk factor for infectious diseases, yet a comprehensive global assessment of this burden—encompassing trends, inequalities, and forecasts—is lacking, hindering progress toward World Health Organization (WHO)’s 2030 elimination targets. This study provides the first such analysis, leveraging the Global Burden of Disease (GBD) 2021 study to quantify the specific burden of infectious diseases attributable to drug use.

**Methods:**

Using data from 204 countries and territories in the GBD 2021 study, we analyzed deaths and disability-adjusted life years (DALYs) from HIV/AIDS, sexually transmitted infections (STIs), and other infectious diseases (primarily acute hepatitis B and C) attributable to drug use, following the GBD risk attribution framework. Methods included trend analysis, health inequality assessment, driver decomposition, age-period-cohort modeling, and Auto-Regressive Integrated Moving Average (ARIMA) forecasting to 2036.

**Results:**

From 1990 to 2021, the global age-standardized mortality rate (ASMR) for HIV/AIDS and STIs attributable to drug use increased by 135%, peaking around 2005 before declining, while the ASMR for other infectious diseases decreased by 57%. Males aged 30–54 carried the highest burden. Health inequality analysis revealed a shift of HIV/AIDS and STIs toward high-Socio-demographic Index (SDI) countries. Epidemiological changes were the primary driver of burden trends. By 2036, the ASMR for HIV/AIDS and STIs is projected to decline by 82.9%.

**Conclusion:**

Drug use is a critical, evolving driver of infectious disease burden. Stratified interventions and coordinated supply–demand-side governance are essential to mitigate this burden and achieve global health goals.

## 1 Introduction

Infectious diseases remain a formidable challenge to global public health (1, 2). Their transmission is strongly influenced by behavioral risk factors, among which drug use is particularly concerning (3). The use of illicit drugs, such as opioids and amphetamines, not only causes substance use disorders but also creates a syndemic with infectious diseases (4–6). This convergence worsens health outcomes, burdens healthcare systems, and reinforces cycles of poverty and social exclusion. Drug use facilitates disease spread through several pathways, primarily by encouraging high-risk sexual behavior (7, 8)and enabling direct bloodborne transmission via injection drug use (IDU) (9). Consequently, quantifying the global burden attributable to drug use is critical for shaping effective public health interventions and advancing international goals, such as the World Health Organization (WHO)'s Global Health Sector Strategies (10).

The epidemiological links are well-established. IDU is a major driver of global epidemics of HIV, hepatitis B (HBV), and hepatitis C (HCV) (9). For instance, IDU is the leading risk factor for HCV in the United States and accounts for approximately 25% of new HIV cases worldwide (11, 12). This burden intensifies in resource-limited settings, such as parts of sub-Saharan Africa and Eastern Europe, where high-risk drug use intersects with overstretched health systems (13).

Despite this established connection, significant research gaps remain. Although previous Global Burden of Disease (GBD) studies have quantified the overall burden of drug use (14) or focused on specific regions (15, 16), a comprehensive, global analysis of the *infectious disease* burden specifically attributable to drug use is still lacking (17).

To address this gap, we present the first comprehensive analysis of the global burden of infectious diseases attributable to drug use from 1990 to 2021, with projections to 2036. Methodologically, we leverage the standardized GBD 2021 database and its cause-outcome hierarchy (18) and employ a suite of analytical models, including joinpoint regression, decomposition analysis, and Auto-Regressive Integrated Moving Average (ARIMA) forecasting (19). The objectives are to: (1) quantify changes in deaths and disability-adjusted life years (DALYs) burdens attributable to drug use from 1990 to 2021; (2) analyze the driving factors behind these changes, including population growth, aging, and epidemiological transitions; (3) evaluate health inequalities across regions based on the Socio-Demographic Index (SDI); and (4) project the trajectory of disease burden to 2036. The findings are intended to provide critical evidence for advancing the WHO’s Global Health Sector Strategies and the United Nations Sustainable Development Goal 3.3 (10), ultimately guiding resource allocation and stratified public health interventions.

## 2 Methods

### 2.1 Study overview

We analyzed the global, regional, and national burden of infectious diseases (HIV/AIDS and sexually transmitted infections (STIs); other infectious diseases including acute hepatitis B and C) attributable to drug use from 1990 to 2021 using the GBD 2021 database. The study included 204 countries and territories across 21 regions.

### 2.2 Data sources and processing

The data collection, standardization processes, and quality control methods for GBD 2021 have been detailed in previous studies (18). Data were extracted from the Global Health Data Exchange (GBD Results Tool: https://vizhub.healthdata.org/gbd-results/). We focused on risk-outcome pairs under the primary risk category of ‘Drug use’ for the following secondary outcomes: ‘HIV/AIDS and sexually transmitted infections’ (including tuberculosis-related subtypes) and ‘Other infectious diseases’ (including acute hepatitis B and C subtypes). Indicators included age-standardized mortality rate (ASMR), age-standardized DALYs rate (ASDR), and absolute numbers of deaths and DALYs, stratified by age, sex, and region.

### 2.3 Definition of risk factors and disease burden modeling

Drug use was defined according to *Diagnostic and Statistical Manual of Mental Disorders (DSM)* or the *International Classification of Diseases (ICD)* diagnostic criteria, encompassing opioid, amphetamine, and cocaine use disorders, and bloodborne infections from IDU. The burden was estimated using the GBD comparative risk assessment framework, calculating population attributable fractions (PAFs) based on relative risks and theoretical minimum risk exposure levels (TMREL). Detailed methods for PAF calculation, TMREL definition, and confounding adjustment are described in GBD methodology publications (15–17).

### 2.4 Statistical analysis

All statistical analyses were performed using R software (version 4.4.1) and Joinpoint Regression Software (version 5.4.0). For all estimates derived directly from the GBD database (e.g., death counts, DALYs, age-standardized rates), the 95% uncertainty intervals (UIs) provided by GBD were used. For metrics calculated in this study [e.g., estimated annual percentage change (EAPC)], 95% confidence intervals (CIs) were derived from the respective statistical models. A *p* < 0.05 was considered statistically significant. A graphical overview of the analytical framework is provided in Supplementary Figure 1. The complete R code is available in Supplementary Material 2, and technical details are described in the Supplementary Method 1.

#### 2.4.1 Trend analysis

We used the EAPC to quantify the average trend of age-standardized rates over time. A linear regression model was fitted to the natural logarithm of the rates, and the EAPC was derived from the model’s slope. The detailed formula and calculation steps are provided in Supplementary Method 1.1.

#### 2.4.2 Joinpoint regression analysis

Joinpoint regression was employed to identify significant turning points in temporal trends from 1990 to 2021. The model selection was based on the Bayesian Information Criterion (BIC), with a maximum of 5 joinpoints allowed. The Annual Percent Change (APC) for each segment and the Average Annual Percent Change (AAPC) over the entire period were calculated. The software parameters and model selection criteria are detailed in Supplementary Method 1.2.

#### 2.4.3 Health inequality analysis

We assessed socioeconomic-related health inequalities using the Slope Inequality Index (SII) and the Concentration Index (CI). The SII, an absolute measure, was calculated by regressing health outcomes against the relative rank of countries based on the SDI. The CI, a relative measure, was derived from the concentration curve. Comprehensive definitions and computational approaches for SII and CI are provided in Supplementary Method 1.3.

#### 2.4.4 Decomposition analysis

The Das Gupta method was applied to analyze the contributions of population growth, aging, and epidemiological changes to the trends in disease burden. This method addresses the interactions between these factors and provides a comprehensive decomposition of temporal changes in disease burden. The technical explanation of the decomposition procedure is included in Supplementary Method 1.4.

#### 2.4.5 Age-period-cohort analysis

The intrinsic estimator Age-Period-Cohort model was used to disentangle the effects of age, time period, and birth cohort on mortality. The model was fitted with 5-year age groups and 5-year periods. Model selection was based on the Akaike Information Criterion (AIC) and residual deviance. The model specification and parameter estimation details are available in Supplementary Method 1.5.

#### 2.4.6 Forecasting model

We utilized the ARIMA model to forecast future trends in age-standardized rates from 2022 to 2036. The model order (*p*, *d*, *q*) was automatically selected using the auto.arima function in R, which minimizes the AIC. The model’s stability was validated through out-of-sample testing. The technical explanation of the ARIMA model is included in Supplementary Method 1.6.

## 3 Results

### 3.1 Global trends in the burden of infectious diseases attributable to drug use from 1990 to 2021

Globally, from 1990 to 2021, the burden of infectious diseases attributable to drug use exhibited divergent trends for HIV/AIDS and STIs versus other infectious diseases (Table 1). The absolute number of deaths and DALYs from HIV/AIDS and STIs increased substantially, by 174 and 159%, respectively. Conversely, the number of deaths and DALYs from other infectious diseases declined by 47 and 43%. While males carried a higher absolute burden for HIV/AIDS and STIs, the ASDR and ASMR among women showed significantly higher growth rates between 1990 and 2021.

**Table 1:** Global all-ages numbers and age-standardized rates of deaths and DALYs for infectious diseases attributable to drug use in 1990 and 2021, according to sex.

Characteristics		HIV/AIDS and sexually transmitted infections			Other infectious diseases		
		Year		Percentage change 1990–2021 (×100%)	Year		Percentage change 1990–2021 (×100%)
		1990	2021		1990	2021	
All-ages numbers							
Deaths	Total	16,626 (13,626 ~ 20,456)	67,473 (56,923 ~ 81,382)	1.74 (1.29 ~ 2.36)	2,574 (1,039 ~ 5,193)	2037 (946 ~ 3,426)	−0.47 (−0.64 ~ −0.13)
	Male	13,231 (11,040 ~ 5,846)	48,625 (41,432 ~ 58,527)	1.49 (1.07 ~ 2.08)	1916 (616 ~ 4,138)	1,077 (587 ~ 1915)	−0.62 (−0.79 ~ −0.2)
	Female	3,395 (2,507 ~ 4,693)	18,848 (14,678 ~ 26,214)	2.74 (1.88 ~ 4.02)	658 (133 ~ 1,361)	960 (277 ~ 1961)	−0.02 (−0.43 ~ 0.98)
DALYs	Total	885,194 (725,219 ~ 1,078,112)	3,388,519 (2,873,881 ~ 4,097,364)	1.59 (1.2 ~ 2.12)	111,306 (46,150 ~ 224,190)	93,567 (43,672 ~ 160,553)	−0.43 (−0.61 ~ −0.08)
	Male	702,774 (584,594 ~ 837,917)	2,402,936 (2,045,810 ~ 2,881,741)	1.32 (0.98 ~ 1.78)	82,041 (27,454 ~ 175,327)	50,172 (27,345 ~ 92,229)	−0.59 (−0.77 ~ −0.15)
	Female	182,420 (134,430 ~ 250,103)	985,583 (764,328 ~ 1,386,262)	2.64 (1.82 ~ 3.83)	29,265 (6,148 ~ 58,517)	43,395 (13,497 ~ 91,544)	0 (−0.4 ~ 1.04)
Age-standardized rates							
ASMR	Total	0.34 (0.28 ~ 0.42)	0.8 (0.68 ~ 0.96)	1.35 (0.96 ~ 1.87)	0.06 (0.02 ~ 0.11)	0.02 (0.01 ~ 0.04)	−0.57 (−0.71 ~ −0.31)
	Male	0.54 (0.44 ~ 0.64)	1.15 (0.98 ~ 1.39)	1.16 (0.8 ~ 1.67)	0.09 (0.03 ~ 0.18)	0.03 (0.01 ~ 0.05)	−0.7 (−0.84 ~ −0.35)
	Female	0.14 (0.1 ~ 0.19)	0.45 (0.35 ~ 0.63)	2.21 (1.46 ~ 3.3)	0.03 (0.01 ~ 0.06)	0.02 (0.01 ~ 0.05)	−0.2 (−0.52 ~ 0.59)
ASDR	Total	17.77 (14.59 ~ 21.66)	40.49 (34.35 ~ 49.03)	1.28 (0.94 ~ 1.74)	2.34 (0.96 ~ 4.69)	1.13 (0.52 ~ 1.94)	−0.52 (−0.67 ~ −0.22)
	Male	27.91 (23.3 ~ 33.24)	57.16 (48.67 ~ 68.54)	1.05 (0.75 ~ 1.46)	3.48 (1.15 ~ 7.46)	1.21 (0.66 ~ 2.24)	−0.65 (−0.81 ~ −0.29)
	Female	7.35 (5.48 ~ 10.07)	23.77 (18.35 ~ 33.46)	2.23 (1.52 ~ 3.27)	1.21 (0.25 ~ 2.4)	1.04 (0.33 ~ 2.23)	−0.14 (−0.48 ~ 0.74)

DALYs, disability-adjusted life years. ASMR, age-standardized mortality rate. ASDR, age-standardized DALYs rate. All-ages numbers of deaths and DALYs were shown as number (95% UIs). ASMR and ASDR were shown as rates (95% UIs, per 100,000 population). Percentage change from 1990 to 2021 were shown as rates (95% UIs, per 100,000 population).

### 3.2 Global trends by gender and age groups

**Figure 1:**
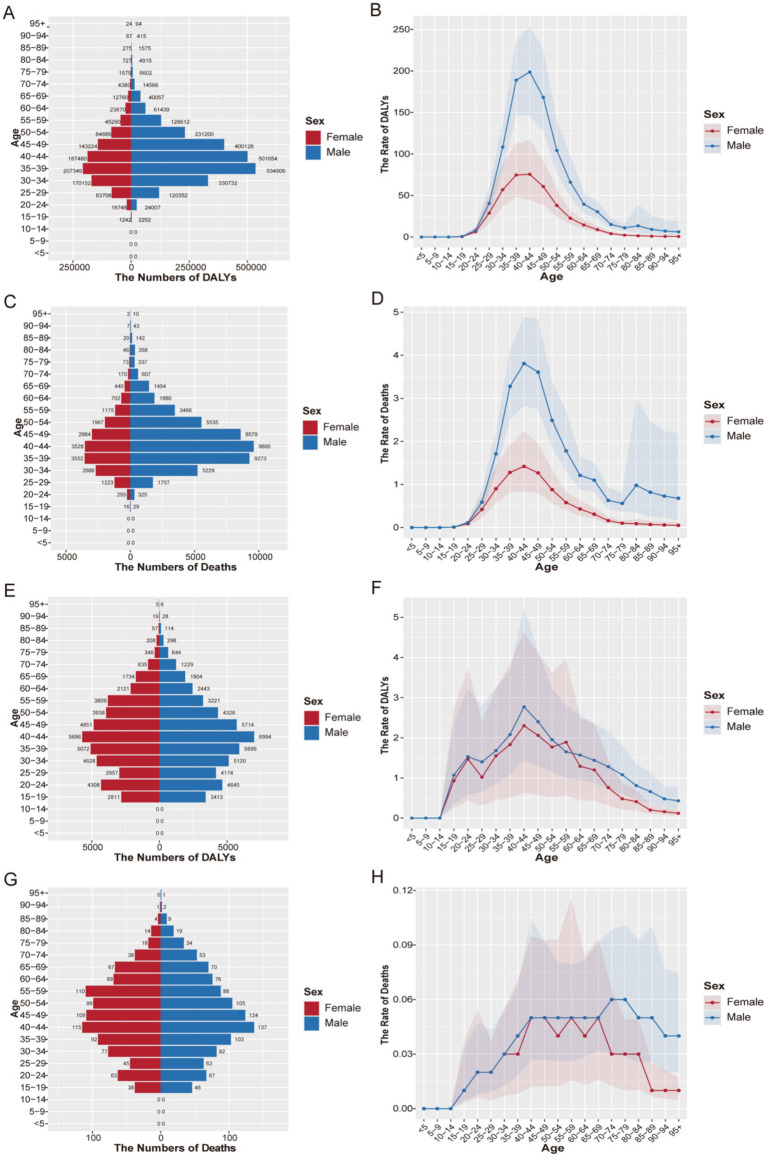
Age-specific DALYs and deaths numbers and ASDR and ASMR of infectious diseases in Global, 2021. **(A)** HIV/AIDS and STIs: age-specific DALYs number, **(B)** ASDR (per 100,000 population), **(C)** age-specific deaths number, **(D)** ASMR (per 100,000 population). **(E)** Other infectious diseases: age-specific DALYs number, **(F)** ASDR (per 100,000 population), **(G)** age-specific deaths number, **(H)** ASMR (per 100,000 population). DALYs, disability-adjusted life years.

In 2021, the disease burden exhibited distinct gender and age patterns (Figure 1). For HIV/AIDS and STIs, DALYs and deaths peaked sharply among males aged 35–39 years (Figures 1A,C). The age-standardized rates (ASDR and ASMR) were consistently higher in males across all age groups, with the most pronounced disparity observed between 30 and 54 years of age (Figures 1B,D). For other infectious diseases, the burden also peaked in middle adulthood (40–44 years) (Figures 1E–G). Notably, the ASMR for these diseases widened considerably after age 65–69, with rates remaining elevated in males but declining in females (Figure 1H).

### 3.3 Global trends by year

**Figure 2:**
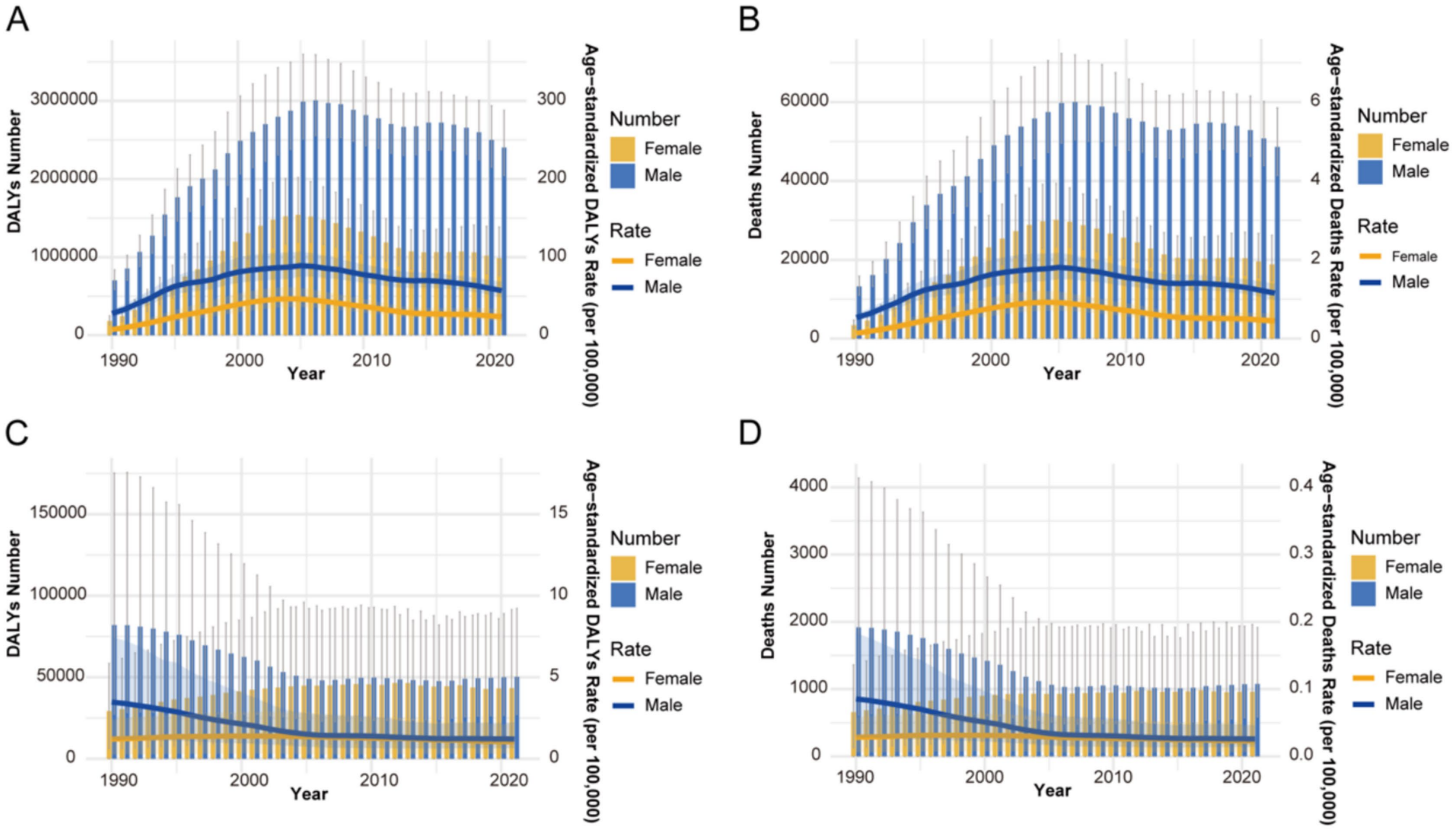
Trends in the all-age cases and ASDR, and ASMR of infectious diseases by sex from 1990 to 2021. **(A)** HIV/AIDS and STIs: DALYs number and rate (per 100,000 population), **(B)** deaths number and rate (per 100,000 population). **(C)** Other infectious diseases: DALYs number and rate (per 100,000 population), **(D)** deaths number and rate (per 100,000 population). DALYs, disability-adjusted life years.

Temporal trends from 1990 to 2021 revealed a characteristic pattern for HIV/AIDS and STIs, with age-standardized rates initially rising to a peak around 2005, followed by a sustained decline (Figures 2A,B). In contrast, the burden of other infectious diseases demonstrated a steady downward trend over the same period (Figures 2C,D). Gender disparities persisted throughout but narrowed over time for other infectious diseases.

### 3.4 Joinpoint regression analysis of the burden of infectious diseases attributable to drug use risk factors

**Figure 3:**
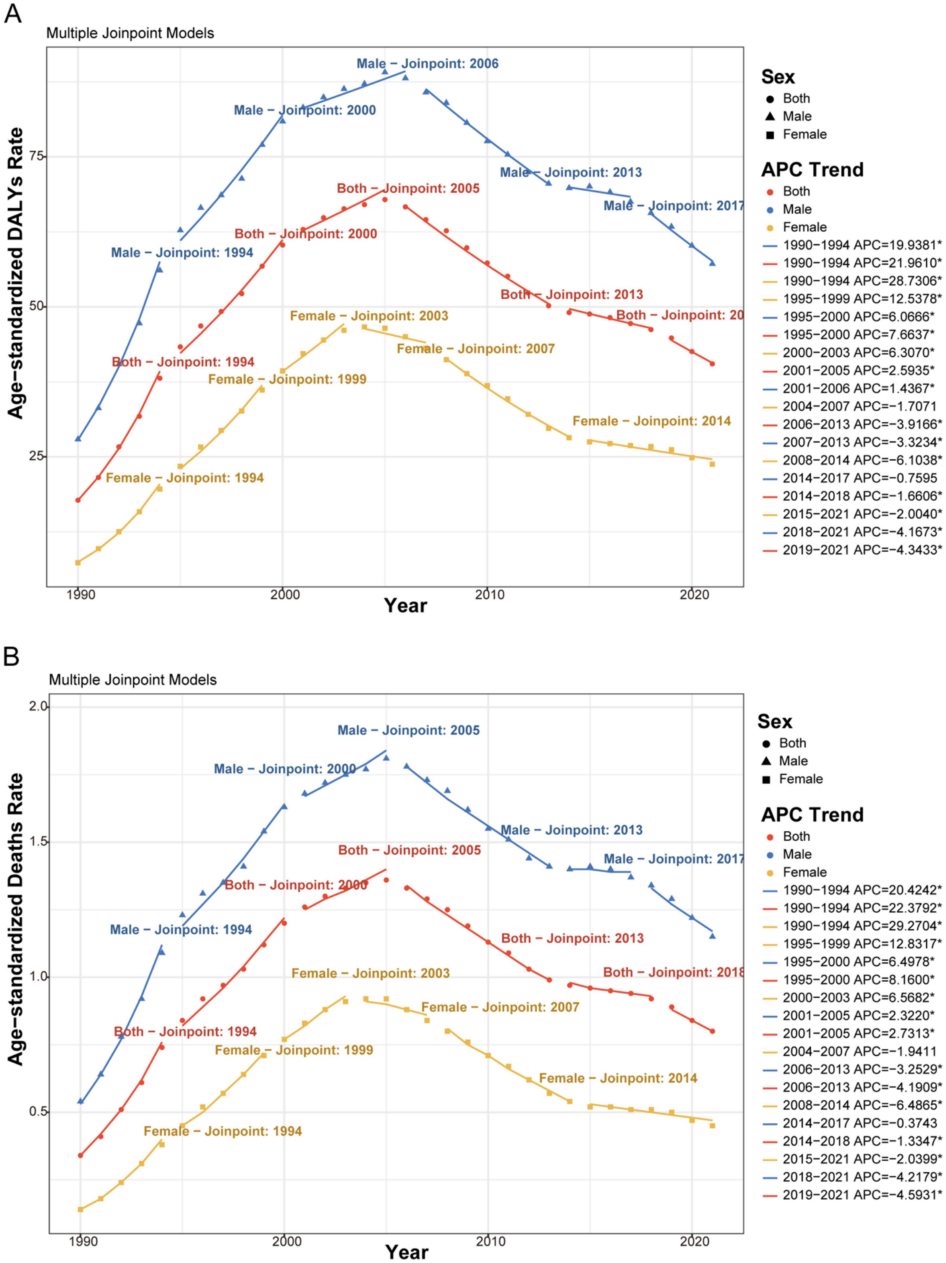
Joinpoint regression analysis of the sex-specific ASDR and ASMR for HIV/AIDS and STIs attributable to drug use risk factors in Global from 1990 to 2021. **(A)** ASDR (per 100,000 population). **(B)** ASMR (per 100,000 population). APC: Annual Percent Change. *Indicates that the APC is significantly different from zero at the alpha = 0.05 level.

**Figure 4:**
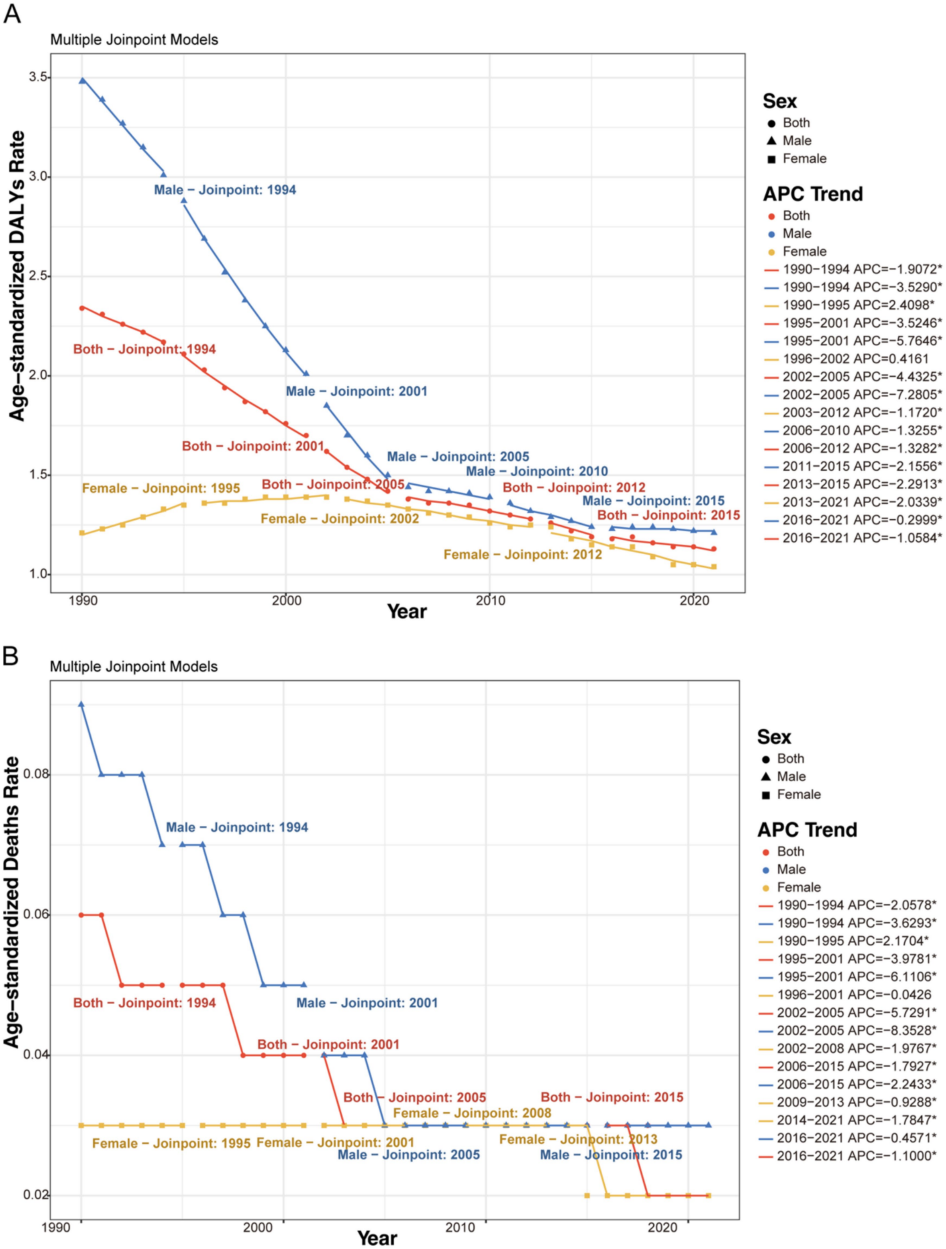
Joinpoint regression analysis of the sex-specific ASDR and ASMR for other infectious diseases attributable to drug use risk factors in Global from 1990 to 2021. **(A)** ASDR (per 100,000 population). **(B)** ASMR (per 100,000 population). APC, Annual Percent Change. *Indicates that the APC is significantly different from zero at the alpha = 0.05 level.

Joinpoint analysis identified significant turning points in the trends of age-standardized rates (Figures 3, 4 and Supplementary Table 1). For HIV/AIDS and STIs, the most rapid increase in ASDR occurred between 1990 and 1994. A consistent declining trend commenced after 2005 for both sexes. For other infectious diseases, the overall trend was decreasing, with the most rapid decline in ASDR occurring during 2001–2005 for males and 2012–2021 for females.

### 3.5 National burdens of infectious diseases attributable to drug use risk factors

**Figure 5:**
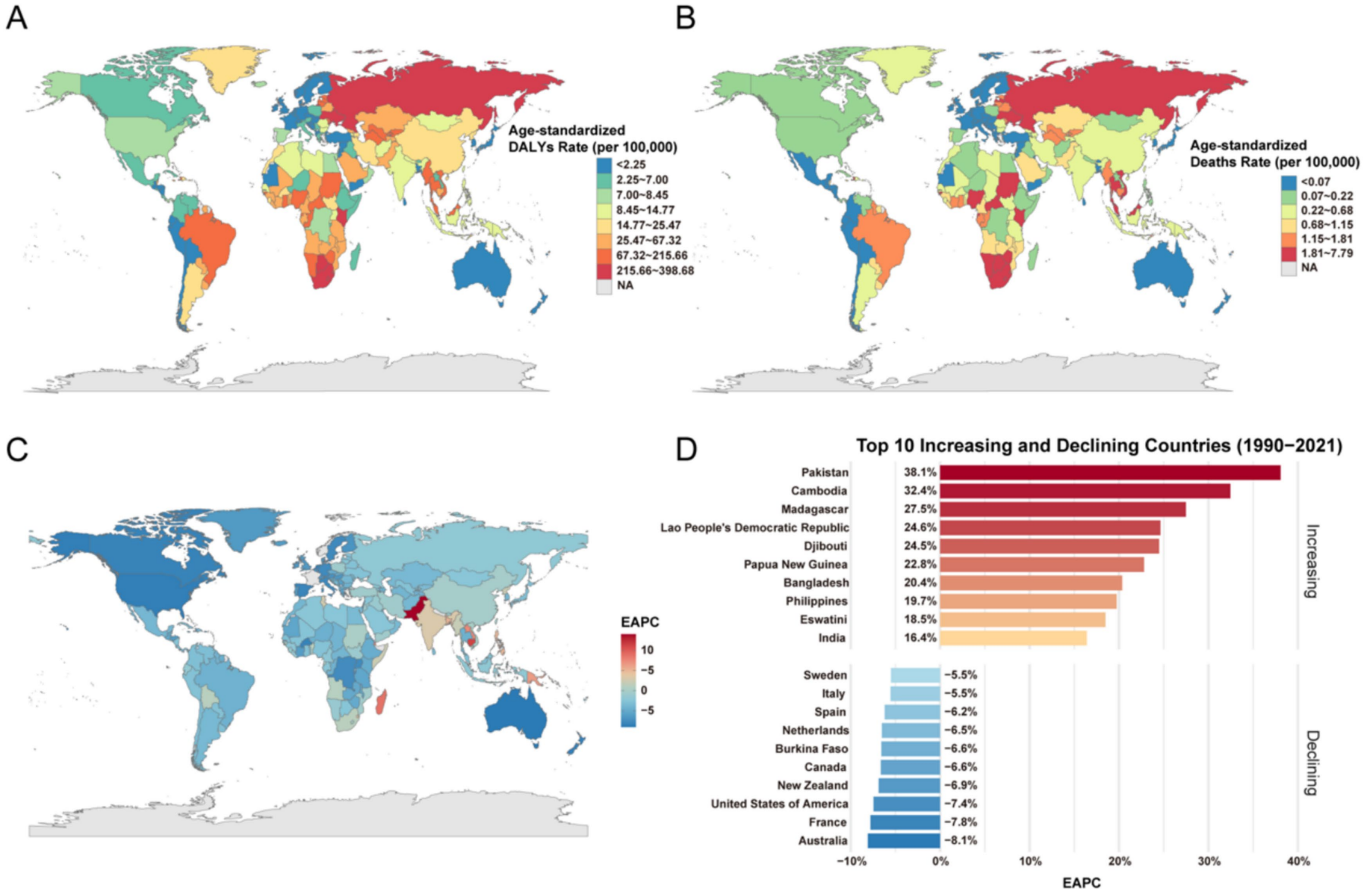
ASDR, ASMR, and EAPC for HIV/AIDS and STIs attributable to drug use risk factors across different countries in 2021. **(A)** ASDR (per 100,000 population). **(B)** ASMR (per 100,000 population). **(C)** The EAPC in ASMR for HIV/AIDS and STIs attributable to drug use risk factors across different countries from 1990 to 2021. **(D)** Top 10 increasing and declining countries of EAPC in ASMR from 1990 to 2021 across different countries. DALYs, disability-adjusted life years. EAPC, estimated annual percentage change.

**Figure 6:**
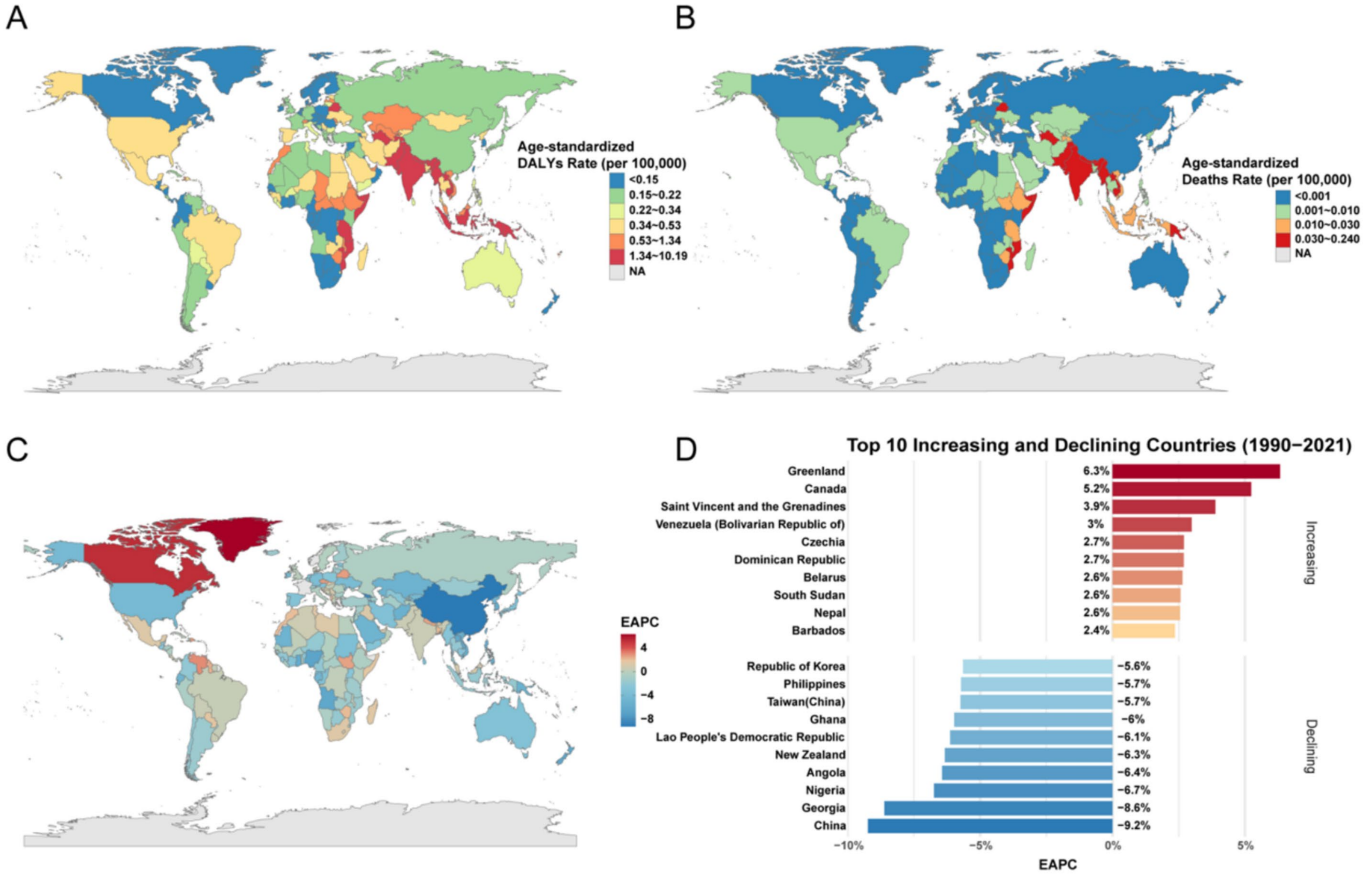
ASDR, ASMR, and EAPC for other infectious diseases attributable to drug use risk factors across different countries in 2021. **(A)** ASDR (per 100,000 population). **(B)** ASMR (per 100,000 population). **(C)** The EAPC in ASMR for other infectious diseases attributable to drug use risk factors across different countries from 1990 to 2021. **(D)** Top 10 increasing and declining countries of EAPC in ASMR across different countries from 1990 to 2021. DALYs, disability-adjusted life years. EAPC, estimated annual percentage change.

The national distribution of the burden in 2021 and its temporal change were highly heterogeneous (Figures 5, 6). Russia recorded the highest national ASDR for HIV/AIDS and STIs, whereas Brunei had the lowest (Figure 5A). Lesotho exhibited the highest ASMR, while Albania, Bosnia and Herzegovina, Brunei, Comoros, Japan, South Korea, North Macedonia, Mauritania, Sao Tome and Principe, and Slovakia had the lowest ASMR (Figure 5B). The most rapid increases in ASMR from 1990 to 2021 were observed in Pakistan, Cambodia, and Madagascar, while the steepest declines were seen in Australia, France, and the United States. For other infectious diseases, the highest ASDR and ASMR were observed in Tonga (Figures 5C,D). For other infectious diseases, the highest ASDR and ASMR were observed in Tonga (Figures 6A,B).

### 3.6 Burden of infectious diseases attributable to drug use risk factors by SDI

**Figure 7:**
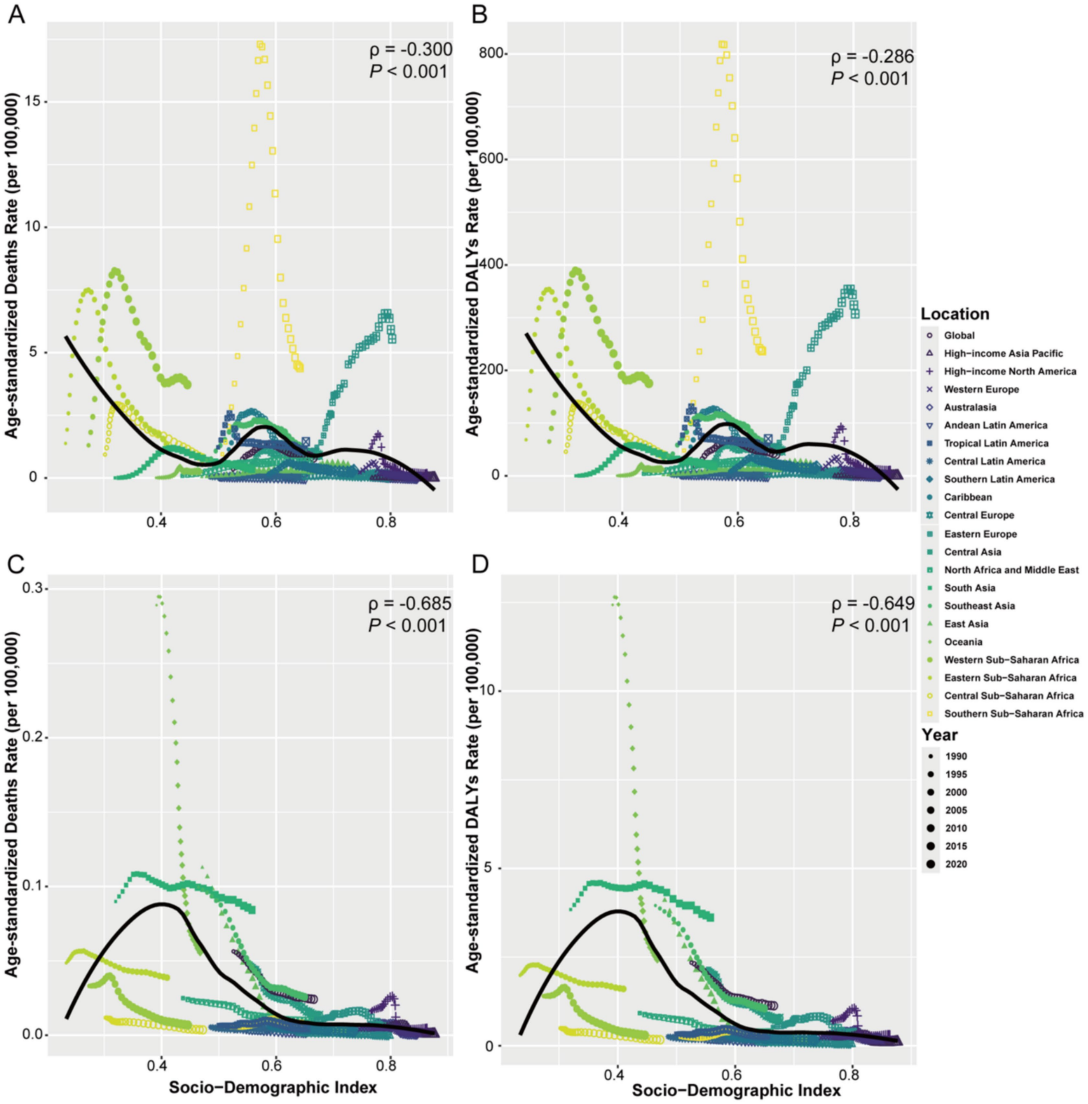
Relationship between SDI and ASMR/ASDR of infectious diseases attributable to drug use risk factors by Global and 21 GBD Regions from 1990 to 2021. **(A)** HIV/AIDS and STIs: ASMR (per 100,000 population), **(B)** ASDR (per 100,000 population). **(C)** Other infectious diseases: ASMR (per 100,000 population), **(D)** ASDR (per 100,000 population). For each region, the points from left to right represent the estimated values for each year from 1990 to 2021, while the expected values are shown as a black line. SDI, socio-demographic index. DALYs, disability-adjusted life years.

The SDI was found to be negatively correlated with the ASMR of HIV/AIDS and STIs attributable to drug use risk factors (*ρ* = −0.300, *p* < 0.001) (Figure 7A). From 1990 to 2021, Global, Central Sub-Saharan Africa, South Asia, Southeast Asia, Western Europe, Tropical Latin America, High-income North America, and Caribbean regions generally followed the expected trend of ASMR from 1990 to 2021. Oceania, North Africa and Middle East, East Asia, Central Asia, Southern Latin America, Andean Latin America, Central Latin America, Central Europe, High-income Asia Pacific, and Australasia remained below the expected levels, with almost no changes in ASMR. In contrast, Southern Sub-Saharan Africa, Western Sub-Saharan Africa, Eastern Sub-Saharan Africa, and Eastern Europe were far above the expected levels, with fluctuating ASMR. Similar trends were observed in ASDR (Figure 7B).

For other infectious diseases, the SDI was also significantly negatively correlated with ASMR (*ρ* = −0.685, *p* < 0.001) (Figure 7C). Most regions followed trends similar to or below the expected trajectory, while Oceania, South Asia, East Asia, and Southeast Asia were above the expected trend, with Oceania being the most prominent. However, as the SDI increased, the ASMR in Oceania declined rapidly. Similar trends were observed in ASDR (Figure 7D).

### 3.7 Cross-nation health inequality in the burden of infectious diseases attributable to drug use risk factors

**Figure 8:**
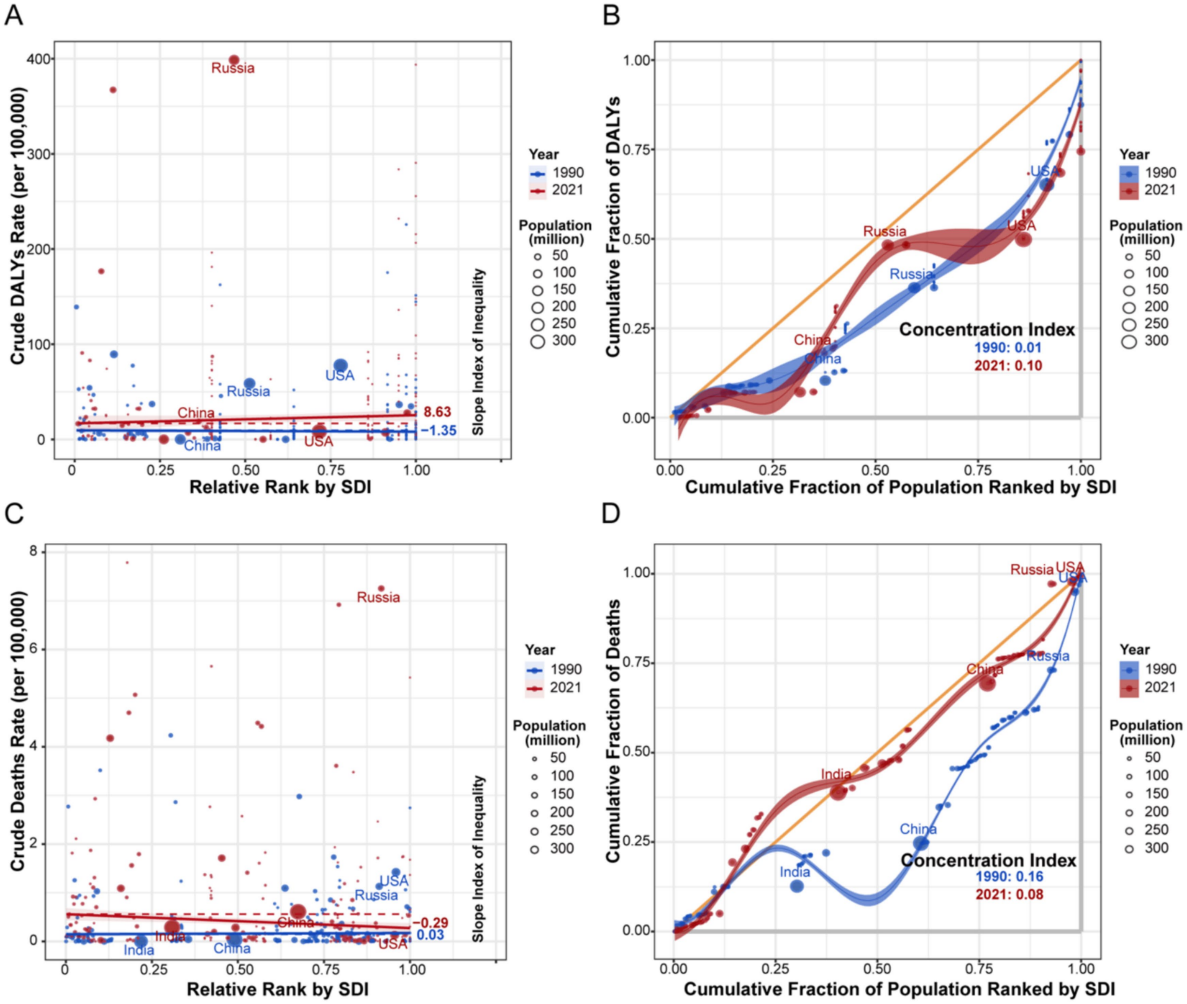
Analysis of SII and CI for the burden of HIV/AIDS and STIs attributable to drug use risk factors across different countries or regions from 1990 to 2021. **(A)** SII analysis of DALYs (per 100,000 population). **(B)** CI analysis of DALYs (per 100,000 population). **(C)** SII analysis of deaths (per 100,000 population). **(D)** CI analysis of deaths (per 100,000 population). SII, slope inequality index. CI, concentration index. SDI, socio-demographic index. DALYs, disability-adjusted life years.

**Figure 9:**
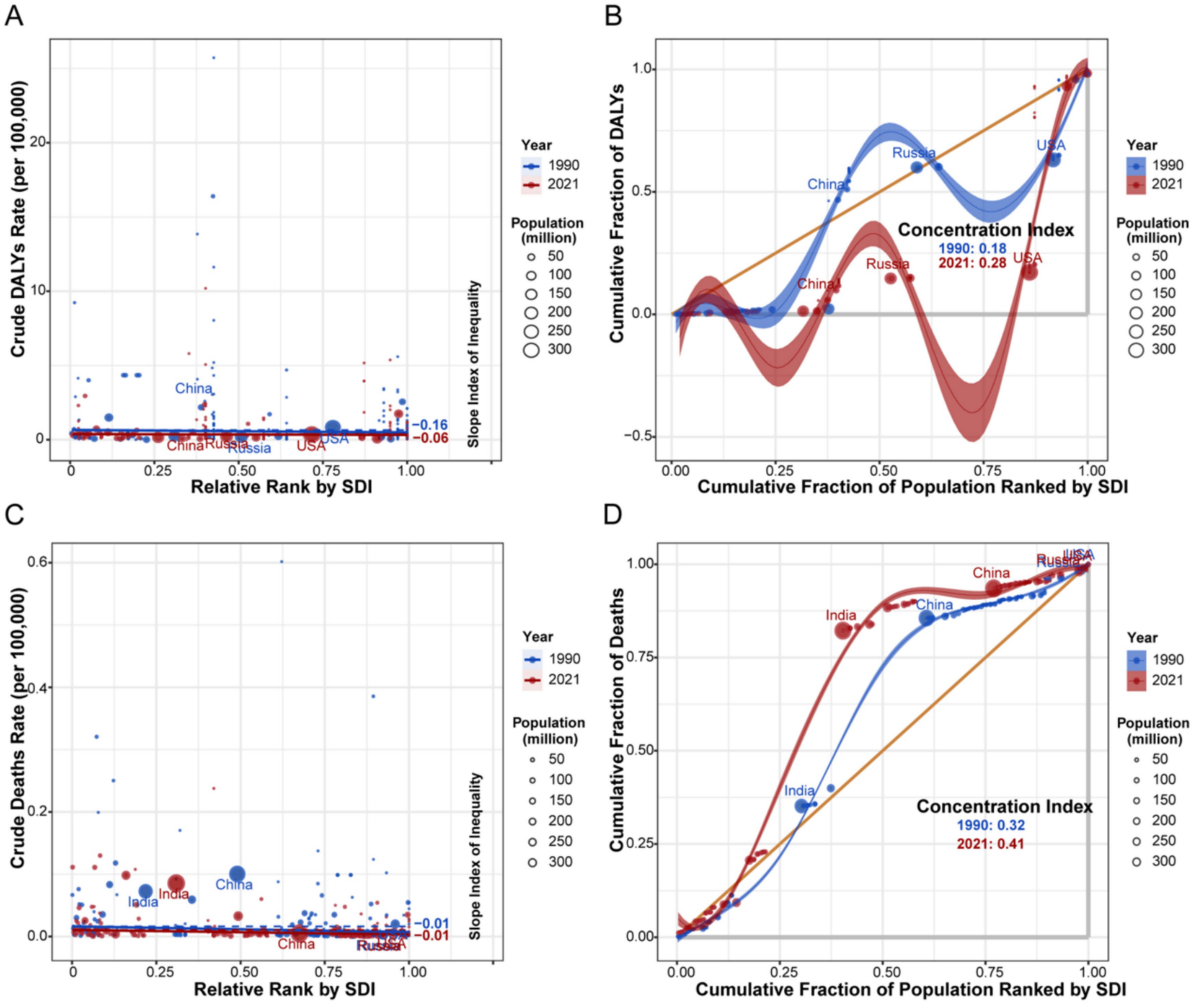
Analysis of SII and CI for the burden of other infectious diseases attributable to drug use risk factors across different countries or regions from 1990 to 2021. **(A)** SII analysis of DALYs (per 100,000 population). **(B)** CI analysis of DALYs (per 100,000 population). **(C)** SII analysis of deaths (per 100,000 population). **(D)** CI analysis of deaths (per 100,000 population). SII, slope inequality index. CI, concentration index. SDI, socio-demographic index. DALYs, disability-adjusted life years.

Health inequality metrics revealed a dynamic shift in the socioeconomic distribution of the burden between 1990 and 2021 (Figures 8, 9). For HIV/AIDS and STIs, the SII for DALYs transitioned from negative to positive, indicating a shift in burden concentration from low-SDI to high-SDI countries. In contrast, the burden of other infectious diseases, as measured by the CI for DALYs, remained disproportionately concentrated in low-SDI nations.

### 3.8 Decomposition of burden drivers for infectious diseases attributable to drug use risk factors

**Figure 10:**
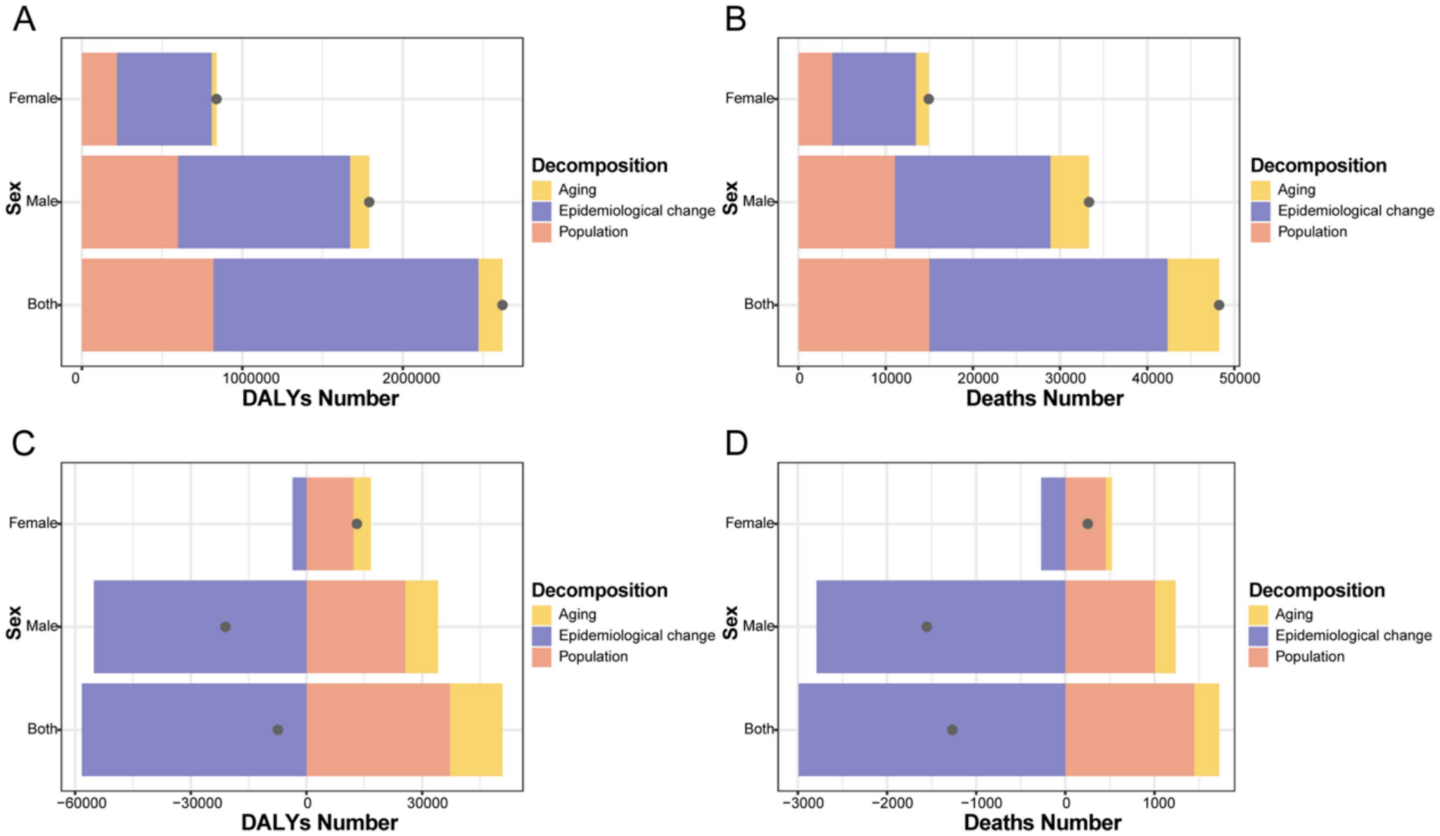
Changes in DALYs and deaths from infectious diseases according to population-level determinants of population growth, aging, and epidemiological change by sex at the Global level from 1990 to 2021. The gray dot represents the overall value of change contributed by all 3 components. For each component, the magnitude of a positive value indicates a corresponding increase in infectious diseases DALYs or deaths attributed to the component; the magnitude of a negative value indicates a corresponding decrease in infectious diseases DALYs or deaths attributed to the related component. **(A)** HIV/AIDS and STIs: DALYs number, **(B)** deaths number. **(C)** Other infectious diseases: DALYs number, **(D)** deaths number. DALYs, disability-adjusted life years.

From 1990 to 2021, the global increase in DALYs from HIV/AIDS and STIs attributable to drug use was primarily driven by epidemiological changes, which accounted for 62.95% of the total absolute effect. Population growth and aging contributed 31.32 and 5.73%, respectively, to this increase (Supplementary Table 2 and Figures 10A,B).

For other infectious diseases, the global reduction in DALYs masked significant sex-based disparities. Among males, the substantial decrease in DALYs was predominantly driven by favorable epidemiological changes, accounting for 61.77% of the total absolute effect. In contrast, females experienced an increase in DALYs, which was largely attributable to demographic pressures—population growth (60.13%) and aging (22.12%)—that overwhelmed the protective effect of epidemiological improvements (−17.76%). A similar pattern of drivers was observed for deaths, with epidemiological changes contributing most substantially to reductions in male mortality, while demographic factors drove increased mortality among females (Supplementary Table 3 and Figures 10C,D).

### 3.9 Age-period-cohort effects on infectious diseases attributable to drug use risk factors mortality

**Figure 11:**
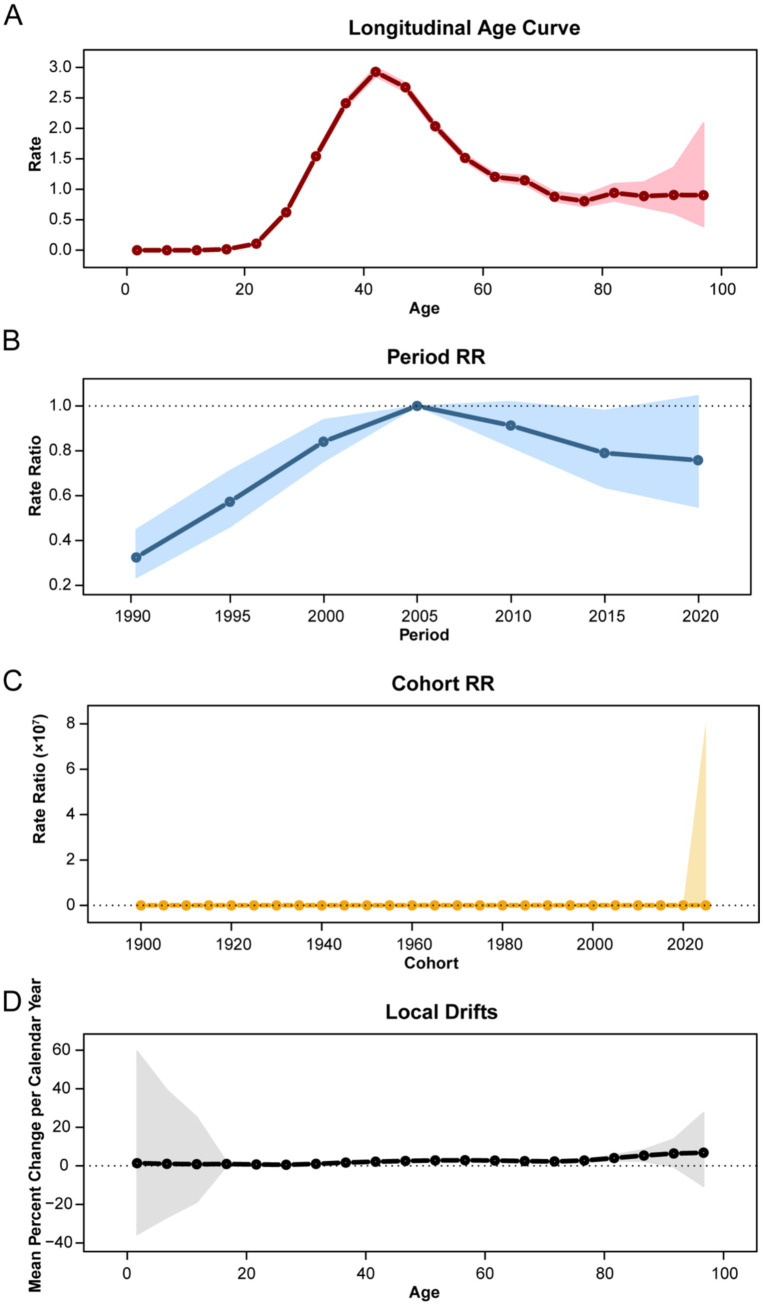
Age-period-cohort analysis of the mortality burden of global infectious diseases attributable to drug use risk (combining deaths from HIV/AIDS and STIs, and other infectious diseases). **(A)** Longitudinal age curve: age effect on mortality rate (per 100,000 population). **(B)** Period relative rate: period effect on mortality rate (per 100,000 population). **(C)** Cohort relative rate: cohort effect on mortality rate (per 100,000 population). **(D)** Local drifts: mean percent change in mortality rate per calendar year compared to the previous year (per 100,000 population).

The Age-Period-Cohort analysis demonstrated distinct effects on mortality risk (Figure 11 and Supplementary Table 4). The age effect indicated that mortality risk peaked in the 40–44 age group. The period effect showed that mortality risk peaked in the 2002–2006 period and subsequently declined. The cohort effect revealed that relative risks increased for birth cohorts up to approximately 1962, after which a decline was observed. Local drift analysis shows changes in mortality rates compared to the previous year across different age groups. Before the 35–39 age group, mortality rates decreased compared to the previous year, but starting from the 40–44 age group, mortality rates increased compared to the previous year.

### 3.10 Future burden projections for infectious diseases attributable to drug use risk factors

**Figure 12:**
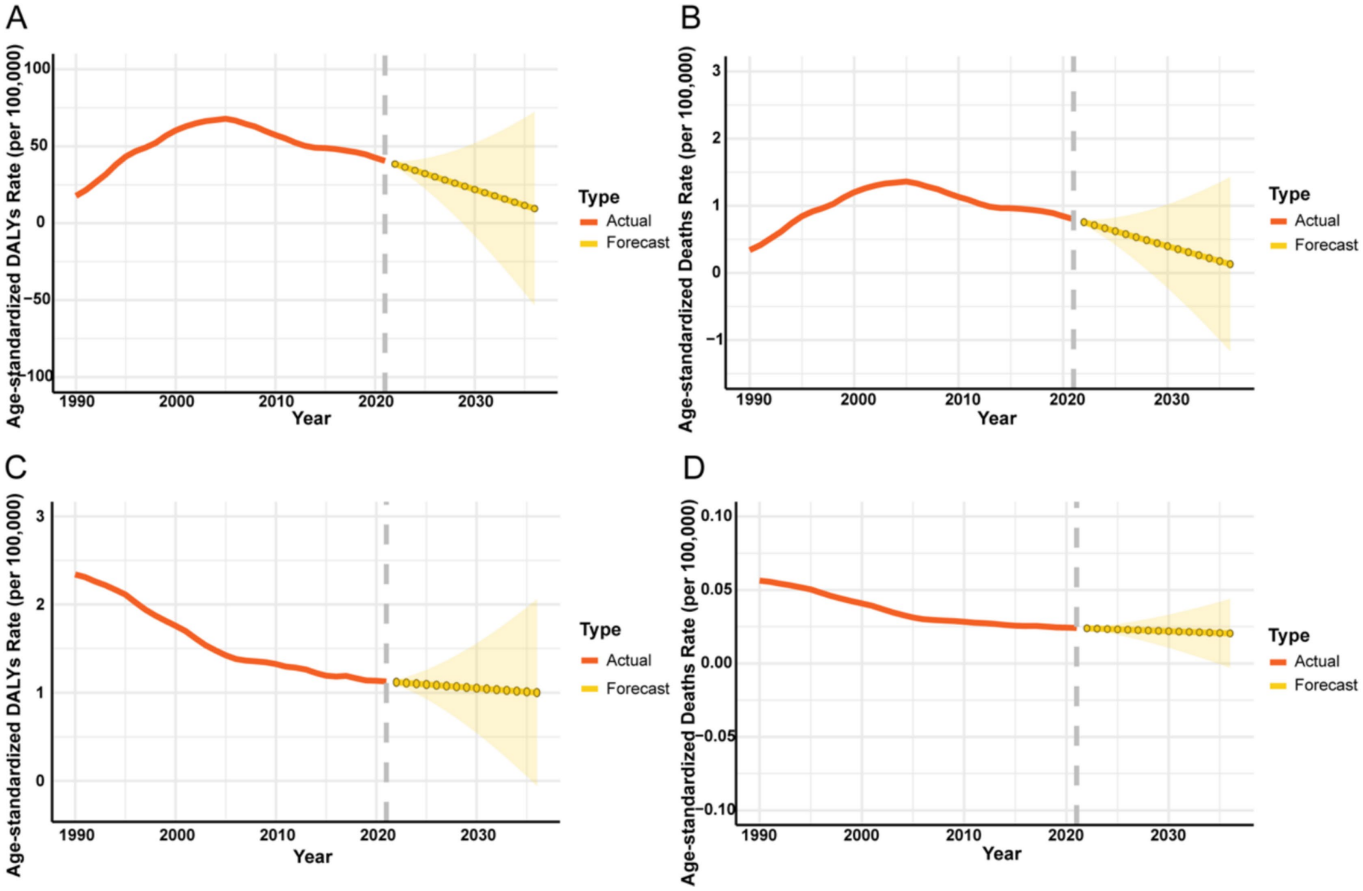
Projected trends of ASDR and ASMR for global infectious diseases attributable to drug use risk factors over the next 15 years (2022–2036). The red line represents the actual trend of ASDR and ASMR for infectious diseases attributable to drug use risk factors from 1990 to 2021; the yellow dashed line and shaded area represent the forecasted trend and its 95% CIs. **(A)** HIV/AIDS and STIs: ASDR (per 100,000 population), **(B)** ASMR (per 100,000 population). **(C)** Other infectious diseases: ASDR (per 100,000 population), **(D)** ASMR (per 100,000 population). DALYs, disability-adjusted life years.

Forecasts from the ARIMA models projected a substantial decline in the global burden of HIV/AIDS and STIs attributable to drug use by 2036 (Figure 12). The ASDR and ASMR are projected to decline by 75.3 and 82.9%, respectively, from 2022 to 2036. In contrast, the ASDR and ASMR for other infectious diseases are forecast to remain stable or exhibit a slight decrease over the same period.

## 4 Discussion

This study presents the first comprehensive analysis of the global burden of infectious diseases attributable to drug use from 1990 to 2021, integrating trend analysis, health inequality assessment, and future projections. Our findings reveal a critical epidemiological divergence: a substantial increase in the burden of HIV/AIDS and STIs contrasted with a significant decline in other infectious diseases, primarily acute viral hepatitis. This divergence was paralleled by a notable shift in the socioeconomic distribution of the HIV/AIDS and STIs burden from low- to high-SDI countries, whereas other infectious diseases remained concentrated in low-SDI regions. Decomposition analysis indicated these trends were predominantly driven by epidemiological changes, moderated by distinct demographic forces and gendered impacts.

The observed transition of HIV/AIDS and STIs burden toward high-SDI countries, as evidenced by the shift in the SII for DALYs from negative to positive, underscores a shift in the risk environment, likely fueled by the opioid crisis involving potent synthetics like fentanyl in regions such as North America and Eastern Europe (20–22). In these settings, epidemics have evolved within structural contexts of limited access to harm reduction services, such as needle-syringe programs (NSPs) and opioid agonist therapy (OAT) (22, 23). Conversely, the scale-up of antiretroviral therapy (ART) in many low- and middle-income countries has likely averted deaths, moderating the absolute burden despite persistent transmission risks (24, 25). The significant burden among males aged 30–54 aligns with the typical duration of injection drug use and higher engagement in IDU and high-risk sexual behaviors (8, 26). The highest mortality rates for other infectious diseases in men aged 65–69 may be attributed to the cumulative effects of long-term infections, comorbidities, and poorer healthcare engagement in older adults (27, 28). The rising burden among women, though from a lower baseline, highlights unique vulnerabilities, including biological susceptibility and systemic barriers to healthcare access (29, 30).

Our forecasts project an encouraging, substantial decline in the burden of HIV/AIDS and STIs by 2036, with the ASDR and ASMR projected to fall by 75.3 and 82.9%, respectively. This optimistic trajectory likely reflects the cumulative impact of decades of public health investment, including expanded ART, pre-exposure prophylaxis (PrEP), and harm reduction efforts (31). However, this projected decline must be interpreted with caution. While it signifies tremendous progress, it also implies that the ambitious global target to eliminate HIV/AIDS and viral hepatitis as public health threats by 2030 (32) will be extraordinarily difficult to achieve universally. The persistence of the burden, even at a lower level, signals the tenacity of the syndemic between drug use and infectious diseases, particularly in the face of evolving drug markets and ongoing socioeconomic disparities.

For other infectious diseases, our projection of a slowly declining ASDR and a stabilized, low ASMR suggests that current interventions for diseases like acute hepatitis B and C are having a stabilizing effect (33). However, the lack of a rapid decline indicates that prevention measures (e.g., hepatitis B vaccination, safe injection equipment) are not being deployed at the scale or intensity required to match the persistence of risk behaviors, particularly in specific geographic hotspots and demographic groups (34).

To accelerate progress toward elimination goals, a stratified and politically engaged public health response is imperative. High-SDI countries must intensify efforts to regulate the pharmaceutical and illicit opioid supply and expand evidence-based harm reduction services to match the scale of the need (35, 36). Low- and middle-SDI regions should prioritize integrating “test-and-treat” strategies for HIV and HCV with scalable interventions like hepatitis B vaccination, OAT, and sterile syringe access (37–39). The gendered burden necessitates targeted interventions for women, including gender-sensitive harm reduction services and improved access to sexual and reproductive healthcare. Furthermore, strengthening social support systems (40, 41). Cross-sector collaboration is vital to enhance disease surveillance networks, ensuring comprehensive access to “prevention-treatment-social support” services (19, 42). Emerging technologies, including HIV self-testing, point-of-care HCV RNA testing, and telehealth for harm reduction service delivery, offer promising avenues to overcome traditional barriers to access and engagement (43–45).

This study has several limitations. First, as with all GBD-based analyses, our estimates are subject to the uncertainty inherent in the modeling process, particularly for regions with limited primary surveillance data (46). Second, the identification of drug use, especially IDU, relies on model-based estimates and may be affected by underreporting and misclassification. Third, the ecological design demonstrates association but cannot establish causality. Fourth, the ARIMA projections assume temporal stability and do not account for potential future policy changes or structural breaks, which could alter the projected trajectories.

## 5 Conclusion

This study delineates the evolving landscape of drug use-related infectious diseases, revealing a sharp increase in HIV/AIDS and STIs burden alongside a decline in other infectious diseases. Young adult males remain the core risk group, but the burden on females is rising more rapidly. Health inequality analysis highlights the dynamic relationship between economic development and risk environments. These findings underscore the need for politically engaged, stratified public health strategies that address both supply and demand sides of drug use, tailored to local epidemiological contexts and SDI levels, to mitigate disparities and advance global health equity.

## Data Availability

The original contributions presented in the study are included in the article/Supplementary material, further inquiries can be directed to the corresponding author.
