# Supplementary material for: Global burden and forecast of infectious diseases attributable to drug use: evidence from GBD 2021
Source: Front Public Health. 2025 Dec 15;13:1706764. doi: 10.3389/fpubh.2025.1706764 (PMC12745377; doi:10.3389/fpubh.2025.1706764)
Supplement: Supplementary file 1 [file Data_Sheet_1.DOCX]

- 1. **Trend Analysis**

The EAPC is calculated based on a linear regression model. The basic model assumes that mortality rates change exponentially over time: $y=y_{0}\times e^{\beta t}$ where *y* represents the indicator value (e.g., mortality rate) at time *t*, *y*_0_ is the initial value, and *β* is the regression coefficient representing the annual rate of change. To transform the model into a linear relationship, the natural logarithm is applied: $\ln\left( y \right)=\ln\left( y_{0} \right)+\beta t$. The slope *β* is obtained through linear regression fitting. Finally, EAPC is calculated using the formula: $EAPC=(e^{\beta}-1)\times100\%$ . If *β* > 0, it indicates an average annual increase; if *β* < 0, it indicates an average annual decrease. The 95% CIs is derived from the linear regression model, and the strength of the trend is determined by whether the 95% CIs crosses zero.

The association between the SDI and EAPC is assessed using Spearman's rank correlation analysis (denoted as *ρ*), which measures the strength and direction of the monotonic relationship between two variables. The range of *ρ* is -1 to +1: if *ρ* > 0, it indicates a completely monotonic increasing relationship; if *ρ* < 0, it indicates a completely monotonic decreasing relationship; if = 0, it indicates no monotonic relationship (though nonlinear relationships may exist). Effect size classification: *ρ* between 0.1 and 0.3: weak correlation; *ρ* between 0.3 and 0.5: moderate correlation; *ρ* > 0.5: strong correlation.

- 1. **Joinpoint Regression Analysis**

Software & Parameters: Specify “Joinpoint Desktop Software (v5.4.0.1) from the National Cancer Institute”. Model Settings: State “Monte Carlo permutation test with 4499 permutations and an overall alpha level of 0.05”.Selection Criterion: Mention “Model selected based on the lowest Bayesian Information Criterion (BIC)”.

- 1. **Health Inequality Analysis (SII and CI)**

The SII and CI were used to analyze the socioeconomic absolute and relative inequalities in the infectious disease burden caused by drug use as risk factors across different countries or regions. The SII is an absolute measure of health inequality, representing the health gap between the most disadvantaged and the most advantaged groups. The key steps for constructing the SII are as follows: First, countries are ranked based on their SDI, and the cumulative population proportion is calculated; Secondly, a weighted regression model was established between the DALYs or deaths caused by infectious diseases attributable to drug use risk factors and the SDI ranking of countries. The SII value, which represents the slope of the regression line, reflects the absolute gradient difference in disease burden across varying levels of SDI. A positive SII value indicates that the disease burden is disproportionately concentrated in groups with higher SDI, while a negative SII value suggests concentration in groups with lower SDI. The CI is a relative measure of health inequality. It is calculated by fitting a Lorenz concentration curve based on cumulative DALYs or deaths and cumulative population proportions. The CI is obtained through numerical integration of the area under the curve, with values ranging from -1 to 1. Values closer to 0 indicate lower levels of inequality. Positive values suggest better health outcomes among wealthier groups while negative values indicate better health outcomes among poorer groups.

- 1. **Decomposition Analysis**

Method: Specify the use of the “Das Gupta's method for demographic decomposition”.Factors: List the three factors decomposed: “Population Growth", "Population Aging (age structure change)”, and “Epidemiological Change (age-specific rate change)”.We calculated the expected changes caused by population growth, the additional changes driven by population aging, and attributed the residual changes to epidemiological factors.

- 1. **Age-Period-Cohort Analysis**

Specify the “Intrinsic Estimator (IE) APC model” to address the identification problem. The model was fitted using the Epi package (version 2.46) in R, with parameter estimation based on 5-year age groups (0-4 years to 95-100 years) and 5-year periods (1992-1996 to 2017-2021). Mortality rates were calculated using the global population as the reference cohort. The integrated model data underwent final analysis on the platform available at <https://analysistools.cancer.gov/apc/>. Model selection was determined by residual deviance and Akaike Information Criterion (AIC) values.

- 1. **Forecasting Model (ARIMA)**

The ARIMA model consists of an autoregressive (AR) component and a moving average (MA) component. Its fundamental assumption is that the data sequence represents time-dependent random variables, whose autocorrelation can be characterized by the ARIMA model, and future values can be predicted based on historical values. Using data from 1990 to 2021, this model forecasts trends for the years 2022 to 2036. The model formula is as follows:

$$\boldsymbol{Y}_{\boldsymbol{t}}\boldsymbol{=}\sum_{\boldsymbol{i=1}}^{\boldsymbol{p}} \boldsymbol{\emptyset}_{\boldsymbol{i}}\boldsymbol{Y}_{\boldsymbol{t-i}}\boldsymbol{+}\boldsymbol{e}_{\boldsymbol{t}}\boldsymbol{-}\sum_{\boldsymbol{j=1}}^{\boldsymbol{q}} \boldsymbol{\theta}_{\boldsymbol{j}}\boldsymbol{e}_{\boldsymbol{t-j}}$$

Here, p (the order of autoregression) and q (the order of moving average) are optimized using the AIC via the auto.arima() function from the forecast package (version 8.24.0) in R. The final models selected for this study were: HIV/AIDS and STIs (ASMR): ARIMA(0,2,0); Other Infectious Diseases (ASMR): ARIMA(1,1,0). The selected model was subjected to residual diagnostics to ensure no significant autocorrelation (assessed via the Ljung-Box test, P-value＞0.05) and approximate normality. The final predictions include median estimates and 95% prediction intervals, which account for the historical volatility in the data. Model stability was assessed via out-of-sample validation: fitting the model to 1990–2010 data and forecasting 2011–2021. The predicted trend closely aligned with observed data, supporting the model's robustness for medium-term projection.
