# Supplementary material for: Global burden and forecast of infectious diseases attributable to drug use: evidence from GBD 2021
Source: Front Public Health. 2025 Dec 15;13:1706764. doi: 10.3389/fpubh.2025.1706764 (PMC12745377; doi:10.3389/fpubh.2025.1706764)
Supplement: Supplementary file 2 [file Data_Sheet_2.DOCX]

**The following reproducible R code was used for all analyses in this study. There are 16 parts (Part 1–16).**

**PART 1 Trend**

setwd("D:/ ")

library(dplyr)

library(ggplot2)

RHD <- read.csv('total.csv',header = T)

RHD <- RHD[,c(3,5,7,9,11,13,14,15,16,17)]

RHD_RATE_times <- subset(RHD,

RHD$age_name=='Age-standardized' &

RHD$metric_name== 'Rate'&

RHD$sex_name=='Both')

RHD_RATE_times <- RHD_RATE_times[,c(1,2,7,8)]

####China

RHD_RATE_times_china <-subset(RHD_RATE_times,

RHD_RATE_times$location_name=='China'

)

names(RHD_RATE_times_china)[names(RHD_RATE_times_china) == 'measure_name'] <- 'measure'

RHD_RATE_times_china$measure[which(RHD_RATE_times_china$measure=='Deaths')] <- 'ASMR'

RHD_RATE_times_china$measure[which(RHD_RATE_times_china$measure=='Incidence')] <- 'ASIR'

RHD_RATE_times_china$measure[which(RHD_RATE_times_china$measure=='Prevalence')] <- 'ASPR'

RHD_RATE_times_china$measure[which(RHD_RATE_times_china$measure=='DALYs (Disability-Adjusted Life Years)')] <- 'DALYs Rate'

p1=ggplot(RHD_RATE_times_china,aes(x =year,y =val, group=measure,color = measure, fill = measure))+

geom_line()+

geom_point()+

theme_bw()+

theme(legend.text=element_text(size=14))+

theme(title=element_text(size=9))+

theme(axis.text.x = element_text(size = 14, color = "black"))+

theme(axis.text.y = element_text(size = 14, color = "black"))+

ylab("Age-standardized Rate (per 100,000)")+

theme(text=element_text(size=14, family="serif"))+##Times New Roman)

labs(title= "1990-2019 China Age-standardized Rate")

p1

####Global1------------------------------------------------

RHD_RATE_times <- read.csv('Global_infections1.csv',header = T)

RHD_RATE_times_Global <- subset(RHD_RATE_times,

RHD_RATE_times$age_name=='Age-standardized' &

RHD_RATE_times$metric_name== 'Rate'&

RHD_RATE_times$sex_name=='Both')

RHD_RATE_times_Global <- RHD_RATE_times_Global[,c(5,7,9,11,13,17,18,19,20,21)]

RHD_RATE_times_Global <- RHD_RATE_times_Global[,c(1,2,7,8)]

names(RHD_RATE_times_Global)[names(RHD_RATE_times_Global) == 'measure_name'] <- 'measure'

RHD_RATE_times_Global$measure[which(RHD_RATE_times_Global$measure=='Deaths')] <- 'ASMR'

RHD_RATE_times_Global$measure[which(RHD_RATE_times_Global$measure=='DALYs (Disability-Adjusted Life Years)')] <- 'DALYs Rate'

p2=ggplot(RHD_RATE_times_Global,aes(x =year,y =val, group=measure,color = measure, fill = measure))+

geom_line()+

geom_point()+

scale_color_manual(values=c("#807DBA","#FC9272"))+

theme_bw()+

theme(legend.text=element_text(size=14))+

theme(title=element_text(size=9))+

theme(axis.text.x = element_text(size = 14, color = "black"))+

theme(axis.text.y = element_text(size = 14, color = "black"))+

ylab("Age-standardized Rate (per 100,000)")+

theme(text=element_text(size=14, family="serif"))+

#theme(legend.position ='top')+

labs(title= "1990-2021 Global Age-standardized Rate")

p2

####Global2------------------------------------------------

RHD_RATE_times <- read.csv('Global_infections2.csv',header = T)

RHD_RATE_times_Global <- subset(RHD_RATE_times,

RHD_RATE_times$age_name=='Age-standardized' &

RHD_RATE_times$metric_name== 'Rate'&

RHD_RATE_times$sex_name=='Both')

RHD_RATE_times_Global <- RHD_RATE_times_Global[,c(5,7,9,11,13,17,18,19,20,21)]

RHD_RATE_times_Global <- RHD_RATE_times_Global[,c(1,2,7,8)]

names(RHD_RATE_times_Global)[names(RHD_RATE_times_Global) == 'measure_name'] <- 'measure'

RHD_RATE_times_Global$measure[which(RHD_RATE_times_Global$measure=='Deaths')] <- 'ASMR'

RHD_RATE_times_Global$measure[which(RHD_RATE_times_Global$measure=='DALYs (Disability-Adjusted Life Years)')] <- 'DALYs Rate'

p3=ggplot(RHD_RATE_times_Global,aes(x =year,y =val, group=measure,color = measure, fill = measure))+

geom_line()+

geom_point()+

scale_color_manual(values=c("#807DBA","#FC9272"))+

theme_bw()+

theme(legend.text=element_text(size=14))+

theme(title=element_text(size=9))+

theme(axis.text.x = element_text(size = 14, color = "black"))+

theme(axis.text.y = element_text(size = 14, color = "black"))+

ylab("Age-standardized Rate (per 100,000)")+

theme(text=element_text(size=14, family="serif"))+

#theme(legend.position ='top')+

labs(title= "1990-2021 Global Age-standardized Rate")

p3

library(ggpubr)

ggarrange(p2,p3,nrow=1,ncol=2,labels="AUTO")

**PART 2 Table 1**

#### Table1 2021年不同性别全年龄及年龄标化率的疾病负担####

#加载数据整理包

library(dplyr)

library(ggplot2)

####读取数据####

setwd("D:/ Table1") #设立指定的目录

IBD_super_region <- read.csv('IBD_super_region.csv',header = T) #事先提取数据

#查看数据的格式

str(IBD_china)

table(IBD_china$measure_name)

table(IBD_china$metric_name)

unique(IBD_super_region$cause_name)

#提取全球传染性疾病数据

IBD_Global<-subset(IBD_super_region, ####subset()函数类似excel的筛选功能，用来选数据的子集，也可在Excel完成

IBD_super_region$location_name =='Global' ###全球

)

IBD_Global_infections<-subset(IBD_Global,

IBD_Global$cause_name =='HIV/AIDS and sexually transmitted infections'|

IBD_Global$cause_name =='Other infectious diseases' ###选择cause

)

####第一步：首先提取2021年全年龄段(All ages)的人数(Number)####

IBD_Global_infections_number1<-subset(IBD_Global_infections, ####subset()函数类似excel的筛选功能，用来选数据的子集，也可在Excel完成

IBD_Global_infections$age_name=='All ages' & ###选择年龄为全年龄段

IBD_Global_infections$year =='1990'& ###选择年份为2021

IBD_Global_infections$metric_name== 'Number' ###选择数据格式为数值

)

IBD_Global_infections_number2<-subset(IBD_Global_infections, ####subset()函数类似excel的筛选功能，用来选数据的子集，也可在Excel完成

IBD_Global_infections$age_name=='All ages' & ###选择年龄为全年龄段

IBD_Global_infections$year =='2021'& ###选择年份为2021

IBD_Global_infections$metric_name== 'Number' ###选择数据格式为数值

)

IBD_Global_infections_number<-rbind(IBD_Global_infections_number1,IBD_Global_infections_number2)

#####由于是人数，所以我们取整数

IBD_Global_infections_number$val <- round(IBD_Global_infections_number$val,0)

IBD_Global_infections_number$lower <- round(IBD_Global_infections_number$lower,0)

IBD_Global_infections_number$upper <- round(IBD_Global_infections_number$upper,0)

####把数值与其上下不确定性区间合并为一个单元格

IBD_Global_infections_number$UI <- paste(IBD_Global_infections_number$lower,IBD_Global_infections_number$upper,sep = ',')

IBD_Global_infections_number$UI <- paste(IBD_Global_infections_number$UI,')',sep = '') ##95%UI

IBD_Global_infections_number$UI <- paste('(',IBD_Global_infections_number$UI,sep = '') ##95%UI

IBD_Global_infections_number$UI <- paste(IBD_Global_infections_number$val,IBD_Global_infections_number$UI,sep = ' ')

write.csv(IBD_Global_infections_number,"Global_infections_number.csv")

unique(IBD_Global_infections$cause_name)

####第二步，我们提取年龄标准化(Age-standardized)的率(Rate)####

IBD_Global_infections_Rate1<-subset(IBD_Global_infections, ####subset()函数类似excel的筛选功能，用来选数据的子集，也可在Excel完成

IBD_Global_infections$age_name=='Age-standardized' & ###选择年龄为年龄标准化

IBD_Global_infections$year =='1990'& ###选择年份

IBD_Global_infections$metric_name== 'Rate' ###选择数据格式为率

)

IBD_Global_infections_Rate2<-subset(IBD_Global_infections, ####subset()函数类似excel的筛选功能，用来选数据的子集，也可在Excel完成

IBD_Global_infections$age_name=='Age-standardized' & ###选择年龄为年龄标准化

IBD_Global_infections$year =='2021'& ###选择年份为2021

IBD_Global_infections$metric_name== 'Rate' ###选择数据格式为率

)

IBD_Global_infections_Rate<-rbind(IBD_Global_infections_Rate1,IBD_Global_infections_Rate2)

#####由于是率，所以我们取2位或3为小数，这里我们取2位小数

IBD_Global_infections_Rate$val <- round(IBD_Global_infections_Rate$val,2)

IBD_Global_infections_Rate$lower <- round(IBD_Global_infections_Rate$lower,2)

IBD_Global_infections_Rate$upper <- round(IBD_Global_infections_Rate$upper,2)

####把数值与其上下不确定性区间合并为一个单元格

IBD_Global_infections_Rate$UI <- paste(IBD_Global_infections_Rate$lower,IBD_Global_infections_Rate$upper,sep = ',')

IBD_Global_infections_Rate$UI <- paste(IBD_Global_infections_Rate$UI,')',sep = '') ##95%UI

IBD_Global_infections_Rate$UI <- paste('(',IBD_Global_infections_Rate$UI,sep = '') ##95%UI

IBD_Global_infections_Rate$UI <- paste(IBD_Global_infections_Rate$val,IBD_Global_infections_Rate$UI,sep = ' ')

####第三步，结果输出####

write.csv(IBD_Global_infections_number,"Global_infections_number.csv") ###输出结果为csv文件

write.csv(IBD_Global_infections_Rate,"Global_infections_Rate.csv") ###输出结果为csv文件

#percentage_change

Total_percentage_change <- read.csv('IHME-GBD_2021_DATA-113c99aa-1.csv',header = T)

#提取全球传染性疾病数据

percentage_change_Global<-subset(Total_percentage_change, ####subset()函数类似excel的筛选功能，用来选数据的子集，也可在Excel完成

Total_percentage_change$location_name =='Global' ###全球

)

percentage_change_infections<-subset(percentage_change_Global,

percentage_change_Global$cause_name =='HIV/AIDS and sexually transmitted infections'|

percentage_change_Global$cause_name =='Other infectious diseases' ###选择cause

)

#unique(percentage_change_infections$location_name)

####第一步：首先提取全年龄段(All ages)的(Rate)####

#unique(percentage_change_infections$age_name)

percentage_change_All_ages<-subset(percentage_change_infections, ####subset()函数类似excel的筛选功能，用来选数据的子集，也可在Excel完成

percentage_change_infections$age_name=='All ages' & ###选择年龄为全年龄段

percentage_change_infections$metric_name== 'Rate' ###选择数据格式为数值

)

#####由于是率，所以我们取2位或3为小数，这里我们取2位小数

percentage_change_All_ages$val <- round(percentage_change_All_ages$val,2)

percentage_change_All_ages$lower <- round(percentage_change_All_ages$lower,2)

percentage_change_All_ages$upper <- round(percentage_change_All_ages$upper,2)

####把数值与其上下不确定性区间合并为一个单元格

percentage_change_All_ages$UI <- paste(percentage_change_All_ages$lower,percentage_change_All_ages$upper,sep = ',')

percentage_change_All_ages$UI <- paste(percentage_change_All_ages$UI,')',sep = '') ##95%UI

percentage_change_All_ages$UI <- paste('(',percentage_change_All_ages$UI,sep = '') ##95%UI

percentage_change_All_ages$UI <- paste(percentage_change_All_ages$val,percentage_change_All_ages$UI,sep = ' ')

write.csv(percentage_change_All_ages,"percentage_change_All_ages.csv")

####第二步，我们提取年龄标准化(Age-standardized)的率(Rate)####

percentage_change_Age_standardized<-subset(percentage_change_infections, ####subset()函数类似excel的筛选功能，用来选数据的子集，也可在Excel完成

percentage_change_infections$age_name=='Age-standardized' & ###选择年龄为全年龄段

percentage_change_infections$metric_name== 'Rate' ###选择数据格式为数值

)

#####由于是率，所以我们取2位或3为小数，这里我们取2位小数

percentage_change_Age_standardized$val <- round(percentage_change_Age_standardized$val,2)

percentage_change_Age_standardized$lower <- round(percentage_change_Age_standardized$lower,2)

percentage_change_Age_standardized$upper <- round(percentage_change_Age_standardized$upper,2)

####把数值与其上下不确定性区间合并为一个单元格

percentage_change_Age_standardized$UI <- paste(percentage_change_Age_standardized$lower,percentage_change_Age_standardized$upper,sep = ',')

percentage_change_Age_standardized$UI <- paste(percentage_change_Age_standardized$UI,')',sep = '') ##95%UI

percentage_change_Age_standardized$UI <- paste('(',percentage_change_Age_standardized$UI,sep = '') ##95%UI

percentage_change_Age_standardized$UI <- paste(percentage_change_Age_standardized$val,percentage_change_Age_standardized$UI,sep = ' ')

####第三步，结果输出####

###输出结果为csv文件

write.csv(percentage_change_Age_standardized,"percentage_change_Age_standardized.csv") ###输出结果为csv文件

**PART 3 Age-Period-Cohort model**

# Install APC software

library(Matrix)

apc2fit <- function(R, ...)

{

# Fit age-period-cohort model that includes quadratic terms.

#

# Args:

# OverDispersion: 1/TRUE (default) or 0/FALSE

# RVals: 3-element vector of age, period, and cohort referent values;

# defaults are mid-point values

# offset_tick: standard offset unit, default is 10^5

# zero_fill: scalar, value to replace 0 counts, default is 0.1

# HiC: include higher-order cohort deviations, 1/TRUE (default) or 0/FALSE

# HiP: include higher-order period deviations, 1/TRUE (default) or 0/FALSE

# HiA: include higher-order age deviations, 1/TRUE (default) or 0/FALSE

#

# Returns:

# Age-Period-Cohort model outputs

PVP <- checkPVPPAIRS(R, ...)

A <- length(PVP$D$a)

P <- length(PVP$D$p)

C <- length(PVP$D$c)

if (A<3 || P<3) {stop("This functions requires three or more age groups and calendar periods.")}

if ( (A==3 || P==3) && (PVP$HiA == FALSE || PVP$HiP == FALSE || PVP$HiC == FALSE) ) {

stop("You must accept default HiA, HiP, and HiC values when there are only three age groups or calendar periods.")}

if (A==3) {A3NULL <- cbind(matrix(PVP$D$a), matrix(0, nrow=A), matrix(-1E-6, nrow=A), matrix(+1E-6, nrow=A))}

if (P==3) {P3NULL <- cbind(matrix(PVP$D$p), matrix(0, nrow=P), matrix(-1E-6, nrow=P), matrix(+1E-6, nrow=P))}

D <- designmatrix(PVP)

Pt <- D$Pt

MOD <- 1 + PVP$HiA*4 + PVP$HiP*2 + PVP$HiC*1

if (MOD == 1) {

# No HiA, No HiP, No HiC

apcM <- APCFIT(PVP, D$X[,1:6])

mapc <- length(Pt[[7]]) + length(Pt[[8]]) + length(Pt[[9]])

apcM$B <- rbind(apcM$B, matrix(0, nrow = mapc))

apcM$s2VAR <- as.matrix(bdiag(list(apcM$s2VAR, matrix(1E-6, nrow = mapc, ncol= mapc))))

} else if (MOD == 2) {

# No HiA, No HiP, With HiC

apcM <- APCFIT(PVP, D$X[,c(1:6, Pt[[9]])])

ma <- length(Pt[[7]])

mp <- length(Pt[[8]])

apcM$B <- rbind(as.matrix(apcM$B[1:6]),

matrix(0, nrow = ma + mp),

as.matrix(apcM$B[Pt[[9]] - ma - mp]))

apcM$s2VAR <- as.matrix(bdiag(list(apcM$s2VAR[1:6,1:6],

matrix(1E-6, nrow = ma + mp, ncol = ma + mp),

apcM$s2VAR[Pt[[9]] - ma - mp, Pt[[9]] - ma - mp])))

}

else if (MOD == 3) {

# No HiA, With HiP, No HiC

apcM <- APCFIT(PVP, D$X[,c(1:6, Pt[[8]])])

ma <- length(Pt[[7]])

mc <- length(Pt[[9]])

apcM$B <- rbind(as.matrix(apcM$B[1:6]),

matrix(0, nrow = ma),

as.matrix(apcM$B[Pt[[8]] - ma]),

matrix(0, nrow = mc))

apcM$s2VAR <- as.matrix(bdiag(list(apcM$s2VAR[1:6,1:6],

matrix(1E-6, nrow = ma, ncol = ma),

apcM$s2VAR[Pt[[8]] - ma, Pt[[8]] - ma],

matrix(1E-6, nrow = mc, ncol = mc))))

}

else if (MOD == 4) {

# No HiA, With HiP, With HiC

apcM <- APCFIT(PVP, D$X[,c(1:6, Pt[[8]], Pt[[9]])])

ma <- length(Pt[[7]])

apcM$B <- rbind(as.matrix(apcM$B[1:6]),

matrix(0, nrow = ma),

as.matrix(apcM$B[c(Pt[[8]], Pt[[9]]) - ma]))

apcM$s2VAR <- as.matrix(bdiag(list(apcM$s2VAR[1:6,1:6],

matrix(1E-6, nrow = ma, ncol = ma),

apcM$s2VAR[c(Pt[[8]], Pt[[9]]) - ma, c(Pt[[8]], Pt[[9]]) - ma])))

}

else if (MOD == 5) {

# With HiA, No HiP, No HiC

apcM <- APCFIT(PVP, D$X[,c(1:6, Pt[[7]])])

mpc <- length(Pt[[8]]) + length(Pt[[9]])

apcM$B <- rbind(as.matrix(apcM$B),

matrix(0, nrow = mpc))

apcM$s2VAR <- as.matrix(bdiag(list(apcM$s2VAR,

matrix(1E-6, nrow = mpc, ncol = mpc))))

}

else if (MOD == 6) {

# With HiA, No HiP, With HiC

INC0 <- c(1:6, Pt[[7]])

INC1 <- Pt[[9]]

apcM <- APCFIT(PVP, D$X[,c(INC0, INC1)])

mp <- length(Pt[[8]])

apcM$B <- rbind(as.matrix(apcM$B[INC0]),

matrix(0, nrow = mp),

as.matrix(apcM$B[INC1 - mp]))

apcM$s2VAR <- as.matrix(bdiag(list(apcM$s2VAR[INC0,INC0],

matrix(0, nrow = mp, ncol = mp),

apcM$s2VAR[ INC1 - mp, INC1 - mp ])))

}

else if (MOD == 7) {

# With HiA, With HiP, No HiC

apcM <- APCFIT(PVP, D$X[,c(1:6, Pt[[7]], Pt[[8]])])

mc <- length(Pt[[9]])

INC <- c(1:6, Pt[[7]], Pt[[8]])

apcM$B <- rbind(apcM$B, matrix(0, nrow = mc))

apcM$s2VAR <- as.matrix(bdiag(list(apcM$s2VAR, matrix(1E-6, nrow = mc, ncol = mc))))

}

else if (MOD == 8) {

# With HiA, With HiP, With HiC

apcM <- APCFIT(PVP, D$X)

}

else

{ stop("Invalid value or values for HiA, HiP, or HiC.") }

B <- apcM$B

s2VAR <-apcM$s2VAR

#

# (0) Fitted rates

#

ETA <- D$X%*%B

# Formula to compute only the variances of fitted rates, not any covariances.

v <- rowSums((D$X%*%s2VAR)*D$X)

# Scale the values such that naive formulae yield correct variances.

v[v<0] <- NaN

EFit <- matrix(1/v, nrow=A, ncol=P)

OFit <- matrix((1/v)*exp(-ETA), nrow=A, ncol=P)

FittedRates <- list(name = paste('Fitted', PVP$name),

events = EFit,

offset = OFit,

offset_tick = PVP$offset_tick,

ages = R$ages,

periods = R$periods);

#

# (1) Coefficents

# Intercept, LAT, NetDrift, CAT, THETAa, THETAp, THETAc

#

XCO <- matrix(c(1, 0, 0, 0, 0, 0,

0, 1, 0, 0, 0, 0,

0, 0, 1, 0, 0, 0,

0, 1, -1, 0, 0, 0,

0, 0, 0, 1, 0, 0,

0, 0, 0, 0, 1, 0,

0, 0, 0, 0, 0, 1),

nrow = 7, byrow = T)

b7 <- XCO%*%B[1:6]

v7 <- XCO%*%s2VAR[1:6,1:6]%*%t(XCO)

s7 <- matrix(sqrt(diag(v7)))

c7 <- cbind(b7 - 1.96*s7, b7 + 1.96*s7)

Coefficients <- cbind(b7, s7, c7)

dimnames(Coefficients) <- list(c("Intercept","LAT","NetDrift", "CAT", "THETAa", "THETAp", "THETAc"), c("Parameter","SD","CILo", "CIHi"))

#

# Wald test - NetDrift different from 0?

#

X21 <- (b7[3]/s7[3])^2

df1 <- 1

PVAL1 <- pchisq(X21, df1,lower.tail = FALSE)

#

# Net Drift - as estimated annual percentage change (EAPC)

#

b3 <- b7[3];

v3 <- v7[3, 3]

s3 <- sqrt(v3)

c3 <- cbind(b3 - 1.96*s3, b3 + 1.96*s3)

NetDrift <- cbind(b3, c3)

NetDrift <- 100*(exp(NetDrift) - 1)

dimnames(NetDrift) <- list( c(), c("Net Drift (%/year)", "CILo", "CIHi") )

#

# Global Curvature

#

bq <- b7[5:7]

sq <- matrix(sqrt(diag(v7[5:7,5:7])))

cq <- cbind(bq - 1.96*sq, bq + 1.96*sq)

eq <- 100*(exp(bq)-1)

ecq <- 100*(exp(cq)-1)

GlobalCurvature <- cbind(eq, ecq)

dimnames(GlobalCurvature) <- list(c("Age", "Period", "Cohort"), c("%/yr^2", "CILo", "CIHi"))

#

# (2a) Quadratic Age Deviations

#

astar <- matrix(PVP$D$a)

abar <- mean(astar)

aref <- PVP$RVals[1]

arefLOC <- match(aref, astar)

XAQ <- matrix(D$X[D$INCa, 4])

ad2 <- XAQ%*%B[4]

ad2v <- XAQ%*%s2VAR[4,4]%*%t(XAQ)

sd <- matrix(sqrt(diag(ad2v)))

ci <- cbind(ad2 - 1.96*sd, ad2 + 1.96*sd)

QuadAgeDeviations <- cbind(astar, ad2, ci)

dimnames(QuadAgeDeviations) <- list(c(), c("Age", "Deviation", "CILo", "CIHi"))

# Wald test - Quadratic Age Effect different from 0?

X22 <- (b7[5]/s7[5])^2

df2 <- 1

PVAL2 <- pchisq(X22, df2, lower.tail = FALSE)

#

# (2b) Higher-Order Age Deviations

#

XAD <- matrix(D$X[D$INCa, Pt[[7]]], nrow = A, byrow = F)

adh <- XAD%*%B[Pt[[7]]]

adhv <- XAD%*%s2VAR[Pt[[7]],Pt[[7]]]%*%t(XAD)

sd <- matrix(sqrt(diag(adhv)))

ci <- cbind(adh - 1.96*sd, adh + 1.96*sd)

HiOrdAgeDeviations <- cbind(astar, adh, ci)

if (A==3) {HiOrdAgeDeviations <- A3NULL}

dimnames(HiOrdAgeDeviations) <- list(c(), c("Age", "Deviation", "CILo", "CIHi"))

# Wald test - any Higher-Order age deviations different from 0?

if (PVP$HiA==1 && A>3){

X23 <- t(matrix(adh[2:(A-2)]))%*%solve(adhv[2:(A-2),2:(A-2)],matrix(adh[2:(A-2)]))

df3 <- A - 3

PVAL3 <- pchisq(X23, df3, lower.tail = FALSE)

}

else{

X23 <- NaN

df3 <- NaN

PVAL3 <- 1

}

#

# (2c) Age Deviations, Complete

#

XA <- cbind(XAQ, XAD)

ad <- XA%*%B[c(4, Pt[[7]])]

adv <- XA%*%s2VAR[c(4, Pt[[7]]),c(4, Pt[[7]])]%*%t(XA)

sd <- matrix(sqrt(diag(adv)))

ci <- cbind(ad - 1.96*sd, ad + 1.96*sd)

AgeDeviations <- cbind(astar, ad, ci)

dimnames(AgeDeviations) <- list(c(), c("Age", "Deviation", "CILo", "CIHi"))

if (A==3) {AgeDeviations <- QuadAgeDeviations; adv <- ad2v}

# Wald test - any complete age deviations different from 0?

if (PVP$HiA==1 && A>3){

X24 <- t(matrix(ad[2:(A-1)]))%*%solve(adv[2:(A-1),2:(A-1)],matrix(ad[2:(A-1)]))

df4 <- A - 2

PVAL4 <- pchisq(X24, df4, lower.tail = FALSE)

}

else{

X24 <- (b7[5]/s7[5])^2

df4 <- 1

PVAL4 <- pchisq(X24, df4, lower.tail = FALSE)

}

#

# (3a) Quadratic Period Deviations

#

pstar <- matrix(PVP$D$p)

pbar <- mean(pstar)

pref <- PVP$RVals[2]

prefLOC <- match(pref, pstar)

XPQ <- matrix(D$X[D$INCp, 5])

pd2 <- XPQ%*%B[5]

pd2v <- XPQ%*%s2VAR[5,5]%*%t(XPQ)

sd <- matrix(sqrt(diag(pd2v)))

ci <- cbind(pd2 - 1.96*sd, pd2 + 1.96*sd)

QuadPerDeviations <- cbind(pstar, pd2, ci)

dimnames(QuadPerDeviations) <- list(c(), c("Period", "Deviation", "CILo", "CIHi"))

# Wald test - Quadratic Period Effect different from 0?

X25 <- (b7[6]/s7[6])^2

df5 <- 1

PVAL5 <- pchisq(X25, df5,lower.tail = FALSE)

#

# (3b) Higher-Order Period Deviations

#

XPD <- matrix(D$X[D$INCp, Pt[[8]]], nrow = P, byrow = F)

pdh <- XPD%*%B[Pt[[8]]]

pdhv <- XPD%*%s2VAR[Pt[[8]],Pt[[8]]]%*%t(XPD)

sd <- matrix(sqrt(diag(pdhv)))

ci <- cbind(pdh - 1.96*sd, pdh + 1.96*sd)

HiOrdPerDeviations <- cbind(pstar, pdh, ci)

if (P==3) {HiOrdPerDeviations <- P3NULL}

dimnames(HiOrdPerDeviations) <- list(c(), c("Period", "Deviation", "CILo", "CIHi"))

# Wald test - any Higher-Order per deviations different from 0?

if (PVP$HiP==1 && P>3){

X26 <- t(matrix(pdh[2:(P-2)]))%*%solve(pdhv[2:(P-2),2:(P-2)],matrix(pdh[2:(P-2)]))

df6 <- P - 3

PVAL6 <- pchisq(X26, df6, lower.tail = FALSE)

}

else {

X26 <- NaN

df6 <- NaN

PVAL6 <- 1

}

#

# (3c) Period Deviations, Complete

#

XP <- cbind(XPQ, XPD)

pd <- XP%*%B[c(5, Pt[[8]])]

pdv <- XP%*%s2VAR[c(5, Pt[[8]]),c(5, Pt[[8]])]%*%t(XP)

sd <- matrix(sqrt(diag(pdv)))

ci <- cbind(pd - 1.96*sd, pd + 1.96*sd)

PerDeviations <- cbind(pstar, pd, ci)

if (P==3) {PerDeviations <- QuadPerDeviations; pdv <- pd2v}

dimnames(PerDeviations) <- list(c(), c("Period", "Deviation", "CILo", "CIHi"))

# Wald test - any complete period deviations different from 0?

if (PVP$HiP==1 && P>3){

X27 <- t(matrix(pd[2:(P-1)]))%*%solve(pdv[2:(P-1),2:(P-1)],matrix(pd[2:(P-1)]))

df7 <- P - 2

PVAL7 <- pchisq(X27, df7, lower.tail = FALSE)

}

else{

X27 <- X25

df7 <- df5

PVAL7 <- PVAL5

}

#

# (4a) Quadratic Cohort Deviations

#

cstar <- matrix(PVP$D$c)

cbar <- mean(cstar)

cref <- PVP$RVals[3]

crefLOC <- match(cref, cstar)

XCQ <- matrix(D$X[D$INCc, 6])

cd2 <- XCQ%*%B[6]

cd2v <- XCQ%*%s2VAR[6,6]%*%t(XCQ)

sd <- matrix(sqrt(diag(cd2v)))

ci <- cbind(cd2 - 1.96*sd, cd2 + 1.96*sd)

QuadCohDeviations <- cbind(cstar, cd2, ci)

dimnames(QuadCohDeviations) <- list(c(), c("Cohort", "Deviation", "CILo", "CIHi"))

# Wald test - Quadratic Cohort Effect different from 0?

X28 <- (b7[7]/s7[7])^2

df8 <- 1

PVAL8 <- pchisq(X28, df8, lower.tail = FALSE)

#

# (4b) Higher-Order Cohort Deviations

#

XCD <- matrix(D$X[D$INCc, Pt[[9]]], nrow = C, byrow = F)

cdh <- XCD%*%B[Pt[[9]]]

cdhv <- XCD%*%s2VAR[Pt[[9]],Pt[[9]]]%*%t(XCD)

sd <- matrix(sqrt(diag(cdhv)))

ci <- cbind(cdh - 1.96*sd, cdh + 1.96*sd)

HiOrdCohDeviations <- cbind(cstar, cdh, ci)

dimnames(HiOrdCohDeviations) <- list(c(), c("Cohort", "Deviation", "CILo", "CIHi"))

# Wald test - any Higher-Order coh deviations different from 0?

if (PVP$HiC==1){

X29 <- t(matrix(cdh[3:(C-1)]))%*%solve(cdhv[3:(C-1),3:(C-1)],matrix(cdh[3:(C-1)]))

df9 <- C - 3

PVAL9 <- pchisq(X29, df9, lower.tail = FALSE)

}

else{

X29 <- NaN

df9 <- NaN

PVAL9 <- 1

}

#

# (4c) Cohort Deviations, Complete

#

XC <- cbind(XCQ, XCD)

cd <- XC%*%B[c(6, Pt[[9]])]

cdv <- XC%*%s2VAR[c(6, Pt[[9]]),c(6, Pt[[9]])]%*%t(XC)

sd <- matrix(sqrt(diag(cdv)))

ci <- cbind(cd - 1.96*sd, cd + 1.96*sd)

CohDeviations <- cbind(cstar, cd, ci)

dimnames(CohDeviations) <- list(c(), c("Cohort", "Deviation", "CILo", "CIHi"))

# Wald test - any complete coh deviations different from 0?

if (PVP$HiC==1){

X210 <- t(matrix(cd[2:(C-1)]))%*%solve(cdv[2:(C-1),2:(C-1)],matrix(cd[2:(C-1)]))

df10 <- C - 2

PVAL10 <- pchisq(X210, df10, lower.tail = FALSE)

}

else{

X210 <- X28

df10 <- df8

PVAL10 <- PVAL8

}

#

# (5a) Quadratic Longitudinal Age Curve - offset by complete cohort

# deviation, plus net drift times offset of reference value of cohort

# versus mean value of cohort

#

lot <- log(PVP$offset_tick)

XLAQ <- cbind(matrix(1, A), astar-abar, matrix(1, A)%*%(cref - cbar), XAQ, matrix(1, A)%*%XC[crefLOC,])

lac2 <- lot + XLAQ%*%B[c(1, 2, 3, 4, 6, Pt[[9]])]

lac2v <- XLAQ%*%s2VAR[c(1, 2, 3, 4, 6, Pt[[9]]), c(1, 2, 3, 4, 6, Pt[[9]])]%*%t(XLAQ)

sd <- matrix(sqrt(diag(lac2v)))

ci <- cbind(lac2 - 1.96*sd, lac2 + 1.96*sd)

QuadLongAge <- cbind(astar, exp(lac2), exp(ci))

dimnames(QuadLongAge) <- list(c(), c("Age", "Rate", "CILo", "CIHi"))

#

# (5b) Quadratic Longitudinal Age Rate Ratios

#

TMP <- diag(0, nrow = A)

TMP[,arefLOC] <- 1

ARR <- diag(1, nrow = A) - TMP

LARQ <- ARR%*%XLAQ

lac2rr <- LARQ%*%B[c(1, 2, 3, 4, 6, Pt[[9]])]

lac2rrv <- LARQ%*%s2VAR[c(1, 2, 3, 4, 6, Pt[[9]]), c(1, 2, 3, 4, 6, Pt[[9]])]%*%t(LARQ)

sd <- matrix(sqrt(diag(lac2rrv)))

ci <- cbind(lac2rr - 1.96*sd, lac2rr + 1.96*sd)

QuadLongAgeRR <- cbind(astar, exp(lac2rr), exp(ci))

dimnames(QuadLongAgeRR) <- list(c(), c("Age", "Rate Ratio", "CILo", "CIHi"))

#

# (5c) Complete Longitudinal Age Curve

#

XLA <- cbind(matrix(1, A), astar-abar, matrix(1, A)%*%(cref - cbar), XAQ, XAD, matrix(1, A)%*%XC[crefLOC,])

lac <- lot + XLA%*%B[c(1, 2, 3, 4, Pt[[7]], 6, Pt[[9]])]

lacv <- XLA%*%s2VAR[c(1, 2, 3, 4, Pt[[7]], 6, Pt[[9]]), c(1, 2, 3, 4, Pt[[7]], 6, Pt[[9]])]%*%t(XLA)

sd <- matrix(sqrt(diag(lacv)))

ci <- cbind(lac - 1.96*sd, lac + 1.96*sd)

LongAge <- cbind(astar, exp(lac), exp(ci))

if (A==3) {LongAge <- QuadLongAge; lacv <- lac2v}

dimnames(LongAge) <- list(c(), c("Age", "Rate", "CILo", "CIHi"))

#

# (5d) Longitudinal Age Rate Ratios

#

LAR <- ARR%*%XLA

lacrr <- LAR%*%B[c(1, 2, 3, 4, Pt[[7]], 6, Pt[[9]])]

lacrrv <- LAR%*%s2VAR[c(1, 2, 3, 4, Pt[[7]], 6, Pt[[9]]), c(1, 2, 3, 4, Pt[[7]], 6, Pt[[9]])]%*%t(LAR)

sd <- matrix(sqrt(diag(lacrrv)))

ci <- cbind(lacrr - 1.96*sd, lacrr + 1.96*sd)

LongAgeRR <- cbind(astar, exp(lacrr), exp(ci))

if (A==3) {LongAgeRR <- QuadLongAgeRR; lacrrv <- lac2rrv}

dimnames(LongAgeRR) <- list(c(), c("Age", "Rate Ratio", "CILo", "CIHi"))

#

# (6a) Quadratic Cross-Sectional Age Curve - offset by complete period

# deviation plus net drift times offset of reference value of period versus

# mean value of period

#

if (P>3){

XXAQ <- cbind(matrix(1, A), astar-abar, matrix(1, A)%*%(pref - pbar) - (astar-abar), XAQ, matrix(1, A)%*%XP[prefLOC,])

BINC2 <- c(1, 2, 3, 4, 5, Pt[[8]])}

else{

XXAQ <- cbind(matrix(1, A), astar-abar, matrix(1, A)%*%(pref - pbar) - (astar-abar), XAQ, matrix(1, A)%*%XPQ[prefLOC,])

BINC2 <- c(1, 2, 3, 4, 5)}

xac2 <- lot + XXAQ%*%B[BINC2]

xac2v <- XXAQ%*%s2VAR[BINC2, BINC2]%*%t(XXAQ)

sd <- matrix(sqrt(diag(xac2v)))

ci <- cbind(xac2 - 1.96*sd, xac2 + 1.96*sd)

QuadCrossAge <- cbind(astar, exp(xac2), exp(ci))

dimnames(QuadCrossAge) <- list(c(), c("Age", "Rate", "CILo", "CIHi"))

#

# (6b) Quadratic Cross-Sectional Age Rate Ratios

#

XARQ <- ARR%*%XXAQ

xac2rr <- XARQ%*%B[BINC2]

xac2rrv <- XARQ%*%s2VAR[BINC2,BINC2]%*%t(XARQ)

sd <- matrix(sqrt(diag(xac2rrv)))

ci <- cbind(xac2rr - 1.96*sd, xac2rr + 1.96*sd)

QuadCrossAgeRR <- cbind(astar, exp(xac2rr), exp(ci))

dimnames(QuadCrossAgeRR) <- list(c(), c("Age", "Rate Ratio", "CILo", "CIHi"))

#

# (6c) Complete Cross-Sectional Age Curve

#

if (P>3){

XXA <- cbind(matrix(1, A), astar-abar, matrix(1, A)%*%(pref - pbar) - (astar-abar), XAQ, XAD, matrix(1, A)%*%XP[prefLOC,])

BINCc <- c(1, 2, 3, 4, Pt[[7]], 5, Pt[[8]])}

else{

XXA <- cbind(matrix(1, A), astar-abar, matrix(1, A)%*%(pref - pbar) - (astar-abar), XAQ, XAD, matrix(1, A)%*%XPQ[prefLOC,])

BINCc <- c(1, 2, 3, 4, Pt[[7]], 5)}

xac <- lot + XXA%*%B[BINCc]

xacv <- XXA%*%s2VAR[BINCc, BINCc]%*%t(XXA)

sd <- matrix(sqrt(diag(xacv)))

ci <- cbind(xac - 1.96*sd, xac + 1.96*sd)

CrossAge <- cbind(astar, exp(xac), exp(ci))

if (A==3) {CrossAge <- QuadCrossAge; xacv <- xac2v}

dimnames(CrossAge) <- list(c(), c("Age", "Rate", "CILo", "CIHi"))

#

# (6d) Cross-Sectional Age Rate Ratios

#

XAR <- ARR%*%XXA

xacrr <- XAR%*%B[BINCc]

xacrrv <- XAR%*%s2VAR[BINCc,BINCc]%*%t(XAR)

sd <- matrix(sqrt(diag(xacrrv)))

ci <- cbind(xacrr - 1.96*sd, xacrr + 1.96*sd)

CrossAgeRR <- cbind(astar, exp(xacrr), exp(ci))

if (A==3) {CrossAgeRR <- QuadCrossAgeRR; xacrrv <- xac2rrv}

dimnames(CrossAgeRR) <- list(c(), c("Age", "Rate Ratio", "CILo", "CIHi"))

#

# (5c:6c) Ratio of Longitudinal-to-Cross-Sectional Age Curves

#

# NOTE: XC <- cbind(XCQ, XCD) and XP <- cbind(XPQ, XPD)

# XLA <- cbind(matrix(1, A), a-abar, matrix(1, A)%*%(cref - cbar), XAQ, XAD, matrix(1, A)%*%XC[crefLOC,])

# B[c( 1, 2, 3, 4, Pt[[7]], 6, Pt[[9]])]

# XXA <- cbind(matrix(1, A), a-abar, matrix(1, A)%*%(pref - pbar) - (a-abar), XAQ, XAD, matrix(1, A)%*%XP[prefLOC,])

# B[c( 1, 2, 3, 4, Pt[[7]], 5, Pt[[8]])]

# For coefficients 1, 2, 4, Pt{7} there is no difference between the

# longitudinal and cross-sectional age curves. There is a difference for 3,

# 6, Pt{9}, 5, Pt{8}

if (P>3){

BINCR <- c(3, 6, Pt[[9]], 5, Pt[[8]])

XLX <- cbind(matrix(1, A)*(cref-cbar) - matrix(1, A)*(pref-pbar) + (astar-abar),

matrix(1, A)%*%XC[crefLOC,], -1*matrix(1, A)%*%XP[prefLOC,])}

else{

BINCR <- c(3, 6, Pt[[9]], 5)

XLX <- cbind(matrix(1, A)*(cref-cbar) - matrix(1, A)*(pref-pbar) + (astar-abar),

matrix(1, A)%*%XC[crefLOC,], -1*matrix(1, A)%*%XPQ[prefLOC,])}

lvcrr <- XLX%*%B[BINCR]

lvcrrv <- XLX%*%s2VAR[BINCR,BINCR]%*%t(XLX)

sd <- matrix(sqrt(diag(lvcrrv)))

ci <- cbind(lvcrr - 1.96*sd, lvcrr + 1.96*sd)

Long2CrossRR <- cbind(astar, exp(lvcrr), exp(ci))

dimnames(Long2CrossRR) <- list(c(), c("Age", "Rate Ratio", "CILo", "CIHi"))

#

# (7a) Gradient of Longitudinal Age Curve: linear & Quadratic Components

#

# Scale Factor

Delta <- astar[2] - astar[1]

# Gradient Operator

G <- (1/Delta)*cbind(diag(A-1), matrix(0, A-1))%*%(-diag(A) + as.matrix(bandSparse(A, m = A, 1, matrix(1,A-1))))

lac2_grad <- G%*%lac2

lac2_gradv <- G%*%lac2v%*%t(G)

sd <- matrix(sqrt(diag(lac2_gradv)))

ci <- cbind(lac2_grad - 1.96*sd, lac2_grad + 1.96*sd)

QuadLongAgeGrad <- cbind(astar[1:A-1], 100*(exp(cbind(lac2_grad, ci))-1))

dimnames(QuadLongAgeGrad) <- list(c(), c("Age", "Percent Change per Year of Age", "CILo", "CIHi"))

#

# (7b) Gradient of Longitudinal Age Curve: Higher-Order Components

#

lach <- lot + XAD%*%B[Pt[[7]]]

lachv <- XAD%*%s2VAR[Pt[[7]], Pt[[7]]]%*%t(XAD)

lach_grad <- G%*%lach

lach_gradv <- G%*%lachv%*%t(G)

sd <- matrix(sqrt(diag(lach_gradv)))

ci <- cbind(lach_grad - 1.96*sd, lach_grad + 1.96*sd)

# Multiplier of gradient of linear and quadratic components

HiOrdLongAgeGrad <- cbind(astar[1:A-1], exp(cbind(lach_grad, ci)))

if (A==3) {HiOrdLongAgeGrad <- A3NULL}

dimnames(HiOrdLongAgeGrad) <- list(c(),c("Age", "Rate Multiplier", "CILo", "CIHi"))

#

# (7c) Gradient of Complete Longitudinal Age Curve

#

lac_grad <- G%*%lac

lac_gradv <- G%*%lacv%*%t(G)

sd <- matrix(sqrt(diag(lac_gradv)))

ci <- cbind(lac_grad - 1.96*sd, lac_grad + 1.96*sd)

LongAgeGrad <- cbind(astar[1:A-1], 100*(exp(cbind(lac_grad, ci))-1))

if (A==3) {LongAgeGrad <- QuadLongAgeGrad; lac_gradv <- lac2_gradv}

dimnames(LongAgeGrad) <- list(c(), c("Age", "Percent Change per Year of Age", "CILo", "CIHi"))

#

# (8a) Gradient of Cross-Sectional Curve: linear & Quadratic Components

#

xac2_grad <- G%*%xac2

xac2_gradv <- G%*%xac2v%*%t(G)

sd <- matrix(sqrt(diag(xac2_gradv)))

ci <- cbind(xac2_grad - 1.96*sd, xac2_grad + 1.96*sd)

QuadCrossAgeGrad <- cbind(astar[1:A-1], 100*(exp(cbind(xac2_grad, ci))-1))

dimnames(QuadCrossAgeGrad) <- list(c(), c("Age", "Percent Change per Year of Age", "CILo", "CIHi"))

#

# (8b) Gradient of Cross-Sectional Age Curve: Higher-Order Components

#

# Same as for Longitudinal Age Curve

HiOrdCrossAgeGrad <- HiOrdLongAgeGrad

xach_gradv <- lach_gradv

#

# (8c) Gradient of Complete Cross-Sectional Age Curve

#

xac_grad <- G%*%xac

xac_gradv <- G%*%xacv%*%t(G)

sd <- matrix(sqrt(diag(xac_gradv)))

ci <- cbind(xac_grad - 1.96*sd, xac_grad + 1.96*sd)

CrossAgeGrad <- cbind(astar[1:A-1], 100*(exp(cbind(xac_grad, ci))-1))

if (A==3) {CrossAgeGrad <- QuadCrossAgeGrad; xac_gradv <- xac2_gradv}

dimnames(CrossAgeGrad) <- list(c(), c("Age", "Percent Change per Year of Age", "CILo", "CIHi"))

#

# (9a) Quadratic Fitted Temporal Trends - offset by complete age deviation

# plus CAT times offset of reference value of age versus mean value of age

#

XPTQ <- cbind(matrix(1, P), matrix(1, P)*(aref - abar), ((pstar-pbar) - matrix(1, P)*(aref-abar)), XPQ, matrix(1, P)%*%XA[arefLOC,])

ftt2 <- lot + XPTQ%*%B[c(1, 2, 3, 5, 4, Pt[[7]])]

ftt2v <- XPTQ%*%s2VAR[c(1, 2, 3, 5, 4, Pt[[7]]), c(1, 2, 3, 5, 4, Pt[[7]])]%*%t(XPTQ)

sd <- matrix(sqrt(diag(ftt2v)))

ci <- cbind(ftt2 - 1.96*sd, ftt2 + 1.96*sd)

QuadFittedTemporalTrends <- cbind(pstar, exp(ftt2), exp(ci))

dimnames(QuadFittedTemporalTrends) <- list(c(), c("Period", "Rate", "CILo", "CIHi"))

#

# (9b) Quadratic Period Rate Ratios

#

Xp <- cbind(pstar - pref, XPQ)

TMP <- diag(0, nrow = P)

TMP[,prefLOC] <- 1

PRR <- diag(1, nrow = P) - TMP

XPR <- PRR%*%Xp

prr2 <- XPR%*%B[c(3, 5)]

prr2v <- XPR%*%s2VAR[c(3, 5), c(3, 5)]%*%t(XPR)

sd <- matrix(sqrt(diag(prr2v)))

ci <- cbind(prr2 - 1.96*sd, prr2 + 1.96*sd)

QuadPeriodRR <- cbind(pstar, exp(prr2), exp(ci))

dimnames(QuadPeriodRR) <- list(c(), c("Period", "Rate Ratio", "CILo", "CIHi"))

#

# (9c) Complete Fitted Temporal Trends

#

if (A>3) {

XPT <- cbind(matrix(1, P), matrix(1, P)*(aref - abar), ((pstar-pbar) - matrix(1, P)*(aref-abar)), XPQ, XPD, matrix(1, P)%*%XA[arefLOC,])

INCT <- c(1, 2, 3, 5, Pt[[8]], 4, Pt[[7]])}

else {

XPT <- cbind(matrix(1, P), matrix(1, P)*(aref - abar), ((pstar-pbar) - matrix(1, P)*(aref-abar)), XPQ, XPD, matrix(1, P)%*%XAQ[arefLOC,])

INCT <- c(1, 2, 3, 5, Pt[[8]], 4)}

ftt <- lot + XPT%*%B[INCT]

fttv <- XPT%*%s2VAR[INCT, INCT]%*%t(XPT)

sd <- matrix(sqrt(diag(fttv)))

ci <- cbind(ftt - 1.96*sd, ftt + 1.96*sd)

FittedTemporalTrends <- cbind(pstar, exp(ftt), exp(ci))

if (P==3) {FittedTemporalTrends <- QuadFittedTemporalTrends; fttv <- ftt2v}

dimnames(FittedTemporalTrends) <- list(c(), c("Period", "Rate", "CI Lo", "CI Hi"))

#

# (9d) Complete Period Rate Ratios

#

if (PVP$HiP==1 && P>3){

Xp <- cbind(pstar - pref, XP)

XPR <- PRR%*%Xp

prr <- XPR%*%B[c(3, 5, Pt[[8]])]

prrv <- XPR%*%s2VAR[c(3, 5, Pt[[8]]), c(3, 5, Pt[[8]])]%*%t(XPR)

sd <- matrix(sqrt(diag(prrv)))

ci <- cbind(prr - 1.96*sd, prr + 1.96*sd)

PeriodRR <- cbind(pstar, exp(prr), exp(ci))

dimnames(PeriodRR) <- list(c(), c("Period", "Rate Ratio", "CI Lo", "CI Hi"))

# Wald test - any PeriodRR values different from 1?

INC11 <- c(1:(prefLOC-1), (prefLOC+1):P)

X211 <- t(matrix(prr[INC11]))%*%solve(prrv[INC11, INC11],matrix(prr[INC11]))

df11 <- P - 1

PVAL11 <- pchisq(X211, df11, lower.tail = FALSE)

}

else{

Xp <- cbind(pstar - pref, XPQ)

XPR <- PRR%*%Xp

prr <- XPR%*%B[c(3, 5)]

prrv <- XPR%*%s2VAR[c(3, 5), c(3, 5)]%*%t(XPR)

sd <- matrix(sqrt(diag(prrv)))

ci <- cbind(prr - 1.96*sd, prr + 1.96*sd)

PeriodRR <- cbind(pstar, exp(prr), exp(ci))

dimnames(PeriodRR) <- list(c(), c("Period", "Rate Ratio", "CI Lo", "CI Hi"))

# Wald test - any PeriodRR values different from 1?

INC11 <- c(1, P)

X211 <- t(matrix(prr[INC11]))%*%solve(prrv[INC11, INC11],matrix(prr[INC11]))

df11 <- 2

PVAL11 <- pchisq(X211, df11, lower.tail = FALSE)

}

#

# (10a) Complete Fitted Cohort Pattern centered on the reference age -

# offset by complete age deviation plus LAT times offset of reference value

# of age versus mean value of age

#

if (A>3){

XCT <- cbind(matrix(1, C), matrix(1, C)*(aref - abar), (cstar - cbar), XC, matrix(1, C)%*%XA[arefLOC,])

INCF <- c(1, 2, 3, 6, Pt[[9]], 4, Pt[[7]])}

else{

XCT <-cbind(matrix(1, C), matrix(1, C)*(aref - abar), (cstar - cbar), XC, matrix(1, C)%*%XAQ[arefLOC,])

INCF <- c(1, 2, 3, 6, Pt[[9]], 4)}

fcp <- lot + XCT%*%B[INCF]

fcpv <- XCT%*%s2VAR[INCF,INCF]%*%t(XCT)

sd <- matrix(sqrt(diag(fcpv)))

ci <- cbind(fcp - 1.96*sd, fcp + 1.96*sd)

FittedCohortPattern <- cbind(cstar, exp(fcp), exp(ci))

dimnames(FittedCohortPattern) <- list(c(), c("Cohort", "Rate", "CILo", "CIHi"))

#

# (10b) Complete Cohort Rate Ratios

#

TMP <- diag(0, nrow = C)

TMP[,crefLOC] <- 1

CRR <- diag(1, nrow = C) - TMP

if (PVP$HiC==1){

Xc <- cbind(cstar - cref, XC)

XCR <- CRR%*%Xc

crr <- XCR%*%B[c(3, 6, Pt[[9]])]

crrv <- XCR%*%s2VAR[c(3, 6, Pt[[9]]),c(3, 6, Pt[[9]])]%*%t(XCR)

sd <- matrix(sqrt(diag(crrv)))

ci <- cbind(crr - 1.96*sd, crr + 1.96*sd)

CohortRR <- cbind(cstar, exp(crr), exp(ci))

dimnames(CohortRR) <- list(c(), c("Cohort", "Rate Ratio", "CILo", "CIHi"))

# Wald test - any CohortRR values different from 1?

INC12 <- c(1:(crefLOC-1), (crefLOC+1):C)

X212 <- t(matrix(crr[INC12]))%*%solve(crrv[INC12, INC12],matrix(crr[INC12]))

df12 <- C - 1

PVAL12 <- pchisq(X212, df12, lower.tail = FALSE)

}

else{

Xc <- cbind(cstar - cref, XCQ)

XCR <- CRR%*%Xc

crr <- XCR%*%B[c(3, 6)]

crrv <- XCR%*%s2VAR[c(3, 6),c(3, 6)]%*%t(XCR)

sd <- matrix(sqrt(diag(crrv)))

ci <- cbind(crr - 1.96*sd, crr + 1.96*sd)

CohortRR <- cbind(cstar, exp(crr), exp(ci))

dimnames(CohortRR) <- list(c(), c("Cohort", "Rate Ratio", "CILo", "CIHi"))

# Wald test - any CohortRR values different from 1?

INC12 <- c(1, C)

X212 <- t(matrix(crr[INC12]))%*%solve(crrv[INC12, INC12],matrix(crr[INC12]))

df12 <- 2

PVAL12 <- pchisq(X212, df12, lower.tail = FALSE)

}

#

# (10c) Quadratic Fitted Cohort Pattern centered on the reference age

#

if (A>3) {

XCTQ <- cbind(matrix(1, C), matrix(1, C)*(aref - abar), (cstar - cbar), XCQ, matrix(1, C)%*%XA[arefLOC,])

INCF2 <- c(1, 2, 3, 6, 4, Pt[[7]])}

else {

XCTQ <- cbind(matrix(1, C), matrix(1, C)*(aref - abar), (cstar - cbar), XCQ, matrix(1, C)%*%XAQ[arefLOC,])

INCF2 <- c(1, 2, 3, 6, 4)}

fcp2 <- lot + XCTQ%*%B[INCF2]

fcp2v <- XCTQ%*%s2VAR[INCF2,INCF2]%*%t(XCTQ)

sd <- matrix(sqrt(diag(fcp2v)))

ci <- cbind(fcp2 - 1.96*sd, fcp2 + 1.96*sd)

QuadFittedCohortPattern <- cbind(cstar, exp(fcp2), exp(ci))

dimnames(QuadFittedCohortPattern) <- list(c(), c("Cohort", "Rate", "CILo", "CIHi"))

#

# (10d) Quadratic Cohort Rate Ratios

#

Xc <- cbind(cstar - cref, XCQ)

XCRq <- CRR%*%Xc

crr2 <- XCRq%*%B[c(3, 6)]

crr2v <- XCRq%*%s2VAR[c(3, 6), c(3, 6)]%*%t(XCRq)

sd <- matrix(sqrt(diag(crr2v)))

ci <- cbind(crr2 - 1.96*sd, crr2 + 1.96*sd)

QuadCohortRR <- cbind(cstar, exp(crr2), exp(ci))

dimnames(QuadCohortRR) <- list(c(), c("Cohort", "Rate Ratio", "CILo", "CIHi"))

#

# (11a) Complete Local Drifts

#

XCB <- as.matrix(bdiag(list(XC, 1)))

JP <- matrix(1, P)

SP <- matrix(1:P)

pbars <- (P+1)/2

DP <- (12/(Delta*(P-1)*P*(P+1)))*t(SP - pbars*JP)

KAC <- matrix(0, nrow=A, ncol=C)

for (ag in 1:A) {

# starting at the first age group, you have the most recent set of P cohorts

i0 <- 1+A-ag

i1 <- 1+A-ag+P-1

KAC[ag, i0:i1] <- DP

}

g <- XCB%*%B[c(6, Pt[[9]], 3)]

v <- XCB%*%s2VAR[c(6, Pt[[9]], 3), c(6, Pt[[9]], 3)]%*%t(XCB)

CM <- cbind(KAC, matrix(1, A))

ld <- CM%*%g

ldv <- CM%*%v%*%t(CM)

sd <- matrix(sqrt(diag(ldv)))

ci <- cbind(ld - 1.96*sd, ld + 1.96*sd)

LocalDrifts <- cbind(astar, 100*(exp(ld)-1), 100*(exp(ci)-1))

dimnames(LocalDrifts) <- list(c(),c("Age", "Mean Percent Change per Calendar Year", "CILo", "CIHi"))

#

# (11b) Quadratic Local Drifts

#

XCQB <- as.matrix(bdiag(list(XCQ, 1)))

g2 <- XCQB%*%B[c(6, 3)]

g2v <- XCQB%*%s2VAR[c(6,3), c(6,3)]%*%t(XCQB)

ld2 <- CM%*%g2

ld2v <- CM%*%g2v%*%t(CM)

sd <- matrix(sqrt(diag(ld2v)))

ci <- cbind(ld2 - 1.96*sd, ld2 + 1.96*sd)

QuadLocalDrifts <- cbind(astar, 100*(exp(ld2)-1), 100*(exp(ci)-1))

dimnames(QuadLocalDrifts) <- list(c(), c("Age", "Mean Percent Change per Calendar Year", "CILo", "CIHi"))

#

# (11c) Complete Deflections

#

CM0 <- cbind(KAC, matrix(0, A))

def <- CM0%*%g

defv <- CM0%*%v%*%t(CM0)

sd <- matrix(sqrt(diag(defv)))

ci <- cbind(def - 1.96*sd, def + 1.96*sd)

Deflections <- cbind(astar, def, ci)

dimnames(Deflections) <- list(c(), c("Age", "Deflection of Local Drift From Net Drift", "CILo", "CIHi"))

# Wald Test: Do all Local Drifts equal the Net Drift?

if (PVP$HiC==1){

# If A==P or P==3 the test has A - 1 df, otherwise the test has A df.

if (A==P || P==3) {

X213 <- t(def[1:(A-1)])%*%solve(defv[1:(A-1), 1:(A-1)], def[1:(A-1)])

df13 <- A - 1

}

else {

X213 <- t(def)%*%solve(defv, def)

df13 <- A

}

PVAL13 <- pchisq(X213, df13, lower.tail = FALSE)

}

else{

X213 <- X28

df13 <- df8

PVAL13 <- PVAL8

}

#

# (11d) Quadratic Deflections

#

def2 <- CM0%*%g2

def2v <- CM0%*%g2v%*%t(CM0)

# If A is odd, the arithmetic mean of a coincides with the middle observed

# value of a. The variances and covariances of def2 should be exactly equal

# to 0 at this point, but values may compute to -eps or smaller, resulting

# in imaginary sqrt values. The fix is to plug in the theoretical value of

# 0. Also, the value of def2 at this point should be set to 0.

if (A %% 2) {

def2v[arefLOC,] <- 0

def2v[,arefLOC] <- 0

def2[arefLOC] <- 0

}

sd <- matrix(sqrt(diag(def2v)))

ci <- cbind(def2 - 1.96*sd, def2 + 1.96*sd)

QuadDeflections <- cbind(astar, def2, ci)

dimnames(QuadDeflections) <- list(c(), c("Age", "Deflection of Local Drift From Net Drift", "CILo", "CIHi"))

#

# Perturbations and Gradient Shifts

#

JA <- matrix(1, A)

SA <- matrix(1:A)

abars <- (A+1)/2

DA <- (12/(Delta*(A-1)*A*(A+1)))*t(SA - abars*JA)

KPC <- matrix(0, nrow=P, ncol=C)

for (pk in 1:P) {

for (ck in 1:C) {

if ((ck >= 1) && (ck < pk)) {

KPC[pk, ck] = 0 }

else if ((ck >= pk) && (ck <= pk + A -1)) {

# Values are loaded in sequence {A, A-1, ..., 1}

KPC[pk, ck] <- -DA[pk - ck + A] }

else {

KPC[pk, ck] <- 0

}

}

}

#

# (12a) Complete Perturbations

#

CMp <- -KPC

pert <- CMp%*%cd

pertv <- CMp%*%cdv%*%t(CMp)

sd <- matrix(sqrt(diag(pertv)))

ci <- cbind(pert - 1.96*sd, pert + 1.96*sd)

Perturbations <- cbind(pstar, 100*(exp(pert)-1), 100*(exp(ci)-1))

dimnames(Perturbations) <- list(c(), c("Period", "Perturbation from CAT", "CILo", "CIHi"))

#

# (12b) Quadratic Perturbations

#

pert2 <- CMp%*%cd2

pert2v <- CMp%*%cd2v%*%t(CMp)

# If P is odd, the arithmetic mean of p coincides with the middle observed

# value of p. The variance and covariances of pert2 should be exactly equal

# to 0 at this point, but may compute to -eps or smaller, resulting in

# imaginary sqrt values. The fix is to plug in the theoretical value of 0.

# Also, the value of pert2 at this point should be set to 0.

if (P %% 2)

pert2v[prefLOC, ] <- 0

pert2v[, prefLOC] <- 0

pert2[prefLOC] <- 0

end

sd <- matrix(sqrt(diag(pert2v)))

ci <- cbind(pert2 - 1.96*sd, pert2 + 1.96*sd)

QuadPerturbations <- cbind(pstar, 100*(exp(pert2)-1), 100*(exp(ci)-1))

dimnames(QuadPerturbations) <- list(c(), c("Period", "Perturbation from CAT", "CILo", "CIHi"))

#

# (12c) Complete Gradient Shifts

#

XCBp <- as.matrix(bdiag(list(XC, 1, 1)))

# cohort deviations on top of LAT on top of NetDrift

g <- XCBp%*%B[c(6, Pt[[9]], 2, 3)]

v <- XCBp%*%s2VAR[c(6, Pt[[9]], 2, 3), c(6, Pt[[9]], 2, 3)]%*%t(XCBp)

CM0 <- cbind(-KPC, matrix(1, P), -matrix(1, P))

gs <- CM0%*%g

gsv <- CM0%*%v%*%t(CM0)

sd <- matrix(sqrt(diag(gsv)))

ci <- cbind(gs - 1.96*sd, gs + 1.96*sd)

GradientShifts <- cbind(pstar, 100*(exp(gs)-1), 100*(exp(ci)-1))

dimnames(GradientShifts) <- list(c(), c("Period", "Mean Percent Change per Year of Age", "CILo", "CIHi"))

# Wald test: Do all Gradient Shifts equal the CAT?

if (PVP$HiC==1){

dgs <- -KPC%*%cd

dgsv <- KPC%*%cdv%*%t(KPC)

# If A==P the test has P - 1 df, otherwise the test has P df.

if (A == P) {

X214 <- t(dgs[1:(P-1)])%*%solve(dgsv[1:(P-1), 1:(P-1)], dgs[1:(P-1)])

df14 <- P - 1

}

else {

X214 <- t(dgs)%*%solve(dgsv, dgs)

df14 <- P

}

PVAL14 <- pchisq(X214, df14, lower.tail = FALSE)

}

else{

X214 <- X28

df14 <- df8

PVAL14 <- PVAL8

}

#

# (12d) Quadratic Gradient Shifts

#

XCQp <- as.matrix(bdiag(list(XCQ, 1, 1)))

g2 <- XCQp%*%B[c(6, 2, 3)]

g2v <- XCQp%*%s2VAR[c(6, 2, 3), c(6, 2, 3)]%*%t(XCQp)

gs2 <- CM0%*%g2

gs2v <- CM0%*%g2v%*%t(CM0)

sd <- matrix(sqrt(diag(gs2v)))

ci <- cbind(gs2 - 1.96*sd, gs + 1.96*sd)

QuadGradientShifts <- cbind(pstar, 100*(exp(gs2)-1), 100*(exp(ci)-1))

dimnames(QuadGradientShifts) <- list(c(), c("Period", "Mean Percent Change per Year of Age", "CILo", "CIHi"))

WaldTests <- matrix(

c(X21, df1, PVAL1, X22, df2, PVAL2,

X23, df3, PVAL3, X24, df4, PVAL4,

X25, df5, PVAL5, X26, df6, PVAL6,

X27, df7, PVAL7, X28, df8, PVAL8,

X29, df9, PVAL9, X210, df10, PVAL10,

X211, df11, PVAL11, X212, df12, PVAL12,

X213, df13, PVAL13, X214, df14, PVAL14), 14, 3, byrow = TRUE)

dimnames(WaldTests) <- list(

c("NetDrift = 0",

"THETAa = 0",

"All Higher-Order Age Deviations = 0",

"All Age Deviations = 0",

"THETAp = 0",

"All Higher-Order Period Deviations = 0",

"All Period Deviations = 0",

"THETAc = 0",

"All Higher-Order Cohort Deviations = 0",

"All Cohort Deviations = 0",

"All Period RR = 1",

"All Cohort RR = 1",

"All Local Drifts = Net Drift",

"All Gradient Shifts = CAT"),

c("X2", "df", "P-Value"))

CombinationTests <- matrix(NaN, nrow = 4)

CombinationTests[1] <- min(c(2*PVAL5, 2*PVAL6, 1))

CombinationTests[2] <- min(c(3*PVAL1, 3*PVAL5, 3*PVAL6, 1))

CombinationTests[3] <- min(c(2*PVAL8, 2*PVAL9, 1))

CombinationTests[4] <- min(c(3*PVAL1, 3*PVAL8, 3*PVAL9, 1))

dimnames(CombinationTests) <- list(c("All Period Deviations = 0",

"All PRR = 1 <=> FTT = constant",

"All Cohort Deviations = 0",

"All CRR = 1 <=> FCP = constant"), c())

# Matrix that converts from C-2 cohort parameters to C cohort deviations.

# (For use outside this function).

nc <- D$nc

vC <- (1/(cstar[C]-cstar[1]))*( cstar[1]*matrix(nc[2:(C-1)]) - matrix(cstar[2:(C-1)]*nc[2:(C-1)]))

v1 <- matrix(-nc[2:(C-1)]) - vC

H <- rbind(t(v1), diag(C - 2), t(vC))

Variances <- list(

ad = adv ,

ad2 = ad2v ,

adh = adhv ,

pd = pdv ,

pd2 = pd2v ,

pdh = pdhv ,

cd = cdv ,

cd2 = cd2v ,

cdh = cdhv ,

lac = lacv ,

lac2 = lac2v ,

lacrr = lacrrv ,

lac2rr = lac2rrv ,

xac = xacv ,

xac2 = xac2v ,

xacrr = xacrrv ,

xac2rr = xac2rrv ,

lvcrr = lvcrrv ,

lac_grad = lac_gradv ,

lac2_grad = lac2_gradv,

lach_grad = lach_gradv,

xac_grad = xac_gradv ,

xac2_grad = xac2_gradv,

xach_grad = lach_gradv,

ftt = fttv ,

ftt2 = ftt2v ,

prr = prrv ,

prr2 = prr2v ,

fcp = fcpv ,

fcp2 = fcp2v ,

crr = crrv ,

crr2 = crr2v ,

ld = ldv ,

ld2 = ld2v ,

def = defv ,

def2 = def2v ,

gs = gsv ,

gs2 = gs2v ,

pert = pertv ,

pert2 = pert2v )

Matrices <- list(

X = D$X ,

XCO = XCO ,

XAQ = XAQ ,

XAD = XAD ,

XPQ = XPQ ,

XPD = XPD ,

XCQ = XCQ ,

XCD = XCD ,

XLA = XLA ,

XLAQ = XLAQ,

XXA = XXA ,

XXAQ = XXAQ,

XPT = XPT ,

XPTQ = XPTQ,

XPR = XPR ,

XCR = XCR ,

XCB = XCB ,

CM = CM ,

XCT = XCT ,

XCTQ = XCTQ,

XLX = XLX ,

LAR = LAR ,

KAC = KAC ,

KPC = KPC ,

GA = G ,

H = H )

M <- list(

Inputs = PVP ,

FittedRates = FittedRates ,

Coefficients = Coefficients ,

NetDrift = NetDrift ,

GlobalCurvature = GlobalCurvature ,

QuadAgeDeviations = QuadAgeDeviations ,

HiOrdAgeDeviations = HiOrdAgeDeviations ,

AgeDeviations = AgeDeviations ,

QuadPerDeviations = QuadPerDeviations ,

HiOrdPerDeviations = HiOrdPerDeviations ,

PerDeviations = PerDeviations ,

QuadCohDeviations = QuadCohDeviations ,

HiOrdCohDeviations = HiOrdCohDeviations ,

CohDeviations = CohDeviations ,

LongAge = LongAge ,

LongAgeRR = LongAgeRR ,

QuadLongAge = QuadLongAge ,

QuadLongAgeRR = QuadLongAgeRR ,

CrossAge = CrossAge ,

CrossAgeRR = CrossAgeRR ,

QuadCrossAge = QuadCrossAge ,

QuadCrossAgeRR = QuadCrossAgeRR ,

Long2CrossRR = Long2CrossRR ,

QuadLongAgeGrad = QuadLongAgeGrad ,

HiOrdLongAgeGrad = HiOrdLongAgeGrad ,

LongAgeGrad = LongAgeGrad ,

QuadCrossAgeGrad = QuadCrossAgeGrad ,

HiOrdCrossAgeGrad = HiOrdCrossAgeGrad ,

CrossAgeGrad = CrossAgeGrad ,

QuadFittedTemporalTrends = QuadFittedTemporalTrends,

QuadPeriodRR = QuadPeriodRR ,

FittedTemporalTrends = FittedTemporalTrends ,

PeriodRR = PeriodRR ,

FittedCohortPattern = FittedCohortPattern ,

CohortRR = CohortRR ,

QuadFittedCohortPattern = QuadFittedCohortPattern ,

QuadCohortRR = QuadCohortRR ,

LocalDrifts = LocalDrifts ,

QuadLocalDrifts = QuadLocalDrifts ,

Deflections = Deflections ,

QuadDeflections = QuadDeflections ,

Perturbations = Perturbations ,

QuadPerturbations = QuadPerturbations ,

GradientShifts = GradientShifts ,

QuadGradientShifts = QuadGradientShifts ,

WaldTests = WaldTests ,

CombinationTests = CombinationTests ,

Variances = Variances ,

Matrices = Matrices ,

APCModel = apcM ,

Pt = Pt ,

nc = nc )

M

}

checkPVPPAIRS <- function(R, OverDispersion = 1, offset_tick = 10^5, zero_fill = 0.1, RVals = c(NaN, NaN, NaN), HiC = TRUE, HiP = TRUE, HiA = TRUE)

{

# Pre-process input data and parameters

D <- rates2data_set(R)

if (all(is.nan(RVals))) {

# Calculate default reference values

A <- length(D$a)

P <- length(D$p)

aref <- floor((A+1)/2)

pref <- floor((P+1)/2)

cref <- pref - aref + A

ageref <- D$a[aref]

perref <- D$p[pref]

cohref <- D$c[cref]

RVals <- c(ageref, perref, cohref)

}

else {

# Valdidate user-supplied reference values

RVals <- floor(RVals)

TA <- is.element(RVals[1], D$a)

if (!TA) {

RVals[1] <- floor(RVals[1]) + 0.5

TA <- is.element(RVals[1], D$a)

}

TB <- is.element(RVals[2], D$p)

if (!TB) {

RVals[2] <- floor(RVals[2]) + 0.5

TB <- is.element(RVals[2], D$p)

}

TC <- is.element(RVals[3], D$c)

if (!TC) {

RVals[3] <- floor(RVals[3]) + 0.5

TC <- is.element(RVals[3], D$c)

}

if (!(TA && TB && TC))

stop("Invalid Age, Period, or Cohort reference value.")

end

}

# Replace 0 events with zero_fill value.

e <- matrix(D$DATA[,4])

e[e==0] <- zero_fill

D$DATA[,4] <- e

PVP <- list(D = D,

RVals = RVals,

OverDispersion = OverDispersion,

offset_tick = offset_tick,

zero_fill = zero_fill,

HiC = HiC,

HiP = HiP,

HiA = HiA)

PVP

}

APCFIT = function(PVP, X)

{

# Fit APC model by weighted least squares

A <- length(PVP$D$a)

P <- length(PVP$D$p)

n <- nrow(X)

p <- ncol(X)

p0 = ncol(X)

Y <- PVP$D$DATA[,4:5];

offset <- matrix(log(Y[,2]))

y <- matrix(Y[,1])

ly <- log(y)

W <- y

WX <- (W%*%matrix(1,ncol=p0))*X

z <- (ly - offset)

XTWX <- t(X)%*%WX

B <- solve(t(X)%*%WX,t(WX)%*%z)

V <- solve(t(X)%*%WX)

u <- matrix(Y[,2]*exp(X%*%B))

wr2 <- matrix(W*(z-X%*%B)^2)

DEVRESIDS <- sign(y-u)*sqrt(wr2)

DEV <- sum(wr2)

if (PVP$OverDispersion==1){

s2 <- max(1, DEV/(n-p0))

} else {s2 <- 1}

s2V = s2*V

APCMODEL <- list(B = B,

s2 = s2,

s2VAR = s2V,

DEV = DEV,

DevResids = DEVRESIDS)

APCMODEL

}

designmatrix <- function(PVP)

{

# Calculate design matrix for APC model

N <- nrow(PVP$D$DATA)

J <- matrix(1, nrow = N)

I.N <- diag(N)

astar <- matrix(PVP$D$DATA[,1])

astarbar <- mean(astar)

astar_0 <- astar - astarbar

A <- length(PVP$D$a)

pstar <- matrix(PVP$D$DATA[,2])

pstarbar <- mean(pstar)

pstar_0 <- pstar - pstarbar

P <- length(PVP$D$p)

cstar <- matrix(PVP$D$DATA[,3])

cstarbar <- pstarbar - astarbar

cstar_0 <- cstar - cstarbar

C <- length(PVP$D$c)

# Age

Ad <- kronecker(matrix(1, nrow=P), diag(A))

# Per

Pd <- kronecker(diag(P), matrix(1, nrow=A))

# Coh

Cd <- matrix(NaN,nrow=N,ncol=C)

for (i in 1:C)

Cd[,i] <- cstar == PVP$D$c[i]

end

nc <- t( diag( t(Cd)%*%Cd ) )

# Projection matrix orthogonal to intercept, linear age, and quadratic age

Delta <- astar[2] - astar[1]

a00 <- astar[1] - 0.5*Delta

if (A>=3) {

qa2 <- astar^2 - (Delta*A + 2*a00)*astar + (a00 + Delta*A/2)^2 - ((Delta^2)/12)*(A-1)*(A+1)

Xa2 <- cbind(J, astar_0, qa2)

xtxi <- (1/P)*diag( 1/ c(A, (Delta^2/12)*(A-1)*A*(A+1), (Delta^4/180)*(A-2)*(A-1)*A*(A+1)*(A+2) ) )

Ra2 <- xtxi%*%t(Xa2)

PA12 <- Xa2%*%Ra2

XAD12 <- I.N - PA12

}

else {

qa2 <- matrix(0, N)

Xa2 <- cbind(J, astar_0)

xtxi <- (1/P)*diag(1/ c(A, (Delta^2/12)*(A-1)*A*(A+1)) )

Ra2 <- xtxi%*%t(Xa2)

PA12 <- Xa2%*%Ra2

XAD12 <- I.N - PA12

}

# Projection matrix orthogonal to intercept, linear period, and quadratic period

p00 <- pstar[1] - 0.5*Delta

if (P >= 3) {

qp2 <- pstar^2 - (Delta*P + 2*p00)*pstar + (p00 + Delta*P/2)^2 - ((Delta^2)/12)*(P-1)*(P+1)

Xp2 <- cbind(J, pstar_0, qp2)

xtxi <- (1/A)*diag( 1/ c(P, (Delta^2/12)*(P-1)*P*(P+1), (Delta^4/180)*(P-2)*(P-1)*P*(P+1)*(P+2) ) )

Rp2 <- xtxi%*%t(Xp2)

PP12 <- Xp2%*%Rp2

XPD12 <- I.N - PP12

}

else {

qp2 <- pstar^2 - (Delta*P + 2*p00)*pstar + (p00 + Delta*P/2)^2 - ((Delta^2)/12)*(P-1)*(P+1)

Xp2 <- cbind(J, pstar_0)

xtxi <- (1/A)*diag( 1/ c(P, (Delta^2/12)*(P-1)*P*(P+1) ) )

Rp2 <- xtxi%*%t(Xp2)

PP12 <- Xp2%*%Rp2

XPD12 <- I.N - PP12

}

c00 <- p00 - a00

qc2 <- cstar^2 - (Delta*(A+P) + 2*(c00 - Delta*A))*cstar + (c00 - Delta*A)^2 + (A+P)*Delta*cstarbar - ((Delta^2)/6)*(2*A^2 + 3*A*P + 2*P^2 - 1)

Xc2 <- cbind(J, cstar_0, qc2)

xtxi <- (1/(A*P))*diag( 1/ c(1, (Delta^2/12)*(A^2 + P^2 - 2), (Delta^4/180)*( (A^2 + P^2)^2 - 10*(A^2 + P^2) + 3*A^2*P^2 + 13 ) ) )

Rc2 <- xtxi%*%t(Xc2)

PC12 <- Xc2%*%Rc2

XCD12 <- I.N - PC12

# Orthogonalize the deviations

Ad0 <- XAD12%*%Ad

Pd0 <- XPD12%*%Pd

Cd0 <- XCD12%*%Cd

if (A>3) {

aCOL <- 2:(A-2)

}

else {

# Ad0 column space is empty

aCOL <- NULL

}

if (P>3){

pCOL <- 2:(P-2)

}

else {

# Pd0 column space is empty

pCOL <- NULL

}

X <- cbind(J, astar_0, cstar_0, qa2, qp2, qc2, Ad0[,aCOL], Pd0[,pCOL], Cd0[,2:(C-2)])

# Pointers for Higher-Order Deviations:

#

# * For age:

# 1:(A - 3) shift forward by 6 -> (1 + 6):(A - 3 + 6) = 7:(A + 3)

# * For period:

# 1:(P - 3) shift forward by 6 + (A - 3) ->

# (1 + 6 + A - 3):(P - 3 + 6 + A - 3) = (A + 4):(A + P)

# * For cohort:

# 1:(C - 3) shift forward by 6 + (A - 3) + (P - 3) ->

# (1 + 6 + (A - 3) + (P - 3)):(C - 3) + 6 + (A - 3) + (P - 3) =

# (A + P + 1):(A + P + C - 3)

Pt <- vector("list", 9)

Pt[[1]] <- 1

Pt[[2]] <- 2

Pt[[3]] <- 3

Pt[[4]] <- 4

Pt[[5]] <- 5

Pt[[6]] <- 6

Pt[[7]] <- 7:(A+3)

Pt[[8]] <- (A+4):(A+P)

Pt[[9]] <- (A+P+1):(A+P+C-3)

if (A==3) {

# 7:(A+3) runs backwards but (A+4) = 7

# need to reset Pt[[7]] to NaN but Pt{8} & Pt{9} are correct

Pt[[7]] <- NaN

}

if (P==3) {

Pt[[8]] <- NaN

}

# Pointer for cohort effects, (last f/u, South+East edges of Lexis)

INCc <- c(A*(1:P), P*A-(1:(A-1)))

# Pointer for age effects, (South edge of Lexis)

INCa <- ((P-1)*A+1):(P*A)

# Pointer for period effects (East edge of Lexis)

INCp <-A*(1:P)

D <- list(X = X, INCa = INCa, INCp = INCp, INCc = INCc, Pt = Pt, nc = nc)

D

}

rates <- function(EVENTS, OFFSET,

fullname = character(0),

label = character(0),

description = character(0),

event_label = "events",

offset_units = "offset units",

offset_tick = 100000,

api = c(NaN, NaN, NaN),

ages = NaN,

periods = NaN)

{

###

# Validate EVENTS and OFFSET

###

if (is.matrix(EVENTS) && all(EVENTS >= 0))

{

A <- nrow(EVENTS)

P <- ncol(EVENTS)

}

else

{

stop("events must be a matrix of non-negative values.")

}

if (!(is.matrix(OFFSET) && all(OFFSET >= 0) && nrow(OFFSET)==A && ncol(OFFSET)==P))

{

stop("offset must be a matrix of non-negative values the same size as events.")

}

###

# Validate ages and periods specified by api or ages/periods combination.

###

if (!all(is.nan(api)))

{

if ( !( length(api==3) && is.numeric(api) ) )

{stop("api must be a 3 element vector.")}

if (!all(api==round(api)))

{stop("api values must be single-years.")}

a0 <- api[1]

p0 <- api[2]

INTERVAL <- api[3]

ages <- a0 + INTERVAL*(0:A)

periods <- p0 + INTERVAL*(0:P)

a <- ages[1:A-1] + 0.5*INTERVAL

p <- periods[1:P-1] + 0.5*INTERVAL

}

else

{

if (! ( !any(is.nan(ages)) && !any(is.nan(periods)) &&

all(ages==round(ages)) && all(periods==round(periods)) &&

(length(ages)==A+1) && (length(periods)==P+1) ) )

{stop("Invalid cutpoints for ages or periods.")}

}

###

# Validate text inputs.

###

if (!is.character(fullname))

{stop('fullname must be a character string.')}

else

{fullname <- gsub("^\\s+|\\s+$", '', fullname)}

if (length(fullname)==0)

{fullname <- paste(c(toString(A), '-by-', toString(P), ' rates object'), collapse = "")}

if (length(description)==0)

{description <- paste(c('Created ', date()), collapse = "")}

if (!is.character(event_label))

{stop('event_label must be a character string.')}

if (!is.character(offset_units))

{stop('offset_units must be a character string.')}

if (length(label)==0)

{label <- fullname}

#aL <- as.character(seq(from = ages[1], to = ages[A], by = 2))

#aH <- as.character(seq(from = ages[2], to = ages[A+1], by = 2))

da <- ages[2:(A+1)] - ages[1:A]

if (!all(da==1))

{ aL <- as.character(seq(from = ages[1], to = ages[A], by = 2))

aH <- as.character(seq(from = ages[2], to = ages[A+1], by = 2))

age_labels <- paste(cbind(aL), ' - ', cbind(aH))}

else

{aL <- as.character(seq(from = ages[1], to = ages[A], by = 1))

age_labels <- aL}

#pL <- as.character(seq(from = periods[1], to = periods[P], by = 2))

#pH <- as.character(seq(from = periods[2], to = periods[P+1], by = 2))

dp <- periods[2:(P+1)] - periods[1:P]

if (!all(dp==1))

{ pL <- as.character(seq(from = periods[1], to = periods[P], by = 2))

pH <- as.character(seq(from = periods[2], to = periods[P+1], by = 2))

per_labels <- paste(cbind(pL), ' - ', cbind(pH))}

else

{ pL <- as.character(seq(from = periods[1], to = periods[P], by = 1))

per_labels <- pL}

a <- ages[1:A] + da/2

p <- periods[1:P] + dp/2

R <- list(fullname = fullname,

label = label,

description = description,

events = EVENTS,

event_label = event_label,

offset = OFFSET,

offset_units = offset_units,

offset_tick = offset_tick,

ages = ages,

age_labels = age_labels,

a = a,

periods = periods,

per_labels = per_labels,

p = p)

R

}

rates2data_set <- function(R) {

A <- nrow(R$events)

P <- ncol(R$events)

da <- R$ages[2:(A+1)] - R$ages[1:A]

D.a <- R$ages[1:A] + 0.5*da

dp <- R$periods[2:(P+1)] - R$periods[1:P]

D.p <- R$periods[1:P] + 0.5*dp

ADATA <- kronecker(matrix(1, nrow=P), matrix(D.a, nrow=A))

PDATA <- kronecker(matrix(D.p, nrow=P), matrix(1, nrow=A))

CDATA <- PDATA - ADATA

D.c <- sort(c(unique(CDATA)))

E <- c(R$events)

O <- c(R$offset)

D.DATA = cbind(ADATA, PDATA, CDATA, E, O)

colnames(D.DATA)<-c("Age","Period","Cohort","Events","Offset")

D <- list(name = R$name,

description = R$description,

DATA = D.DATA,

a = D.a,

p = D.p,

c = D.c)

D

}

rates2csv <- function(R, FILE = "rates.csv")

{

A <- nrow(R$events)

P <- ncol(R$events)

comma = ','

commas <- paste(replicate(P-1, ','), collapse="")

# fprintf(fid, ['Title: ' R.fullname commas '\n']);

s1 <- paste(c('Title: ', R$fullname, commas), collapse = "")

s2 <- paste(c('Description: ', R$description, commas), collapse = "")

s3 <- paste(c('Start Year: ', toString(R$p[1]), commas), collapse = "")

s4 <- paste(c('Start Age: ', toString(R$a[1]), commas), collapse = "")

s5 <- paste(c('Interval (Years): ', toString(R$a[2]-R$a[1]), commas), collapse = "")

DATA <- matrix(NaN, A, 2*P)

DATA[,seq(1, 2*P-1, by = 2)] <- R$events

DATA[,seq(2, 2*P, by = 2)] <- R$offset

cat(s1, s2, s3, s4, s5, file = FILE, sep = "\n", append = FALSE)

write(t(DATA), file = FILE, append = TRUE, sep = ',', ncolumns = 2*P)

Fout <- list(s1 = s1, s2 = s2, s3 = s3, s3 = s4, s5 = s5, DATA = DATA)

Fout

}

simple_csv2rates <- function(FILE,StartYear,StartAge,Interval,fullname,description)

{

# DATA is a data.frame

DATA <- read.table(FILE, header = FALSE, sep = ',')

PP <- ncol(DATA)

A <- nrow(DATA)

E = as.matrix(DATA[, seq(1, PP-1, by = 2)])

dimnames(E) <- NULL

O = as.matrix(DATA[, seq(2, PP, by = 2)])

dimnames(O) <- NULL

a <- seq(from = StartAge, by = Interval, to = StartAge + Interval*A)

p <- seq(from = StartYear, by = Interval, to = StartYear + Interval*PP/2)

R <- rates(E, O,

fullname = fullname,

description = description,

ages = a,

periods = p)

R

}

prepare_rates <- function(DATA,StartYear,StartAge,Interval,fullname,description)

{

# DATA is a data.frame

PP <- ncol(DATA)

A <- nrow(DATA)

E = as.matrix(DATA[, seq(1, PP-1, by = 2)])

dimnames(E) <- NULL

O = as.matrix(DATA[, seq(2, PP, by = 2)])

dimnames(O) <- NULL

a <- seq(from = StartAge, by = Interval, to = StartAge + Interval*A)

p <- seq(from = StartYear, by = Interval, to = StartYear + Interval*PP/2)

R <- rates(E, O,

fullname = fullname,

description = description,

ages = a,

periods = p)

R

}

type <- function(R, comp = 'r')

{

A <- nrow(R$events)

P <- ncol(R$events)

if (comp == "r") {

Tout <- R$offset_tick*R$events/R$offset

dn <- paste('Rates - ', R$fullname)

} else if (comp == "e") {

Tout <- R$events

dn <- paste("Events -", R$fullname)

} else if (comp == "o") {

Tout <- R$offset

dn <- paste('Offset - ', R$fullname)

} else if (comp == "eo") {

DATA <- matrix(NaN, nrow = A, ncol = 2*P)

DATA[,seq.int(1, 2*P, 2)]<-R$events

DATA[,seq.int(2, 2*P, 2)]<-R$offset

Tout <- DATA

dn <- paste("Events & Offset -", R$fullname)

} else if (comp == "er") {

r <- R$offset_tick*R$events/R$offset

DATA <- matrix(NaN, nrow = A, ncol = 2*P)

DATA[,seq.int(1, 2*P, 2)]<-R$events

DATA[,seq.int(2, 2*P, 2)]<-r

Tout <- DATA

dn <- paste("Events and Rates - ", R$fullname)

} else if (comp == "eor") {

r <- R$offset_tick*R$events/R$offset

DATA <- matrix(NaN, nrow = A, ncol = 3*P)

DATA[,seq.int(1, 3*P, 3)]<-R$events

DATA[,seq.int(2, 3*P, 3)]<-R$offset

DATA[,seq.int(3, 3*P, 3)]<-r

Tout <- DATA

dn <- paste("Events, offset, and Rates - ", R$fullname)

} else if (comp == "rci") {

r <- R$offset_tick*R$events/R$offset

v <- (R$offset_tick^2)*R$events/R$offset^2

cilo <- r - 1.96*sqrt(v)

cilo[cilo<0] <- 0

cihi <- r + 1.96*sqrt(v)

DATA <- matrix(NaN, nrow = A, ncol = 3*P)

DATA[,seq.int(1, 3*P, 3)]<-r

DATA[,seq.int(2, 3*P, 3)]<-cilo

DATA[,seq.int(3, 3*P, 3)]<-cihi

Tout <- DATA

dn <- paste("Rates and 95% CI - ", R$fullname)

} else {

}

Tout <- list(name = dn, DATA = Tout, ages = R$ages, periods = R$periods)

Tout

}

csv2rates <- function(FILE)

{

StartYear <- 0

StartAge <- 0

Interval <- 1

header <- scan(FILE, nlines = 5, what = character(0), sep = '/', quiet = 1)

# Strip any excess delimeters

header <- gsub(",", "", header)

# Strip leading and trailing white space

header <- gsub("^\\s+|\\s+$", "", header)

H = length(header)

k <- 0

for (h in 1:H) {

headerh = header[h]

nc = nchar(headerh)

f <- regexpr("Title:", headerh, ignore.case = TRUE)

d <- regexpr("Description:", headerh, ignore.case = TRUE)

p <- regexpr("Start Year:", headerh, ignore.case = TRUE)

a <- regexpr("Start Age:", headerh, ignore.case = TRUE)

i <- regexpr("Interval \\(Years\\):", headerh, ignore.case = TRUE)

if (f==1)

{

fullname <- gsub("^\\s+|\\s+$", "", substr(headerh, attr(f, "match.length")+1, nc))

k <- k + 1

}

if (d==1)

{

description <- gsub("^\\s+|\\s+$", "", substr(headerh, attr(d, "match.length")+1,nc))

k <- k + 1

}

if (p==1)

{

StartYear <- as.numeric(substr(headerh, attr(p, "match.length")+1,nc))

k <- k + 1

}

if (a==1)

{

StartAge <- as.numeric(substr(headerh, attr(a, "match.length")+1,nc))

k <- k + 1

}

if (i==1)

{

Interval <- as.numeric(substr(headerh, attr(i, "match.length")+1,nc))

k <- k + 1

}

}

# DATA is a data.frame

DATA <- read.table(FILE, skip = k, header = FALSE, sep = ',')

PP <- ncol(DATA)

A <- nrow(DATA)

E = as.matrix(DATA[, seq(1, PP-1, by = 2)])

dimnames(E) <- NULL

O = as.matrix(DATA[, seq(2, PP, by = 2)])

dimnames(O) <- NULL

a <- seq(from = StartAge, by = Interval, to = StartAge + Interval*A)

p <- seq(from = StartYear, by = Interval, to = StartYear + Interval*PP/2)

R <- rates(E, O,

fullname = fullname,

description = description,

ages = a,

periods = p)

R

}

plot.apc1 <- function(M)

{

# Plot first set of age-period-cohort estimable functions.

par(mfrow = c(4,3))

DATA <- cbind(matrix(M$LongAge[,1]), (M$LongAge[,c(2,3,4)]))

dimnames(DATA) <- list(c(), c("Age", "Rate", "CILo", "CIHi"))

pcurve(DATA, col = "darkred", colf = "pink", lwd = 3, cex = 1.0, pch = 21)

title(main = "Longitudinal Age Curve", cex.main = 1)

DATA <- cbind(matrix(M$CrossAge[,1]), (M$CrossAge[,c(2,3,4)]))

dimnames(DATA) <- list(c(), c("Age", "Rate", "CILo", "CIHi"))

pcurve(DATA, lwd = 3, col = "darkred", colf = "pink", cex = 1.0, pch = 21)

title(main = "Cross-Sectional Age Curve", cex.main = 1)

pcurve(M$Long2CrossRR, lwd = 3, col = "darkred", colf = "pink", cex = 1.0, pch = 21)

abline(1,0, lty = 3)

title(main = "Long vs. Cross RR", cex.main = 1)

pcurve(M$FittedTemporalTrends, col = "steelblue4", colf = "slategray1", lwd = 3, cex = 1.0, pch = 21)

title(main = "Fitted Temporal Trends", cex.main = 1)

pcurve(M$PeriodRR, col = "steelblue4", colf = "slategray1", lwd = 3, cex = 1.0, pch = 21)

abline(1, 0, lty = 3)

title(main = "Period RR", cex.main = 1)

pcurve(M$CohortRR, col = "seagreen4", colf = "darkseagreen1", lwd = 3, cex = 1.0, pch = 21)

abline(1, 0, lty = 3)

title(main = "Cohort RR", cex.main = 1)

pcurve(M$LocalDrifts, col = "black", colf = "grey88", lwd = 3, cex = 1.0, pch = 21)

abline(0, 0, lty = 3)

title(main = "Local Drifts", cex.main = 1)

pcurve(M$AgeDeviations, col = "darkred", colf = "pink", lwd = 3, cex = 1.0, pch = 21)

abline(0, 0, lty = 3)

title(main = "Age Deviations", cex.main = 1)

pcurve(M$PerDeviations, col = "steelblue4", colf = "slategray1", lwd = 3 , cex = 1.0, pch = 21)

abline(0, 0, lty = 3)

title(main = "Period Deviations", cex.main = 1)

pcurve(M$CohDeviations, col = "seagreen4", colf = "darkseagreen1", lwd = 3, cex = 1.0, pch = 21)

abline(0, 0, lty = 3)

title(main = "Cohort Deviations", cex.main = 1)

pcurve(M$FittedCohortPattern, col = "seagreen4", colf = "darkseagreen1", lwd = 3, cex = 1.0, pch = 21)

abline(0, 0, lty = 3)

title(main = "Fitted Cohort Pattern", cex.main = 1)

}

plot.apc2 <- function(M)

{

# Plot second set of age-period-cohort estimable functions.

par(mfrow = c(4,3))

DATA <- cbind(matrix(M$QuadLongAge[,1]), (M$QuadLongAge[,c(2,3,4)]))

dimnames(DATA) <- list(c(), c("Age", "Rate", "CILo", "CIHi"))

pcurve(DATA, col = "darkred", colf = "pink", lwd = 3, cex = 1.0, pch = 21)

title(main = "Longitudinal Age Curve", cex.main = 1)

DATA <- cbind(matrix(M$QuadCrossAge[,1]), (M$QuadCrossAge[,c(2,3,4)]))

dimnames(DATA) <- list(c(), c("Age", "Rate", "CILo", "CIHi"))

pcurve(DATA, lwd = 3, col = "darkred", colf = "pink", cex = 1.0, pch = 21)

title(main = "Cross-Sectional Age Curve", cex.main = 1)

pcurve(M$Long2CrossRR, lwd = 3, col = "darkred", colf = "pink", cex = 1.0, pch = 21)

abline(1,0, lty = 3)

title(main = "Long vs. Cross RR", cex.main = 1)

pcurve(M$QuadFittedTemporalTrends, col = "steelblue4", colf = "slategray1", lwd = 3, cex = 1.0, pch = 21)

title(main = "Fitted Temporal Trends", cex.main = 1)

pcurve(M$QuadPeriodRR, col = "steelblue4", colf = "slategray1", lwd = 3, cex = 1.0, pch = 21)

abline(1, 0, lty = 3)

title(main = "Period RR", cex.main = 1)

pcurve(M$QuadCohortRR, col = "seagreen4", colf = "darkseagreen1", lwd = 3, cex = 1.0, pch = 21)

abline(1, 0, lty = 3)

title(main = "Cohort RR", cex.main = 1)

pcurve(M$QuadLocalDrifts, col = "black", colf = "grey88", lwd = 3, cex = 1.0, pch = 21)

abline(M$NetDrift[1,1], 0, lty = 3)

title(main = "Local Drifts", cex.main = 1)

pcurve(M$QuadAgeDeviations, col = "darkred", colf = "pink", lwd = 3, cex = 1.0, pch = 21)

title(main = "Age Deviations", cex.main = 1)

pcurve(M$QuadPerDeviations, col = "steelblue4", colf = "slategray1", lwd = 3 , cex = 1.0, pch = 21)

abline(0, 0, lty = 3)

title(main = "Period Deviations", cex.main = 1)

pcurve(M$QuadCohDeviations, col = "seagreen4", colf = "darkseagreen1", lwd = 3, cex = 1.0, pch = 21)

abline(0, 0, lty = 3)

title(main = "Cohort Deviations", cex.main = 1)

pcurve(M$QuadFittedCohortPattern, col = "seagreen4", colf = "darkseagreen1", lwd = 3, cex = 1.0, pch = 21)

abline(0, 0, lty = 3)

title(main = "Fitted Cohort Pattern", cex.main = 1)

DATA <- cbind(matrix(M$QuadGradientShifts[,1]), (M$QuadGradientShifts[,c(2,3,4)]))

dimnames(DATA) <- list(c(), c("Period", "Mean % Change Per Year of Age", "CILo", "CIHi"))

pcurve(DATA, col = "darkred", colf = "pink", lwd = 3, cex = 1.0, pch = 21)

title(main = "Gradient Shifts", cex.main = 1)

abline(100*(exp(M$Coefficients[4,1]) - 1), 0, lty = 3)

}

pcurve <- function(DATA, col = 'steelblue4', colf = 'slategray1', bg = 'grey99', pch = 1, type = 'b', lty = 1, lwd = 2, XLim = NA, YLim = NA, cex = 1.5)

{

# Plot a selected output from an age-period-cohort model.

x <- DATA[,1]

rangex <- range(x)[2]-range(x)[1]

if (is.na(XLim[1])) {XLim <- c(min(x)-0.05*rangex, max(x)+0.05*rangex)}

xl <- dimnames(DATA)[[2]][1]

y <- DATA[,2]

rangey <- range(y)[2]-range(y)[1]

if (is.na(YLim[1])) {YLim <- c(min(y)-0.05*rangey, max(y)+0.05*rangey)}

yl <- dimnames(DATA)[[2]][2]

if (ncol(DATA)==4){

xci <- c(x, rev(x))

yci <- c(DATA[, 3], rev(DATA[, 4]))

rangey <- range(yci)[2]-range(yci)[1]

YLim <- c(min(yci) - 0.05*rangey, max(yci) + 0.05*rangey)}

else {yci <- NULL}

plot(x, y, col = col, pch = pch, type = type, lty = lty, lwd = lwd, cex = cex,

xlab = xl, ylab = yl, xlim = XLim, ylim = YLim, las = 1, bg = bg)

if (!is.null(yci[1])){

polygon(xci, yci, col = colf, border = colf)

}

points(x, y, col = col, pch = pch, type = type, lty = lty, lwd = lwd, cex = cex,

xlab = xl, ylab = yl, xlim = XLim, ylim = YLim, las = 1, bg = bg)

}

setwd("D:/年龄周期队列模型")

#install.packages("prepare_rates")

library(magrittr)

library(dplyr)

library(data.table)

source('source_apc.R')

source('function_year5.R')

#读取数据，查看数据结构

Global <- fread('Global.csv')

str(Global)

Global_infections<-subset(Global,

Global$cause_name =='HIV/AIDS and sexually transmitted infections'|

Global$cause_name =='Other infectious diseases')

unique(Global_infections$year)

#提取年龄分组

age1 <- c("<5 years","5-9 years","10-14 years","15-19 years","20-24 years",

"25-29 years","30-34 years","35-39 years","40-44 years","45-49 years",

"50-54 years","55-59 years","60-64 years","65-69 years","70-74 years",

"75-79 years","80-84 years","85-89 years","90-94 years","95+ years") ###20个年龄组

age2 <- c("<5 years","5-9 years","10-14 years","15-19 years","20-24 years",

"25-29 years","30-34 years","35-39 years","40-44 years","45-49 years",

"50-54 years","55-59 years","60-64 years","65-69 years","70-74 years",

"75-79 years","80-84 years","85-89 years","90-94 years","95+ years") ###20个年龄

####死亡人数的年龄周期队列####

#1.发生率的发生人数，数据提取

Global_infections_both<- subset(Global_infections,

(Global_infections$age_name %in% age1 ) &

Global_infections$sex_name=="Both"&

Global_infections$location_name=='Global'&

Global_infections$metric_name== 'Number' &

Global_infections$measure_name=='Deaths')

### 按年份和年龄合并传染病1+2数值

library(dplyr)

Global_combined <- Global_infections_both %>%

group_by(year = year, age = age_name) %>% # 请确认实际年份列名

summarise(

val = sum(val, na.rm = TRUE),

upper = sum(upper, na.rm = TRUE),

lower = sum(lower, na.rm = TRUE),

location_name = first(location_name),

sex_name = first(sex_name),

metric_name = first(metric_name),

measure_name = first(measure_name),

age_id = first(age_id),

cause_name = "Combined Total",

.groups = 'drop'

)

# 合并到原始数据（可选）

#Final_data <- bind_rows(Global_infections_both, Global_combined)

#查看年龄分组

unique(Global_combined$age)

#删减 years字样

Global_combined$age<-gsub(" years","",Global_combined$age)

unique(Global_combined$age)

#因子化

Global_combined$age <- factor(Global_combined$age, levels = c("<5", "5-9", "10-14", "15-19",

"20-24", "25-29", "30-34", "35-39", "40-44", "45-49", "50-54",

"55-59", "60-64", "65-69", "70-74", "75-79", "80-84", "85-89",

"90-94", "95+"))

#选择age_id age_name,year, val 四列

Global_combined <- Global_combined[,c("age_id","age","year","val")]

##解决因格式问题的报错

library(data.table)

# 将数据转换为data.table格式

setDT(Global_combined)

# 执行长转宽操作

Global_combined_n <- dcast(

data = Global_combined,

age_id + age ~ year, # 左侧为保持不变的列，右侧为展开的列

value.var = "val" # 明确指定数值列

)

#长转宽，年份作为列

#Global_combined_n <- dcast(data = Global_combined, age_id + age ~ year)

#转成5年一组, 从21年开始往前每5年一个组

Global_combined_g <- function_year5(Global_combined_n, 1990, 2021, 2021)

#列名

rownames(Global_combined_g) <- Global_combined_n$age

#####导入人口数据####

Global_population <- fread('Global_population.csv')

var_name <- c("location_name","sex_name","year","age_id","age_name","val")

#population <- data.frame()

#population<-data.frame()

#for(k in 1:length(fileName)){

# data = read.csv(file = paste(path,fileName[k],sep = "\\"),

# header = T,stringsAsFactors = F)

#population=rbind(population,data)

#}

Global_population<-Global_population%>% dplyr::select(var_name) %>%

filter(location_name %in% 'Global' & age_name %in% age2 & sex_name %in% 'Both')

#去掉age_name的字样

Global_population$age_name<-gsub(" years","",Global_population$age_name)

#选择age_id age_name,year, val 四列

Global_population <- Global_population[,c("age_id","age_name","year","val")]

#人口数据的长转宽

Global_population_n <- dcast(data = Global_population, age_id + age_name ~ year)

#转成五年一组

Global_population_g <- function_year5(Global_population_n, 1990, 2021, 2021)

rownames(Global_population_g) <- Global_population_n$age_name

######查看两个数据的年龄段是否有区别####

#取两个数据集的交集

name <- intersect(Global_population_n$age_name,Global_combined_n$age)

#提取不同年龄段数据

Global_population_g <- Global_population_g[rownames(Global_population_g) %in% name,]

Global_combined_g <- Global_combined_g[rownames(Global_combined_g) %in% name,]

####按数据所需格式排列结果数据

name2 <- paste0(names(Global_population_g),"p")

Global_population_g <- Global_population_g %>% stats::setNames(name2)

both_population <- tibble(cbind(Global_combined_g,Global_population_g)) %>%

dplyr::select(`1990-1991`,`1990-1991p`,`1992-1996`,`1992-1996p`,`1997-2001`,`1997-2001p`,

`2002-2006`,`2002-2006p`,`2007-2011`,`2007-2011p`,`2012-2016`,`2012-2016p`,

`2017-2021`,`2017-2021p`)

write.table(both_population,'both_population.csv',row.names = F,col.names = F,sep = ',')

###R中画图#####

## APC模型进一步处理

R <- prepare_rates(both_population,

StartYear=1990,StartAge=15,Interval=5,

fullname='',description='')

## APC模型计算

M <- apc2fit(R)

## 画图

plot.apc1(M)

######DALYs######

unique(Global_infections$measure_name)

#1.发生率的发生人数，数据提取

Global_infections_both2<- subset(Global_infections,

(Global_infections$age_name %in% age1 ) &

Global_infections$sex_name=="Both"&

Global_infections$location_name=='Global'&

Global_infections$metric_name== 'Number' &

Global_infections$measure_name=='DALYs (Disability-Adjusted Life Years)')

### 按年份和年龄合并传染病1+2数值

library(dplyr)

Global_combined2 <- Global_infections_both2 %>%

group_by(year = year, age = age_name) %>% # 请确认实际年份列名

summarise(

val = sum(val, na.rm = TRUE),

upper = sum(upper, na.rm = TRUE),

lower = sum(lower, na.rm = TRUE),

location_name = first(location_name),

sex_name = first(sex_name),

metric_name = first(metric_name),

measure_name = first(measure_name),

age_id = first(age_id),

cause_name = "Combined Total",

.groups = 'drop'

)

# 合并到原始数据（可选）

#Final_data <- bind_rows(Global_infections_both, Global_combined)

#查看年龄分组

unique(Global_combined2$age)

#删减 years字样

Global_combined2$age<-gsub(" years","",Global_combined2$age)

unique(Global_combined2$age)

#因子化

Global_combined2$age <- factor(Global_combined2$age, levels = c("<5", "5-9", "10-14", "15-19",

"20-24", "25-29", "30-34", "35-39", "40-44", "45-49", "50-54",

"55-59", "60-64", "65-69", "70-74", "75-79", "80-84", "85-89",

"90-94", "95+"))

#选择age_id age_name,year, val 四列

Global_combined2 <- Global_combined2[,c("age_id","age","year","val")]

##解决因格式问题的报错

library(data.table)

# 将数据转换为data.table格式

setDT(Global_combined2)

# 执行长转宽操作

Global_combined_n2 <- dcast(

data = Global_combined2,

age_id + age ~ year, # 左侧为保持不变的列，右侧为展开的列

value.var = "val" # 明确指定数值列

)

#长转宽，年份作为列

#Global_combined_n2 <- dcast(data = Global_combined2, age_id + age ~ year)

#转成5年一组, 从21年开始往前每5年一个组

Global_combined_g2 <- function_year5(Global_combined_n2, 1990, 2021, 2021)

#列名

rownames(Global_combined_g2) <- Global_combined_n2$age

#####导入人口数据####

Global_population <- fread('Global_population.csv')

var_name <- c("location_name","sex_name","year","age_id","age_name","val")

#population <- data.frame()

#population<-data.frame()

#for(k in 1:length(fileName)){

# data = read.csv(file = paste(path,fileName[k],sep = "\\"),

#header = T,stringsAsFactors = F)

# population=rbind(population,data)

#}

Global_population<-Global_population%>% dplyr::select(var_name) %>%

filter(location_name %in% 'Global' & age_name %in% age2 & sex_name %in% 'Both')

#去掉age_name的字样

Global_population$age_name<-gsub(" years","",Global_population$age_name)

#选择age_id age_name,year, val 四列

Global_population <- Global_population[,c("age_id","age_name","year","val")]

#人口数据的长转宽

Global_population_n <- dcast(data = Global_population, age_id + age_name ~ year)

#转成五年一组

Global_population_g <- function_year5(Global_population_n, 1990, 2021, 2021)

rownames(Global_population_g) <- Global_population_n$age_name

######查看两个数据的年龄段是否有区别####

#取两个数据集的交集

name <- intersect(Global_population_n$age_name,Global_combined_n2$age)

#提取不同年龄段数据

Global_population_g <- Global_population_g[rownames(Global_population_g) %in% name,]

Global_combined_g2 <- Global_combined_g2[rownames(Global_combined_g2) %in% name,]

####按数据所需格式排列结果数据

name2 <- paste0(names(Global_population_g),"p")

Global_population_g <- Global_population_g %>% stats::setNames(name2)

both_population2 <- tibble(cbind(Global_combined_g2,Global_population_g)) %>%

dplyr::select(`1990-1991`,`1990-1991p`,`1992-1996`,`1992-1996p`,`1997-2001`,`1997-2001p`,

`2002-2006`,`2002-2006p`,`2007-2011`,`2007-2011p`,`2012-2016`,`2012-2016p`,

`2017-2021`,`2017-2021p`)

write.table(both_population2,'both_populationDALYs.csv',row.names = F,col.names = F,sep = ',')

both_population2 <- fread('both_populationDALYs.csv')

###R中画图#####

## APC模型进一步处理

R <- prepare_rates(both_population2,

StartYear=1990,StartAge=15,Interval=5,

fullname='',description='')

## APC模型计算

M <- apc2fit(R)

## 画图

plot.apc2(M)

**PART 4 Data Merge**

#设置工作空间

setwd("D:\\数据整理")

###数据在另一个文件夹中,给他设定一个别的路径####

path = "数据"

fileName = dir(path)

fileName

newdata<-data.frame()

for(k in 1:length(fileName)){

data = read.csv(file = paste0("数据/",fileName[k]),

header = T,stringsAsFactors = F)

newdata=rbind(newdata,data)

}

#write.csv(newdata,"total.csv")

unique(newdata$location_name)###查看newdata数据中的location_name的内容,同时unique()会进行去重

####中国数据：

IBD_china<-subset(newdata,newdata$location_name == 'China') ###挑选子集

write.csv(IBD_china,"IBD_china.csv")

####7个超级地区+全球数据

IBD_super_region <-subset(newdata,(newdata$location_name == 'Central Europe, Eastern Europe, and Central Asia'|

newdata$location_name == 'High-income'|

newdata$location_name == 'Latin America and Caribbean'|

newdata$location_name == 'North Africa and Middle East'|

newdata$location_name == 'South Asia'|

newdata$location_name == 'Southeast Asia, East Asia, and Oceania'|

newdata$location_name == 'Sub-Saharan Africa'|

newdata$location_name == 'Global' ))

unique(IBD_super_region$location_name)###检查

write.csv(IBD_super_region,"IBD_super_region.csv")

#####21个地区

IBD_region <-subset(newdata,(newdata$location_name == 'High-income Asia Pacific'|

newdata$location_name == 'Central Asia'|

newdata$location_name == 'Southeast Asia'|

newdata$location_name == 'East Asia'|

newdata$location_name == 'Central Europe'|

newdata$location_name == 'Eastern Europe'|

newdata$location_name == 'North Africa and Middle East'|

newdata$location_name == 'Australasia'|

newdata$location_name == 'Western Europe'|

newdata$location_name == 'Andean Latin America'|

newdata$location_name == 'Caribbean'|

newdata$location_name == 'High-income North America'|

newdata$location_name == 'Western Sub-Saharan Africa'|

newdata$location_name == 'South Asia'|

newdata$location_name == 'Oceania'|

newdata$location_name == 'Central Sub-Saharan Africa'|

newdata$location_name == 'Central Latin America'|

newdata$location_name == 'Southern Latin America'|

newdata$location_name == 'Tropical Latin America'|

newdata$location_name == 'Eastern Sub-Saharan Africa'|

newdata$location_name == 'Southern Sub-Saharan Africa'

))

unique(IBD_region$location_name)###检查

write.csv(IBD_region,"IBD_region.csv")

####204个国家

IBD_country <-subset(newdata,(newdata$location_name != 'Central Europe, Eastern Europe, and Central Asia'&

newdata$location_name != 'High-income'&

newdata$location_name != 'Latin America and Caribbean'&

newdata$location_name != 'North Africa and Middle East'&

newdata$location_name != 'South Asia'&

newdata$location_name != 'Southeast Asia, East Asia, and Oceania'&

newdata$location_name != 'Sub-Saharan Africa'&

newdata$location_name != 'Global'&

newdata$location_name != 'High-income Asia Pacific'&

newdata$location_name != 'Central Asia'&

newdata$location_name != 'Southeast Asia'&

newdata$location_name != 'East Asia'&

newdata$location_name != 'Central Europe'&

newdata$location_name != 'Eastern Europe'&

newdata$location_name != 'North Africa and Middle East'&

newdata$location_name != 'Australasia'&

newdata$location_name != 'Western Europe'&

newdata$location_name != 'Andean Latin America'&

newdata$location_name != 'Caribbean'&

newdata$location_name != 'High-income North America'&

newdata$location_name != 'Western Sub-Saharan Africa'&

newdata$location_name != 'South Asia'&

newdata$location_name != 'Oceania'&

newdata$location_name != 'Central Sub-Saharan Africa'&

newdata$location_name != 'Central Latin America'&

newdata$location_name != 'Southern Latin America'&

newdata$location_name != 'Tropical Latin America'&

newdata$location_name != 'Eastern Sub-Saharan Africa'&

newdata$location_name != 'Southern Sub-Saharan Africa'))

unique(IBD_country$location_name)###检查

write.csv(IBD_country,"IBD_country.csv")

####5个SDI地区

IBD_SDI_region <-subset(newdata,(newdata$location_name == 'Low SDI'|

newdata$location_name == 'High-middle SDI'|

newdata$location_name == 'High SDI'|

newdata$location_name == 'Low-middle SDI'|

newdata$location_name == 'Middle SDI'))

unique(IBD_SDI_region$location_name)###检查

write.csv(IBD_SDI_region,"IBD_SDI_region.csv")

####5个SDI地区+Global

SDI_Global <-subset(newdata,(newdata$location_name == 'Low SDI'|

newdata$location_name == 'High-middle SDI'|

newdata$location_name == 'High SDI'|

newdata$location_name == 'Low-middle SDI'|

newdata$location_name == 'Middle SDI'|

newdata$location_name == 'Global' ))

unique(SDI_Global$location_name)###检查

write.csv(SDI_Global,"SDI_Global.csv")

**PART 5 joinpoint**

#setwd("D:/Joinpoint ")

Global <- read.csv('Global.csv',header = T) ###读取数据

####第一步 接下来论文中使用Joinpoint回归分析了年龄标准化发病率与年龄标准化死亡率####

###全球 传染1 死亡

ASIR<- subset(Global,Global$measure_name=='Deaths'& ###选择死亡

(Global$age_name == "Age-standardized") & ###年龄标准化

Global$metric_name== 'Rate') ###选择率

unique(ASIR$cause_name)

ASIR_infections1<-subset(ASIR,

ASIR$cause_name =='HIV/AIDS and sexually transmitted infections')

unique(ASIR_infections1$measure_name)

#计算发病率的标准误

ASIR_infections1$SE <- (ASIR_infections1$upper-ASIR_infections1$lower)/(1.96*2) ##根据95%UI 计算标准误

str(ASIR_infections1)

##获取性别、年份以及对应的数值和SE的值

ASIR_infections1 <- ASIR_infections1[,c("sex_name","year","val","SE")]

# joinpoint要求年份按照升序排列，且一个组放在一起

ASIR_infections1 <- ASIR_infections1[order(ASIR_infections1$sex_name,ASIR_infections1$year),]

write.csv(ASIR_infections1,'ASIR_infections1_Deaths_joinpoint.csv') ###输出数据为CSV

####joinpoint上打开Incidence_joinpoint.csv进行回归分析####

####第二步 调整发病率及可信区间的表示形式

# 数据处理好后,继续用R处理

AAPC <- read.table('export.Export.AAPC.txt',header = T) ##读取AAPC的数据

names(AAPC)[6:8] <- c('val','lower','upper')###更改第2到第4列的表头名字

#保留两个小数点

AAPC$val <- sprintf("%0.2f",AAPC$val) ##小数位数

AAPC$lower <- sprintf("%0.2f",AAPC$lower) ##小数位数

AAPC$upper <- sprintf("%0.2f",AAPC$upper) ##小数位数

#

AAPC$AAPC <- paste0(AAPC$val," (",AAPC$lower," - ",AAPC$upper,")")

write.csv(AAPC,'deaths1_AAPC.csv')

#读取APC数据

APC <- read.table('export.Export.APC.txt',header = T)

names(APC)[6:8] <- c('val','lower','upper')

#保留两个小数点

APC$val <- sprintf("%0.2f",APC$val) ##小数位数

APC$lower <- sprintf("%0.2f",APC$lower) ##小数位数

APC$upper <- sprintf("%0.2f",APC$upper) ##小数位数

APC$APC <- paste0(APC$val," (",APC$lower," - ",APC$upper,")")

write.csv(APC,'deaths1_APC.csv')

###全球 传染2 死亡-----------------------------------------------------

ASIR<- subset(Global,Global$measure_name=='Deaths'& ###选择死亡

(Global$age_name == "Age-standardized") & ###年龄标准化

Global$metric_name== 'Rate') ###选择率

unique(ASIR$cause_name)

ASIR_infections2<-subset(ASIR,

ASIR$cause_name =='Other infectious diseases')

unique(ASIR_infections2$measure_name)

#计算发病率的标准误

ASIR_infections2$SE <- (ASIR_infections2$upper-ASIR_infections2$lower)/(1.96*2) ##根据95%UI 计算标准误

str(ASIR_infections2)

##获取性别、年份以及对应的数值和SE的值

ASIR_infections2 <- ASIR_infections2[,c("sex_name","year","val","SE")]

# joinpoint要求年份按照升序排列，且一个组放在一起

ASIR_infections2 <- ASIR_infections2[order(ASIR_infections2$sex_name,ASIR_infections2$year),]

write.csv(ASIR_infections2,'ASIR_infections2_Deaths_joinpoint.csv') ###输出数据为CSV

####joinpoint上打开Incidence_joinpoint.csv进行回归分析####

###全球 传染1 DALYs-----------------------------------------------------

unique(Global$measure_name)

ASIR<- subset(Global,Global$measure_name=='DALYs (Disability-Adjusted Life Years)'& ###选择DALYs (Disability-Adjusted Life Years)

(Global$age_name == "Age-standardized") & ###年龄标准化

Global$metric_name== 'Rate') ###选择率

unique(ASIR$cause_name)

ASIR_infections1<-subset(ASIR,

ASIR$cause_name =='HIV/AIDS and sexually transmitted infections')

unique(ASIR_infections1$measure_name)

#计算发病率的标准误

ASIR_infections1$SE <- (ASIR_infections1$upper-ASIR_infections1$lower)/(1.96*2) ##根据95%UI 计算标准误

str(ASIR_infections1)

##获取性别、年份以及对应的数值和SE的值

ASIR_infections1 <- ASIR_infections1[,c("sex_name","year","val","SE")]

# joinpoint要求年份按照升序排列，且一个组放在一起

ASIR_infections1 <- ASIR_infections1[order(ASIR_infections1$sex_name,ASIR_infections1$year),]

write.csv(ASIR_infections1,'ASIR_infections1_DALYs_joinpoint.csv') ###输出数据为CSV

####joinpoint上打开Incidence_joinpoint.csv进行回归分析####

###全球 传染2 DALYs-----------------------------------------------------

unique(Global$measure_name)

ASIR<- subset(Global,Global$measure_name=='DALYs (Disability-Adjusted Life Years)'& ###选择DALYs (Disability-Adjusted Life Years)

(Global$age_name == "Age-standardized") & ###年龄标准化

Global$metric_name== 'Rate') ###选择率

unique(ASIR$cause_name)

ASIR_infections2<-subset(ASIR,

ASIR$cause_name =='Other infectious diseases')

unique(ASIR_infections2$measure_name)

#计算发病率的标准误

ASIR_infections2$SE <- (ASIR_infections2$upper-ASIR_infections2$lower)/(1.96*2) ##根据95%UI 计算标准误

str(ASIR_infections2)

##获取性别、年份以及对应的数值和SE的值

ASIR_infections2 <- ASIR_infections2[,c("sex_name","year","val","SE")]

# joinpoint要求年份按照升序排列，且一个组放在一起

ASIR_infections2 <- ASIR_infections2[order(ASIR_infections2$sex_name,ASIR_infections2$year),]

write.csv(ASIR_infections2,'ASIR_infections2_DALYs_joinpoint.csv') ###输出数据为CSV

####joinpoint上打开Incidence_joinpoint.csv进行回归分析####

**PART 6 GBD map**

#设置工作空间

setwd("D:/GBD map")

install.packages('ggmap')

install.packages('maps')

# install.packages('dplyr')

#install.packages('countrycode')

library(ggmap)

library(maps)

library(dplyr)

library(RColorBrewer)

IBD<- vroom::vroom("D: \\IBD_country\\IBD_country.csv")

#2021年 Age-standardized DALYs rates

CIR_2021 <- read.csv("204countryDALYs.csv",header = T,check.names = F)

unique(CIR_2021$cause_name)

CIR_2021.1 <- subset(

CIR_2021, # 数据框

cause_name == 'HIV/AIDS and sexually transmitted infections' # 筛选条件

)

CIR_2021.1 <- subset(CIR_2021.1,CIR_2021.1$year==2021 &

CIR_2021.1$age_name=='All ages' &

CIR_2021.1$metric_name== 'Rate' &

CIR_2021.1$measure_name=='DALYs (Disability-Adjusted Life Years)'&

CIR_2021.1$sex_name=='Both')

CIR_2021.1 <- CIR_2021.1[,c(4,16,17,18)] ###选择位置与数据+95%UI

CIR_2021.1$val <- round(CIR_2021.1$val,2) ###率保留2位小数点

CIR_2021.1$lower <- round(CIR_2021.1$lower,2)

CIR_2021.1$upper <- round(CIR_2021.1$upper,2)

#### map for ASMR

worldData <- map_data('world') #####使用map包提取世界·范围内的地图

country_asr <- CIR_2021.1 ####

country_asr$location <- as.character(country_asr$location_name)

###以下代码的目的是让country_asr$location的国家名称与worldData的国家名称一致

### 这样才能让数据映射到地图上

country_asr$location[country_asr$location == 'United States of America'] = 'USA'

country_asr$location[country_asr$location == 'Russian Federation'] = 'Russia'

country_asr$location[country_asr$location == 'United Kingdom'] = 'UK'

country_asr$location[country_asr$location == 'Congo'] = 'Republic of Congo'

country_asr$location[country_asr$location == "Iran (Islamic Republic of)"] = 'Iran'

country_asr$location[country_asr$location == "Democratic People's Republic of Korea"] = 'North Korea'

country_asr$location[country_asr$location == "Taiwan (Province of China)"] = 'Taiwan'

country_asr$location[country_asr$location == "Republic of Korea"] = 'South Korea'

country_asr$location[country_asr$location == "United Republic of Tanzania"] = 'Tanzania'

country_asr$location[country_asr$location == "C?te d'Ivoire"] = 'Saint Helena'

country_asr$location[country_asr$location == "Bolivia (Plurinational State of)"] = 'Bolivia'

country_asr$location[country_asr$location == "Venezuela (Bolivarian Republic of)"] = 'Venezuela'

country_asr$location[country_asr$location == "Czechia"] = 'Czech Republic'

country_asr$location[country_asr$location == "Republic of Moldova"] = 'Moldova'

country_asr$location[country_asr$location == "Viet Nam"] = 'Vietnam'

country_asr$location[country_asr$location == "Lao People's Democratic Republic"] = 'Laos'

country_asr$location[country_asr$location == "Syrian Arab Republic"] = 'Syria'

country_asr$location[country_asr$location == "North Macedonia"] = 'Macedonia'

country_asr$location[country_asr$location == "Micronesia (Federated States of)"] = 'Micronesia'

country_asr$location[country_asr$location == "Macedonia"] = 'North Macedonia'

country_asr$location[country_asr$location == "Trinidad and Tobago"] = 'Trinidad'

country_asr <- rbind(country_asr,country_asr[country_asr$location == "Trinidad",])

country_asr$location[country_asr$location == "Trinidad"] = 'Tobago'

country_asr$location[country_asr$location == "Cabo Verde"] = 'Cape Verde'

country_asr$location[country_asr$location == "United States Virgin Islands"] = 'Virgin Islands'

country_asr$location[country_asr$location == "Antigua and Barbuda"] = 'Antigu'

country_asr <- rbind(country_asr,country_asr[country_asr$location == "Antigu",])

country_asr$location[country_asr$location == "Antigu"] = 'Barbuda'

country_asr$location[country_asr$location == "Saint Kitts and Nevis"] = 'Saint Kitts'

country_asr <- rbind(country_asr,country_asr[country_asr$location == "Saint Kitts",])

country_asr$location[country_asr$location == "Saint Kitts"] = 'Nevis'

country_asr$location[country_asr$location == "Côte d'Ivoire"] = 'Ivory Coast'

country_asr$location[country_asr$location == "Saint Vincent and the Grenadines"] = 'Saint Vincent'

country_asr <- rbind(country_asr,country_asr[country_asr$location == "Saint Vincent",])

country_asr$location[country_asr$location == "Saint Vincent"] = 'Grenadines'

country_asr$location[country_asr$location == "Eswatini"] = 'Swaziland'

country_asr$location[country_asr$location == "Brunei Darussalam"] = 'Brunei'

country_asr$location[country_asr$location == "Türkiye"] = 'Turkey'

unique(country_asr$location_name)

#write.csv(country_asr,"ASDR_204.1.csv")

total <- full_join(worldData,country_asr,by = c('region'='location'))##把两个数据根据地点合并起来

mycolor2<-rev(brewer.pal(8, "Spectral") ) ##选择我们觉得好看的颜色，这里使用了brewer.pal

summary(total$val)

quantile(total$val,seq(0.1,1,0.1),na.rm = T)

total <- total %>% mutate(val2 = cut(val, breaks = c(0.00,2.25,7.00,8.45,14.77,25.47,67.32,215.66,398.68),

labels = c("<2.25",

"2.25~7.00",

"7.00~8.45",

"8.45~14.77",

"14.77~25.47",

"25.47~67.32",

"67.32~215.66",

"215.66~398.68"), ## breaks需要根据自己的实际结果来调整

include.lowest = T,right = T))

total1<-na.omit(total)

p <- ggplot()

p1 <- p + geom_polygon(data=total,

aes(x=long, y=lat, group = group,fill=val2),

colour="gray",size = .01) +

scale_fill_manual(values = mycolor2) +

theme_void()+labs(x="", y="")+

guides(fill = guide_legend(title='ASDR(/10^5)'))+

theme(legend.position = 'right')

p1

###修改后

library(countrycode)

# 将 country_asr 的 location 转换为 ISO3C 代码（标准化中间步骤）

country_asr$iso3c <- countrycode(

sourcevar = country_asr$location_name,

origin = "country.name",

destination = "iso3c"

)

# 从 ISO3C 代码转回地图数据中的通用名称

country_asr$location <- countrycode(

sourcevar = country_asr$iso3c,

origin = "iso3c",

destination = "country.name"

)

# 特殊处理个别名称（如 USA）

country_asr$location[country_asr$location_name == "United States of America"] <- "USA"

#查找未匹配国家

world_regions <- unique(worldData$region)

country_asr_unmatched <- country_asr[!country_asr$location %in% world_regions, ]

print(country_asr_unmatched$location)

# 手动修正特殊名称

country_asr$location <- case_when(

country_asr$location_name == "People's Republic of China" ~ "China",

country_asr$location_name == "Democratic Republic of the Congo" ~ "DR Congo",

country_asr$location_name == "Russian Federation" ~ "Russia",

TRUE ~ country_asr$location

)

# 手动修正未匹配的国家名称

country_asr <- country_asr %>%

mutate(location = case_when(

# 逐个修正

location == "Myanmar (Burma)" ~ "Myanmar",

location == "Micronesia (Federated States of)" ~ "Micronesia",

location == "Bosnia & Herzegovina" ~ "Bosnia and Herzegovina",

location == "Czechia" ~ "Czech Republic",

location == "United Kingdom" ~ "UK",

location == "Antigua & Barbuda" ~ "Antigua",

location == "St. Vincent & Grenadines" ~ "Saint Vincent and the Grenadines",

location == "St. Lucia" ~ "Saint Lucia",

location == "Trinidad & Tobago" ~ "Trinidad and Tobago",

location == "Palestinian Territories" ~ "Palestine",

location == "DR Congo" ~ "Democratic Republic of the Congo",

location == "Congo - Brazzaville" ~ "Republic of Congo",

location == "Eswatini" ~ "Swaziland", # 地图数据中仍可能用旧名

location == "Côte d’Ivoire" ~ "Ivory Coast",

location == "São Tomé & Príncipe" ~ "Sao Tome and Principe",

location == "St. Kitts & Nevis" ~ "Saint Kitts",

location == "U.S. Virgin Islands" ~ "Virgin Islands",

location == "Tokelau" ~ "Tokelau", # 地图数据可能不包含该区域

location == "Tuvalu" ~ "Tuvalu", # 同上

location == "Antigua & Barbuda" ~ "Antigua", # 重复项需统一

TRUE ~ location # 其他名称保持不变

))

# 再次检查未匹配国家（应减少至接近零）

world_regions <- unique(worldData$region)

country_asr_unmatched <- country_asr[!country_asr$location %in% world_regions, ]

print(unique(country_asr_unmatched$location))

# 手动修正未匹配的国家名称（针对灰色国家）

country_asr <- country_asr %>%

mutate(location = case_when(

# 修正用户列出的灰色国家名称

location == "Australia" ~ "Australia", # 确保名称一致

location == "New Zealand" ~ "New Zealand",

location == "Japan" ~ "Japan",

location == "Republic of Korea" ~ "South Korea",

location == "Democratic Socialist Republic of Sri Lanka" ~ "Sri Lanka",

location == "Republic of Yemen" ~ "Yemen",

location == "Islamic Republic of Mauritania" ~ "Mauritania",

location == "Plurinational State of Bolivia" ~ "Bolivia",

location == "Republic of Peru" ~ "Peru",

location == "Co-operative Republic of Guyana" ~ "Guyana",

location == "Republic of Guatemala" ~ "Guatemala",

location == "Portuguese Republic" ~ "Portugal",

location == "Türkiye" ~ "Türkiye", # 若地图数据使用旧名称

location == "Syrian Arab Republic" ~ "Syria",

location == "Republic of Albania" ~ "Albania",

location == "North Macedonia" ~ "North Macedonia", # 确保地图数据支持此名称

location == "Republic of Montenegro" ~ "Montenegro",

location == "Bosnia and Herzegovina" ~ "Bosnia and Herzegovina", # 确认拼写一致

location == "Hungary" ~ "Hungary",

location == "Republic of Slovenia" ~ "Slovenia",

location == "Slovak Republic" ~ "Slovakia",

location == "Czechia" ~ "Czech Republic",

location == "Kingdom of the Netherlands" ~ "Netherlands",

location == "Republic of Finland" ~ "Finland",

location == "Republic of Fiji" ~ "Fiji",

# 处理其他未匹配的 NA 和特殊地区

is.na(location) ~ NA_character_, # 保留 NA 但后续过滤

location == "Tokelau" ~ "Tokelau", # 若地图数据不包含，需过滤

location == "Tuvalu" ~ "Tuvalu", # 同上

TRUE ~ location

)) %>%

filter(!is.na(location)) # 移除 NA 值

# 特殊处理：若地图数据使用 "Swaziland" 而非 "Eswatini"

country_asr$location[country_asr$location == "Eswatini"] <- "Swaziland"

# 处理地图数据中不包含的小区域（如 Tokelau）

country_asr <- country_asr %>%

filter(!location %in% c("Tokelau", "Tuvalu"))

# 再次检查未匹配国家

world_regions <- unique(worldData$region)

country_asr_unmatched <- country_asr[!country_asr$location %in% world_regions, ]

print(unique(country_asr_unmatched$location)) # 应返回空或仅无关区域

anti_join(worldData, country_asr, by = c("region" = "location")) %>% distinct(region)

#######infections2----------------------------------------------------

CIR_2021.2 <- subset(

CIR_2021, # 数据框

cause_name == 'Other infectious diseases' # 筛选条件

)

CIR_2021.2 <- subset(CIR_2021.2,CIR_2021.2$year==2021 &

CIR_2021.2$age_name=='All ages' &

CIR_2021.2$metric_name== 'Rate' &

CIR_2021.2$measure_name=='DALYs (Disability-Adjusted Life Years)'&

CIR_2021.2$sex_name=='Both')

CIR_2021.2 <- CIR_2021.2[,c(4,16,17,18)] ###选择位置与数据+95%UI

CIR_2021.1$val <- round(CIR_2021.1$val,2) ###率保留2位小数点

CIR_2021.1$lower <- round(CIR_2021.1$lower,2)

CIR_2021.1$upper <- round(CIR_2021.1$upper,2)

#### map for ASMR

worldData <- map_data('world') #####使用map包提取世界·范围内的地图

country_asr <- CIR_2021.2 ####

country_asr$location <- as.character(country_asr$location_name)

###以下代码的目的是让country_asr$location的国家名称与worldData的国家名称一致

### 这样才能让数据映射到地图上

country_asr$location[country_asr$location == 'United States of America'] = 'USA'

country_asr$location[country_asr$location == 'Russian Federation'] = 'Russia'

country_asr$location[country_asr$location == 'United Kingdom'] = 'UK'

country_asr$location[country_asr$location == 'Congo'] = 'Republic of Congo'

country_asr$location[country_asr$location == "Iran (Islamic Republic of)"] = 'Iran'

country_asr$location[country_asr$location == "Democratic People's Republic of Korea"] = 'North Korea'

country_asr$location[country_asr$location == "Taiwan (Province of China)"] = 'Taiwan'

country_asr$location[country_asr$location == "Republic of Korea"] = 'South Korea'

country_asr$location[country_asr$location == "United Republic of Tanzania"] = 'Tanzania'

country_asr$location[country_asr$location == "C?te d'Ivoire"] = 'Saint Helena'

country_asr$location[country_asr$location == "Bolivia (Plurinational State of)"] = 'Bolivia'

country_asr$location[country_asr$location == "Venezuela (Bolivarian Republic of)"] = 'Venezuela'

country_asr$location[country_asr$location == "Czechia"] = 'Czech Republic'

country_asr$location[country_asr$location == "Republic of Moldova"] = 'Moldova'

country_asr$location[country_asr$location == "Viet Nam"] = 'Vietnam'

country_asr$location[country_asr$location == "Lao People's Democratic Republic"] = 'Laos'

country_asr$location[country_asr$location == "Syrian Arab Republic"] = 'Syria'

country_asr$location[country_asr$location == "North Macedonia"] = 'Macedonia'

country_asr$location[country_asr$location == "Micronesia (Federated States of)"] = 'Micronesia'

country_asr$location[country_asr$location == "Macedonia"] = 'North Macedonia'

country_asr$location[country_asr$location == "Trinidad and Tobago"] = 'Trinidad'

country_asr <- rbind(country_asr,country_asr[country_asr$location == "Trinidad",])

country_asr$location[country_asr$location == "Trinidad"] = 'Tobago'

country_asr$location[country_asr$location == "Cabo Verde"] = 'Cape Verde'

country_asr$location[country_asr$location == "United States Virgin Islands"] = 'Virgin Islands'

country_asr$location[country_asr$location == "Antigua and Barbuda"] = 'Antigu'

country_asr <- rbind(country_asr,country_asr[country_asr$location == "Antigu",])

country_asr$location[country_asr$location == "Antigu"] = 'Barbuda'

country_asr$location[country_asr$location == "Saint Kitts and Nevis"] = 'Saint Kitts'

country_asr <- rbind(country_asr,country_asr[country_asr$location == "Saint Kitts",])

country_asr$location[country_asr$location == "Saint Kitts"] = 'Nevis'

country_asr$location[country_asr$location == "Côte d'Ivoire"] = 'Ivory Coast'

country_asr$location[country_asr$location == "Saint Vincent and the Grenadines"] = 'Saint Vincent'

country_asr <- rbind(country_asr,country_asr[country_asr$location == "Saint Vincent",])

country_asr$location[country_asr$location == "Saint Vincent"] = 'Grenadines'

country_asr$location[country_asr$location == "Eswatini"] = 'Swaziland'

country_asr$location[country_asr$location == "Brunei Darussalam"] = 'Brunei'

country_asr$location[country_asr$location == "Türkiye"] = 'Turkey'

unique(country_asr$location_name)

#write.csv(country_asr,"ASDR_204.1.csv")

total <- full_join(worldData,country_asr,by = c('region'='location'))##把两个数据根据地点合并起来

mycolor2<-rev(brewer.pal(6, "Spectral") ) ##选择我们觉得好看的颜色，这里使用了brewer.pal

summary(total$val)

quantile(total$val,seq(0.1,1,0.1),na.rm = T)

total <- total %>% mutate(val2 = cut(val, breaks = c(0.00,0.15,0.22,0.34,0.53,1.34,10.19),

labels = c("<0.15",

"0.15~0.22",

"0.22~0.34",

"0.34~0.53",

"0.53~1.34",

"1.34~10.19"

), ## breaks需要根据自己的实际结果来调整

include.lowest = T,right = T))

total1<-na.omit(total)

p <- ggplot()

p1.2 <- p + geom_polygon(data=total,

aes(x=long, y=lat, group = group,fill=val2),

colour="gray",size = .01) +

scale_fill_manual(values = mycolor2) +

theme_void()+labs(x="", y="")+

guides(fill = guide_legend(title='ASDR(/10^5)'))+

theme(legend.position = 'right')

p1.2

###修改后

library(countrycode)

# 将 country_asr 的 location 转换为 ISO3C 代码（标准化中间步骤）

country_asr$iso3c <- countrycode(

sourcevar = country_asr$location_name,

origin = "country.name",

destination = "iso3c"

)

# 从 ISO3C 代码转回地图数据中的通用名称

country_asr$location <- countrycode(

sourcevar = country_asr$iso3c,

origin = "iso3c",

destination = "country.name"

)

# 特殊处理个别名称（如 USA）

country_asr$location[country_asr$location_name == "United States of America"] <- "USA"

#查找未匹配国家

world_regions <- unique(worldData$region)

country_asr_unmatched <- country_asr[!country_asr$location %in% world_regions, ]

print(country_asr_unmatched$location)

# 手动修正特殊名称

country_asr$location <- case_when(

country_asr$location_name == "People's Republic of China" ~ "China",

country_asr$location_name == "Democratic Republic of the Congo" ~ "DR Congo",

country_asr$location_name == "Russian Federation" ~ "Russia",

TRUE ~ country_asr$location

)

# 手动修正未匹配的国家名称

country_asr <- country_asr %>%

mutate(location = case_when(

# 逐个修正

location == "Myanmar (Burma)" ~ "Myanmar",

location == "Micronesia (Federated States of)" ~ "Micronesia",

location == "Bosnia & Herzegovina" ~ "Bosnia and Herzegovina",

location == "Czechia" ~ "Czech Republic",

location == "United Kingdom" ~ "UK",

location == "Antigua & Barbuda" ~ "Antigua",

location == "St. Vincent & Grenadines" ~ "Saint Vincent and the Grenadines",

location == "St. Lucia" ~ "Saint Lucia",

location == "Trinidad & Tobago" ~ "Trinidad and Tobago",

location == "Palestinian Territories" ~ "Palestine",

location == "DR Congo" ~ "Democratic Republic of the Congo",

location == "Congo - Brazzaville" ~ "Republic of Congo",

location == "Eswatini" ~ "Swaziland", # 地图数据中仍可能用旧名

location == "Côte d’Ivoire" ~ "Ivory Coast",

location == "São Tomé & Príncipe" ~ "Sao Tome and Principe",

location == "St. Kitts & Nevis" ~ "Saint Kitts",

location == "U.S. Virgin Islands" ~ "Virgin Islands",

location == "Tokelau" ~ "Tokelau", # 地图数据可能不包含该区域

location == "Tuvalu" ~ "Tuvalu", # 同上

location == "Antigua & Barbuda" ~ "Antigua", # 重复项需统一

TRUE ~ location # 其他名称保持不变

))

# 再次检查未匹配国家（应减少至接近零）

world_regions <- unique(worldData$region)

country_asr_unmatched <- country_asr[!country_asr$location %in% world_regions, ]

print(unique(country_asr_unmatched$location))

# 手动修正未匹配的国家名称（针对灰色国家）

country_asr <- country_asr %>%

mutate(location = case_when(

# 修正用户列出的灰色国家名称

location == "Australia" ~ "Australia", # 确保名称一致

location == "New Zealand" ~ "New Zealand",

location == "Japan" ~ "Japan",

location == "Republic of Korea" ~ "South Korea",

location == "Democratic Socialist Republic of Sri Lanka" ~ "Sri Lanka",

location == "Republic of Yemen" ~ "Yemen",

location == "Islamic Republic of Mauritania" ~ "Mauritania",

location == "Plurinational State of Bolivia" ~ "Bolivia",

location == "Republic of Peru" ~ "Peru",

location == "Co-operative Republic of Guyana" ~ "Guyana",

location == "Republic of Guatemala" ~ "Guatemala",

location == "Portuguese Republic" ~ "Portugal",

location == "Türkiye" ~ "Türkiye", # 若地图数据使用旧名称

location == "Syrian Arab Republic" ~ "Syria",

location == "Republic of Albania" ~ "Albania",

location == "North Macedonia" ~ "North Macedonia", # 确保地图数据支持此名称

location == "Republic of Montenegro" ~ "Montenegro",

location == "Bosnia and Herzegovina" ~ "Bosnia and Herzegovina", # 确认拼写一致

location == "Hungary" ~ "Hungary",

location == "Republic of Slovenia" ~ "Slovenia",

location == "Slovak Republic" ~ "Slovakia",

location == "Czechia" ~ "Czech Republic",

location == "Kingdom of the Netherlands" ~ "Netherlands",

location == "Republic of Finland" ~ "Finland",

location == "Republic of Fiji" ~ "Fiji",

# 处理其他未匹配的 NA 和特殊地区

is.na(location) ~ NA_character_, # 保留 NA 但后续过滤

location == "Tokelau" ~ "Tokelau", # 若地图数据不包含，需过滤

location == "Tuvalu" ~ "Tuvalu", # 同上

TRUE ~ location

)) %>%

filter(!is.na(location)) # 移除 NA 值

# 特殊处理：若地图数据使用 "Swaziland" 而非 "Eswatini"

country_asr$location[country_asr$location == "Eswatini"] <- "Swaziland"

# 处理地图数据中不包含的小区域（如 Tokelau）

country_asr <- country_asr %>%

filter(!location %in% c("Tokelau", "Tuvalu"))

# 再次检查未匹配国家

world_regions <- unique(worldData$region)

country_asr_unmatched <- country_asr[!country_asr$location %in% world_regions, ]

print(unique(country_asr_unmatched$location)) # 应返回空或仅无关区域

anti_join(worldData, country_asr, by = c("region" = "location")) %>% distinct(region)

#2021年的全年龄段粗死亡率 infections1---------------------------

CDR_2021 <- read.csv("204countryDeaths.csv",header = T,check.names = F)

unique(CDR_2021$cause_name)

CDR_2021.1 <- subset(

CDR_2021, # 数据框

cause_name == 'HIV/AIDS and sexually transmitted infections' # 筛选条件

)

CDR_2021.1 <- subset(CDR_2021.1,CDR_2021.1$year==2021 &

CDR_2021.1$age_name=='All ages' &

CDR_2021.1$metric_name== 'Rate' &

CDR_2021.1$measure_name=='Deaths'&

CDR_2021.1$sex_name=='Both')

CDR_2021.1 <- CDR_2021.1[,c(4,16,17,18)] ###选择位置与数据+95%UI

CDR_2021.1$val <- round(CDR_2021.1$val,2) ###率保留3位小数点

CDR_2021.1$lower <- round(CDR_2021.1$lower,2)

CDR_2021.1$upper <- round(CDR_2021.1$upper,2)

#### map for ASMR

worldData <- map_data('world')

country_asr <- CDR_2021.1

country_asr$location <- as.character(country_asr$location_name)

###以下代码的目的是让country_asr$location的国家名称与worldData的国家名称一致

### 这样才能让数据映射到地图上

country_asr$location[country_asr$location == 'United States of America'] = 'USA'

country_asr$location[country_asr$location == 'Russian Federation'] = 'Russia'

country_asr$location[country_asr$location == 'United Kingdom'] = 'UK'

country_asr$location[country_asr$location == 'Congo'] = 'Republic of Congo'

country_asr$location[country_asr$location == "Iran (Islamic Republic of)"] = 'Iran'

country_asr$location[country_asr$location == "Democratic People's Republic of Korea"] = 'North Korea'

country_asr$location[country_asr$location == "Taiwan (Province of China)"] = 'Taiwan'

country_asr$location[country_asr$location == "Republic of Korea"] = 'South Korea'

country_asr$location[country_asr$location == "United Republic of Tanzania"] = 'Tanzania'

country_asr$location[country_asr$location == "C?te d'Ivoire"] = 'Saint Helena'

country_asr$location[country_asr$location == "Bolivia (Plurinational State of)"] = 'Bolivia'

country_asr$location[country_asr$location == "Venezuela (Bolivarian Republic of)"] = 'Venezuela'

country_asr$location[country_asr$location == "Czechia"] = 'Czech Republic'

country_asr$location[country_asr$location == "Republic of Moldova"] = 'Moldova'

country_asr$location[country_asr$location == "Viet Nam"] = 'Vietnam'

country_asr$location[country_asr$location == "Lao People's Democratic Republic"] = 'Laos'

country_asr$location[country_asr$location == "Syrian Arab Republic"] = 'Syria'

country_asr$location[country_asr$location == "North Macedonia"] = 'Macedonia'

country_asr$location[country_asr$location == "Micronesia (Federated States of)"] = 'Micronesia'

country_asr$location[country_asr$location == "Macedonia"] = 'North Macedonia'

country_asr$location[country_asr$location == "Trinidad and Tobago"] = 'Trinidad'

country_asr <- rbind(country_asr,country_asr[country_asr$location == "Trinidad",])

country_asr$location[country_asr$location == "Trinidad"] = 'Tobago'

country_asr$location[country_asr$location == "Cabo Verde"] = 'Cape Verde'

country_asr$location[country_asr$location == "United States Virgin Islands"] = 'Virgin Islands'

country_asr$location[country_asr$location == "Antigua and Barbuda"] = 'Antigu'

country_asr <- rbind(country_asr,country_asr[country_asr$location == "Antigu",])

country_asr$location[country_asr$location == "Antigu"] = 'Barbuda'

country_asr$location[country_asr$location == "Saint Kitts and Nevis"] = 'Saint Kitts'

country_asr <- rbind(country_asr,country_asr[country_asr$location == "Saint Kitts",])

country_asr$location[country_asr$location == "Saint Kitts"] = 'Nevis'

country_asr$location[country_asr$location == "Côte d'Ivoire"] = 'Ivory Coast'

country_asr$location[country_asr$location == "Saint Vincent and the Grenadines"] = 'Saint Vincent'

country_asr <- rbind(country_asr,country_asr[country_asr$location == "Saint Vincent",])

country_asr$location[country_asr$location == "Saint Vincent"] = 'Grenadines'

country_asr$location[country_asr$location == "Eswatini"] = 'Swaziland'

country_asr$location[country_asr$location == "Brunei Darussalam"] = 'Brunei'

country_asr$location[country_asr$location == "Türkiye"] = 'Turkey'

#write.csv(country_asr,"ASMR_204.csv")

total <- full_join(worldData,country_asr,by = c('region'='location'))##把两个数据根据地点合并起来

mycolor2<-rev(brewer.pal(6, "Spectral") ) ##选择我们觉得好看的颜色，这里使用了brewer.pal

#pdf("2019_ASR_Incidence_map.pdf")

summary(total$val)

quantile(country_asr$val,seq(0.1,1,0.1))

total <- total %>% mutate(val2 = cut(val, breaks = c(0.00,0.07,0.22,0.68,1.15,1.81,7.79),

labels = c("<0.07","0.07~0.22","0.22~0.68","0.68~1.15","1.15~1.81",

"1.81~7.79"), ## breaks需要根据自己的实际结果来调整

include.lowest = T,right = T))

p <- ggplot()

p2 <- p + geom_polygon(data=total,

aes(x=long, y=lat, group = group,fill=val2),

colour="gray",size = .01) +

scale_fill_manual(values = mycolor2) +

theme_void()+labs(x="", y="")+

guides(fill = guide_legend(title='CMR(/10^5)'))+

theme(legend.position = 'right')

p2

###修改后

library(countrycode)

# 将 country_asr 的 location 转换为 ISO3C 代码（标准化中间步骤）

country_asr$iso3c <- countrycode(

sourcevar = country_asr$location_name,

origin = "country.name",

destination = "iso3c"

)

# 从 ISO3C 代码转回地图数据中的通用名称

country_asr$location <- countrycode(

sourcevar = country_asr$iso3c,

origin = "iso3c",

destination = "country.name"

)

# 特殊处理个别名称（如 USA）

country_asr$location[country_asr$location_name == "United States of America"] <- "USA"

#查找未匹配国家

world_regions <- unique(worldData$region)

country_asr_unmatched <- country_asr[!country_asr$location %in% world_regions, ]

print(country_asr_unmatched$location)

# 手动修正特殊名称

country_asr$location <- case_when(

country_asr$location_name == "People's Republic of China" ~ "China",

country_asr$location_name == "Democratic Republic of the Congo" ~ "DR Congo",

country_asr$location_name == "Russian Federation" ~ "Russia",

TRUE ~ country_asr$location

)

# 手动修正未匹配的国家名称

country_asr <- country_asr %>%

mutate(location = case_when(

# 逐个修正

location == "Myanmar (Burma)" ~ "Myanmar",

location == "Micronesia (Federated States of)" ~ "Micronesia",

location == "Bosnia & Herzegovina" ~ "Bosnia and Herzegovina",

location == "Czechia" ~ "Czech Republic",

location == "United Kingdom" ~ "UK",

location == "Antigua & Barbuda" ~ "Antigua",

location == "St. Vincent & Grenadines" ~ "Saint Vincent and the Grenadines",

location == "St. Lucia" ~ "Saint Lucia",

location == "Trinidad & Tobago" ~ "Trinidad and Tobago",

location == "Palestinian Territories" ~ "Palestine",

location == "DR Congo" ~ "Democratic Republic of the Congo",

location == "Congo - Brazzaville" ~ "Republic of Congo",

location == "Eswatini" ~ "Swaziland", # 地图数据中仍可能用旧名

location == "Côte d’Ivoire" ~ "Ivory Coast",

location == "São Tomé & Príncipe" ~ "Sao Tome and Principe",

location == "St. Kitts & Nevis" ~ "Saint Kitts",

location == "U.S. Virgin Islands" ~ "Virgin Islands",

location == "Tokelau" ~ "Tokelau", # 地图数据可能不包含该区域

location == "Tuvalu" ~ "Tuvalu", # 同上

location == "Antigua & Barbuda" ~ "Antigua", # 重复项需统一

TRUE ~ location # 其他名称保持不变

))

# 再次检查未匹配国家（应减少至接近零）

world_regions <- unique(worldData$region)

country_asr_unmatched <- country_asr[!country_asr$location %in% world_regions, ]

print(unique(country_asr_unmatched$location))

# 手动修正未匹配的国家名称（针对灰色国家）

country_asr <- country_asr %>%

mutate(location = case_when(

# 修正用户列出的灰色国家名称

location == "Australia" ~ "Australia", # 确保名称一致

location == "New Zealand" ~ "New Zealand",

location == "Japan" ~ "Japan",

location == "Republic of Korea" ~ "South Korea",

location == "Democratic Socialist Republic of Sri Lanka" ~ "Sri Lanka",

location == "Republic of Yemen" ~ "Yemen",

location == "Islamic Republic of Mauritania" ~ "Mauritania",

location == "Plurinational State of Bolivia" ~ "Bolivia",

location == "Republic of Peru" ~ "Peru",

location == "Co-operative Republic of Guyana" ~ "Guyana",

location == "Republic of Guatemala" ~ "Guatemala",

location == "Portuguese Republic" ~ "Portugal",

location == "Türkiye" ~ "Türkiye", # 若地图数据使用旧名称

location == "Syrian Arab Republic" ~ "Syria",

location == "Republic of Albania" ~ "Albania",

location == "North Macedonia" ~ "North Macedonia", # 确保地图数据支持此名称

location == "Republic of Montenegro" ~ "Montenegro",

location == "Bosnia and Herzegovina" ~ "Bosnia and Herzegovina", # 确认拼写一致

location == "Hungary" ~ "Hungary",

location == "Republic of Slovenia" ~ "Slovenia",

location == "Slovak Republic" ~ "Slovakia",

location == "Czechia" ~ "Czech Republic",

location == "Kingdom of the Netherlands" ~ "Netherlands",

location == "Republic of Finland" ~ "Finland",

location == "Republic of Fiji" ~ "Fiji",

# 处理其他未匹配的 NA 和特殊地区

is.na(location) ~ NA_character_, # 保留 NA 但后续过滤

location == "Tokelau" ~ "Tokelau", # 若地图数据不包含，需过滤

location == "Tuvalu" ~ "Tuvalu", # 同上

TRUE ~ location

)) %>%

filter(!is.na(location)) # 移除 NA 值

# 特殊处理：若地图数据使用 "Swaziland" 而非 "Eswatini"

country_asr$location[country_asr$location == "Eswatini"] <- "Swaziland"

# 处理地图数据中不包含的小区域（如 Tokelau）

country_asr <- country_asr %>%

filter(!location %in% c("Tokelau", "Tuvalu"))

# 再次检查未匹配国家

world_regions <- unique(worldData$region)

country_asr_unmatched <- country_asr[!country_asr$location %in% world_regions, ]

print(unique(country_asr_unmatched$location)) # 应返回空或仅无关区域

anti_join(worldData, country_asr, by = c("region" = "location")) %>% distinct(region)

#2021年的全年龄段粗死亡率 infections2---------------------------------------

CDR_2021 <- read.csv("204countryDeaths.csv",header = T,check.names = F)

unique(CIR_2021$cause_name)

CDR_2021.2 <- subset(

CDR_2021, # 数据框

cause_name == 'Other infectious diseases' # 筛选条件

)

CDR_2021.2 <- subset(CDR_2021.2,CDR_2021.2$year==2021 &

CDR_2021.2$age_name=='All ages' &

CDR_2021.2$metric_name== 'Rate' &

CDR_2021.2$measure_name=='Deaths'&

CDR_2021.2$sex_name=='Both')

CDR_2021.2 <- CDR_2021.2[,c(4,16,17,18)] ###选择位置与数据+95%UI

CDR_2021.2$val <- round(CDR_2021.2$val,2) ###率保留3位小数点

CDR_2021.2$lower <- round(CDR_2021.2$lower,2)

CDR_2021.2$upper <- round(CDR_2021.2$upper,2)

#### map for ASMR

worldData <- map_data('world')

country_asr <- CDR_2021.2

country_asr$location <- as.character(country_asr$location_name)

###以下代码的目的是让country_asr$location的国家名称与worldData的国家名称一致

### 这样才能让数据映射到地图上

country_asr$location[country_asr$location == 'United States of America'] = 'USA'

country_asr$location[country_asr$location == 'Russian Federation'] = 'Russia'

country_asr$location[country_asr$location == 'United Kingdom'] = 'UK'

country_asr$location[country_asr$location == 'Congo'] = 'Republic of Congo'

country_asr$location[country_asr$location == "Iran (Islamic Republic of)"] = 'Iran'

country_asr$location[country_asr$location == "Democratic People's Republic of Korea"] = 'North Korea'

country_asr$location[country_asr$location == "Taiwan (Province of China)"] = 'Taiwan'

country_asr$location[country_asr$location == "Republic of Korea"] = 'South Korea'

country_asr$location[country_asr$location == "United Republic of Tanzania"] = 'Tanzania'

country_asr$location[country_asr$location == "C?te d'Ivoire"] = 'Saint Helena'

country_asr$location[country_asr$location == "Bolivia (Plurinational State of)"] = 'Bolivia'

country_asr$location[country_asr$location == "Venezuela (Bolivarian Republic of)"] = 'Venezuela'

country_asr$location[country_asr$location == "Czechia"] = 'Czech Republic'

country_asr$location[country_asr$location == "Republic of Moldova"] = 'Moldova'

country_asr$location[country_asr$location == "Viet Nam"] = 'Vietnam'

country_asr$location[country_asr$location == "Lao People's Democratic Republic"] = 'Laos'

country_asr$location[country_asr$location == "Syrian Arab Republic"] = 'Syria'

country_asr$location[country_asr$location == "North Macedonia"] = 'Macedonia'

country_asr$location[country_asr$location == "Micronesia (Federated States of)"] = 'Micronesia'

country_asr$location[country_asr$location == "Macedonia"] = 'North Macedonia'

country_asr$location[country_asr$location == "Trinidad and Tobago"] = 'Trinidad'

country_asr <- rbind(country_asr,country_asr[country_asr$location == "Trinidad",])

country_asr$location[country_asr$location == "Trinidad"] = 'Tobago'

country_asr$location[country_asr$location == "Cabo Verde"] = 'Cape Verde'

country_asr$location[country_asr$location == "United States Virgin Islands"] = 'Virgin Islands'

country_asr$location[country_asr$location == "Antigua and Barbuda"] = 'Antigu'

country_asr <- rbind(country_asr,country_asr[country_asr$location == "Antigu",])

country_asr$location[country_asr$location == "Antigu"] = 'Barbuda'

country_asr$location[country_asr$location == "Saint Kitts and Nevis"] = 'Saint Kitts'

country_asr <- rbind(country_asr,country_asr[country_asr$location == "Saint Kitts",])

country_asr$location[country_asr$location == "Saint Kitts"] = 'Nevis'

country_asr$location[country_asr$location == "Côte d'Ivoire"] = 'Ivory Coast'

country_asr$location[country_asr$location == "Saint Vincent and the Grenadines"] = 'Saint Vincent'

country_asr <- rbind(country_asr,country_asr[country_asr$location == "Saint Vincent",])

country_asr$location[country_asr$location == "Saint Vincent"] = 'Grenadines'

country_asr$location[country_asr$location == "Eswatini"] = 'Swaziland'

country_asr$location[country_asr$location == "Brunei Darussalam"] = 'Brunei'

country_asr$location[country_asr$location == "Türkiye"] = 'Turkey'

#write.csv(country_asr,"ASMR_204.csv")

total <- full_join(worldData,country_asr,by = c('region'='location'))##把两个数据根据地点合并起来

mycolor2<-rev(brewer.pal(4, "Spectral") ) ##选择我们觉得好看的颜色，这里使用了brewer.pal

#pdf("2019_ASR_Incidence_map.pdf")

summary(total$val)

quantile(country_asr$val,seq(0.1,1,0.1))

total <- total %>% mutate(val2 = cut(val, breaks = c(0.000,0.001,0.010,0.030,0.240),

labels = c("<0.001","0.001~0.010","0.010~0.030","0.030~0.240"), ## breaks需要根据自己的实际结果来调整

include.lowest = T,right = T))

p <- ggplot()

p2.2 <- p + geom_polygon(data=total,

aes(x=long, y=lat, group = group,fill=val2),

colour="gray",size = .01) +

scale_fill_manual(values = mycolor2) +

theme_void()+labs(x="", y="")+

guides(fill = guide_legend(title='CMR(/10^5)'))+

theme(legend.position = 'right')

p2.2

###修改后

library(countrycode)

# 将 country_asr 的 location 转换为 ISO3C 代码（标准化中间步骤）

country_asr$iso3c <- countrycode(

sourcevar = country_asr$location_name,

origin = "country.name",

destination = "iso3c"

)

# 从 ISO3C 代码转回地图数据中的通用名称

country_asr$location <- countrycode(

sourcevar = country_asr$iso3c,

origin = "iso3c",

destination = "country.name"

)

# 特殊处理个别名称（如 USA）

country_asr$location[country_asr$location_name == "United States of America"] <- "USA"

#查找未匹配国家

world_regions <- unique(worldData$region)

country_asr_unmatched <- country_asr[!country_asr$location %in% world_regions, ]

print(country_asr_unmatched$location)

# 手动修正特殊名称

country_asr$location <- case_when(

country_asr$location_name == "People's Republic of China" ~ "China",

country_asr$location_name == "Democratic Republic of the Congo" ~ "DR Congo",

country_asr$location_name == "Russian Federation" ~ "Russia",

TRUE ~ country_asr$location

)

# 手动修正未匹配的国家名称

country_asr <- country_asr %>%

mutate(location = case_when(

# 逐个修正

location == "Myanmar (Burma)" ~ "Myanmar",

location == "Micronesia (Federated States of)" ~ "Micronesia",

location == "Bosnia & Herzegovina" ~ "Bosnia and Herzegovina",

location == "Czechia" ~ "Czech Republic",

location == "United Kingdom" ~ "UK",

location == "Antigua & Barbuda" ~ "Antigua",

location == "St. Vincent & Grenadines" ~ "Saint Vincent and the Grenadines",

location == "St. Lucia" ~ "Saint Lucia",

location == "Trinidad & Tobago" ~ "Trinidad and Tobago",

location == "Palestinian Territories" ~ "Palestine",

location == "DR Congo" ~ "Democratic Republic of the Congo",

location == "Congo - Brazzaville" ~ "Republic of Congo",

location == "Eswatini" ~ "Swaziland", # 地图数据中仍可能用旧名

location == "Côte d’Ivoire" ~ "Ivory Coast",

location == "São Tomé & Príncipe" ~ "Sao Tome and Principe",

location == "St. Kitts & Nevis" ~ "Saint Kitts",

location == "U.S. Virgin Islands" ~ "Virgin Islands",

location == "Tokelau" ~ "Tokelau", # 地图数据可能不包含该区域

location == "Tuvalu" ~ "Tuvalu", # 同上

location == "Antigua & Barbuda" ~ "Antigua", # 重复项需统一

TRUE ~ location # 其他名称保持不变

))

# 再次检查未匹配国家（应减少至接近零）

world_regions <- unique(worldData$region)

country_asr_unmatched <- country_asr[!country_asr$location %in% world_regions, ]

print(unique(country_asr_unmatched$location))

# 手动修正未匹配的国家名称（针对灰色国家）

country_asr <- country_asr %>%

mutate(location = case_when(

# 修正用户列出的灰色国家名称

location == "Australia" ~ "Australia", # 确保名称一致

location == "New Zealand" ~ "New Zealand",

location == "Japan" ~ "Japan",

location == "Republic of Korea" ~ "South Korea",

location == "Democratic Socialist Republic of Sri Lanka" ~ "Sri Lanka",

location == "Republic of Yemen" ~ "Yemen",

location == "Islamic Republic of Mauritania" ~ "Mauritania",

location == "Plurinational State of Bolivia" ~ "Bolivia",

location == "Republic of Peru" ~ "Peru",

location == "Co-operative Republic of Guyana" ~ "Guyana",

location == "Republic of Guatemala" ~ "Guatemala",

location == "Portuguese Republic" ~ "Portugal",

location == "Türkiye" ~ "Türkiye", # 若地图数据使用旧名称

location == "Syrian Arab Republic" ~ "Syria",

location == "Republic of Albania" ~ "Albania",

location == "North Macedonia" ~ "North Macedonia", # 确保地图数据支持此名称

location == "Republic of Montenegro" ~ "Montenegro",

location == "Bosnia and Herzegovina" ~ "Bosnia and Herzegovina", # 确认拼写一致

location == "Hungary" ~ "Hungary",

location == "Republic of Slovenia" ~ "Slovenia",

location == "Slovak Republic" ~ "Slovakia",

location == "Czechia" ~ "Czech Republic",

location == "Kingdom of the Netherlands" ~ "Netherlands",

location == "Republic of Finland" ~ "Finland",

location == "Republic of Fiji" ~ "Fiji",

# 处理其他未匹配的 NA 和特殊地区

is.na(location) ~ NA_character_, # 保留 NA 但后续过滤

location == "Tokelau" ~ "Tokelau", # 若地图数据不包含，需过滤

location == "Tuvalu" ~ "Tuvalu", # 同上

TRUE ~ location

)) %>%

filter(!is.na(location)) # 移除 NA 值

# 特殊处理：若地图数据使用 "Swaziland" 而非 "Eswatini"

country_asr$location[country_asr$location == "Eswatini"] <- "Swaziland"

# 处理地图数据中不包含的小区域（如 Tokelau）

country_asr <- country_asr %>%

filter(!location %in% c("Tokelau", "Tuvalu"))

# 再次检查未匹配国家

world_regions <- unique(worldData$region)

country_asr_unmatched <- country_asr[!country_asr$location %in% world_regions, ]

print(unique(country_asr_unmatched$location)) # 应返回空或仅无关区域

anti_join(worldData, country_asr, by = c("region" = "location")) %>% distinct(region)

**PART 7 function_year5**

function_year5 <- function(table_name, start_year, end_year, current_year){

remain <- current_year - floor((current_year - start_year)/5) * 5

year_names <- NULL

for (i in start_year:end_year) {

if((i - current_year)/5 - floor((i - current_year)/5) == 0){

if(i == remain){

temp <- paste(start_year, i, sep = '-')

year_names <- append(year_names, temp)

}

else{

temp <- paste(i-4, i, sep = '-')

year_names <- append(year_names, temp)

}

}

}

table_name <- as.data.frame(table_name)

new_years <- seq(start_year,end_year,1)

new_table <- as.data.frame(matrix(data = rep(0, length(year_names)*nrow(table_name)), ncol = length(year_names), nrow = nrow(table_name))) %>% as.data.frame()

colnames(new_table) <- year_names

j = 1

for (i in 1:(end_year - start_year + 1)){

if((new_years[i] - current_year)/5 - floor((new_years[i] - current_year)/5) != 0){

new_table[, year_names[j]] <- new_table[,year_names[j]] + table_name[,as.character(new_years[i])]

}

else{

if(j == 1){

new_table[,year_names[j]] <- (new_table[,year_names[j]] + table_name[,as.character(new_years[i])]) / (remain - start_year + 1)

}

else{

new_table[,year_names[j]] <- (new_table[,year_names[j]] + table_name[,as.character(new_years[i])]) / 5

}

j = j + 1

}

}

return(new_table)

}

**PART 8 frontier analysis**

setwd("D:/frontier analysis")

## frontier analysis################

library(dplyr)

library(data.table)

library(purrr)

library(tidyr)

library(ggplot2)

library(ggrepel)

#####导入数据####

case<- vroom::vroom("D:\\课程材料\\代码及实操\\实操\\IBD_country\\IBD_country.csv")

unique(case$age_name)

case<- subset(case,case$age_name=="All ages"|case$age_name=="Age-standardized" )

SDI <- read.csv('SDI_2021.csv',header = T)

## 合并SDI值

frontier_data <- subset(case,case$age_name=='Age-standardized' &

case$metric_name== 'Rate' &

case$sex_name =='Both' &

case$measure_name=='DALYs (Disability-Adjusted Life Years)')

frontier_data<-frontier_data[,c(5,14,15)]#####选出location year val

frontier_SDI <- left_join(frontier_data,SDI,by=c('location_name','year')) #####加上SDI数据

boostrap_DEA <- as.data.frame(matrix(nrow=0,ncol=6))

names(boostrap_DEA) <- c(names(frontier_SDI),'frontier','super')

boostrap_num <- 100 ### boostrap次数

for(interation_number in 1:boostrap_num){

boot_sample <- frontier_SDI[sample(1:nrow(frontier_SDI),nrow(frontier_SDI),replace = TRUE),] ## 有放回抽样，抽样nrow(frontier_SDI)次

boot_sample <- boot_sample %>% arrange(SDI,desc(val)) ## 我们对SDI从小到大排列、ASR从大到小排列进行排列

boot_sample$super <- NA

boot_sample$super[1] <- 0 ## super变量是用来判断是否是super-efficient

for (i in 2:nrow(frontier_SDI)) {

data <- boot_sample[-i,] ## 对每个点进行排除，判断是否是super-efficient

data$frontier <- NA ## 产生frontier变量

min <- data$val[1] ## ASR已从大到小排列，因此第一点是默认的frontier点

for (j in 1:(i-1)) { ## 为了节约计算空间，我们只需要对剔除点以前的数据生成frontier值来判断这个点是否是super-efficient

min <- ifelse(data$val[j]<min,data$val[j],min)

data$frontier[j] <- min} ## 判断该点是否比frontier值小，如果是，成为新的frontier

boot_sample$super[i] <- ifelse(boot_sample[i,1]==boot_sample[i+1,1] & boot_sample[i,2]==boot_sample[i+1,2],0,

ifelse(boot_sample$val[i]<data$frontier[i-1],0,1)) ## 判断排除的点是否是super-efficient,两种情况

###，一种是排除的点和后面的点是同一个值，那就默认不是super-efficient；如果不和后一个点一样，就判断ASR和前一个点的frontier的大小，

###如果小于frontier,就认为是super-efficient

}

## 排除super-efficient后计算每个点的frontier值

boot_sample_exclude <- boot_sample[boot_sample$super==0,]

min <- boot_sample_exclude$val[1]

for (z in 1:nrow(boot_sample_exclude)){

min <- ifelse(boot_sample_exclude$val[z]<min,boot_sample_exclude$val[z],min)

boot_sample_exclude$frontier[z] <- min

}

boostrap_DEA <- rbind(boostrap_DEA,boot_sample_exclude)

boostrap_DEA <-boostrap_DEA %>%

group_by(location_name,year,val,SDI) %>%

summarize(frontier=mean(frontier))

print(interation_number)

}

load(file='boostrap.Rdata')

boostrap_DEA <- boostrap_DEA %>%

mutate(eff_diff = val - frontier)

boostrap_DEA_1990 <- boostrap_DEA %>%

filter(year == 1990)

boostrap_DEA_2021 <- boostrap_DEA %>%

filter(year == 2021)

boostrap_DEA_2021$trend <- ifelse(boostrap_DEA_2021$val > boostrap_DEA_1990$val, "Increase",

"Decrease")

plotA <- ggplot(boostrap_DEA, aes(SDI,val)) + geom_point(aes(color = year),size=1.8)+

scale_x_continuous(breaks = c(0,0.2,0.4,0.6,0.8,1.0),limits = c(0,1)) +

scale_y_reverse() + ## y轴旋转

scale_color_gradient(low='#62b1d0',high='#1047a9') + ## 连续性变量配色

stat_smooth(data=boostrap_DEA, aes(SDI,frontier),colour='black',formula=y ~ poly(x, 1),

stat = "smooth",method='loess',se=F,span=0.2,fullrange=T) + ## 绘制frontier线

theme_bw()

plotA

black <- boostrap_DEA_2021[order(boostrap_DEA_2021$eff_diff,decreasing = T),][1:15,] ## 获取距离差最大的15个点

blue <- subset(boostrap_DEA_2021,SDI<0.5)[order(subset(boostrap_DEA_2021,SDI<0.5)$eff_diff),][1:5,] ## 低SDI国家中距离差最小的5个国家

red <- subset(boostrap_DEA_2021,SDI>0.85)[order(subset(boostrap_DEA_2021,SDI>0.85)$eff_diff,decreasing = T),][1:5,] ## 高SDI国家中距离差最大的5个国家

plotB <- ggplot(boostrap_DEA_2021, aes(SDI,val)) + geom_point(aes(color = trend),size=2.5)+

scale_x_continuous(breaks = c(0,0.2,0.4,0.6,0.8,1.0),limits = c(0,1)) + ## x轴标度设定

scale_y_reverse() + ## y轴调转

stat_smooth(data=boostrap_DEA, aes(SDI,frontier),colour='black',formula=y ~ poly(x, 1),

stat = "smooth",method='loess',se=F,span=0.2,fullrange=T) + ## 画frontier拟合线

geom_text_repel(data=black,colour='black',aes(SDI,val, label = location_name),size=2.5,fontface= 'bold',max.overlaps = 160) + ##添加距离差最大的15个地区名称，并标记在对应的点上

geom_text_repel(data=red,colour='darkred',aes(SDI,val, label = location_name),size=2.5,fontface= 'bold',max.overlaps = 160) + ##添加高SDI国家中距离差最大的5个国家，并标记在对应的点上，用红色字体现实

geom_text_repel(data=blue,colour='darkblue',aes(SDI,val, label = location_name),size=2.5,fontface= 'bold',max.overlaps = 160) + ##添加低SDI国家中距离差最小的5个国家，并标记在对应的点上，用蓝色字体现实

theme_bw()

plotB

**PART 9 The EAPC in ASDR in 204 countries and territories from 1990 to 2021**

##The EAPC in ASDR in 204 countries and territories from 1990 to 2021.

CDR <- read.csv("204countryDeaths1990.2020.csv",header = T,check.names = F)

unique(CDR$cause_name)

CDR_1 <- subset(

CDR, # 数据框

cause_name == 'HIV/AIDS and sexually transmitted infections' # 筛选条件

)

CDR_1 <- subset(CDR_1,CDR_1$year==2021|1990 &

CDR_1$age_name=='All ages' &

CDR_1$metric_name== 'Rate' &

CDR_1$measure_name=='Deaths'&

CDR_1$sex_name=='Both')

CDR_1 <- CDR_1[,c(4,15,16,17,18)] ###选择位置与数据+95%UI

write.csv(CDR_1,"CDR_1.csv")

library(tidyverse)

# 读取数据

# 加载必要的包

library(dplyr)

# 读取数据

data <- read.csv("CDR_1.csv", stringsAsFactors = FALSE)

# 按国家和年份排序

data_sorted <- data %>%

arrange(location_name, year)

# 计算EAPC函数

calculate_eapc <- function(group) {

# 检查是否有1990和2021年的数据

if (nrow(group) != 2 || !all(c(1990, 2021) %in% group$year)) {

message(sprintf("跳过 %s，数据点不足或年份不完整", group$location_name[1]))

return(NULL)

}

# 提取年份和值

y0 <- group$val[group$year == 1990]

y1 <- group$val[group$year == 2021]

delta_t <- 2021 - 1990 # 时间跨度固定为31年

# 计算对数增长率

beta <- (log(y1) - log(y0)) / delta_t

eapc <- (exp(beta) - 1) * 100 # 转换为百分比

# 返回结果

data.frame(

location = group$location_name[1],

EAPC = eapc,

stringsAsFactors = FALSE

)

}

# 分组计算EAPC

eapc_results <- data_sorted %>%

group_by(location_name) %>%

group_map(~ calculate_eapc(.x), .keep = TRUE) %>%

bind_rows()

# 输出结果

print(eapc_results)

write.csv(eapc_results,"eapc_results1")

##地图绘制---------------

eapc_results$EAPC <- round(eapc_results$EAPC,2) ###率保留2位小数点

#### map for ASMR

worldData <- map_data('world') #####使用map包提取世界·范围内的地图

country_asr <- eapc_results ####

###以下代码的目的是让country_asr$location的国家名称与worldData的国家名称一致

### 这样才能让数据映射到地图上

country_asr$location[country_asr$location == 'United States of America'] = 'USA'

country_asr$location[country_asr$location == 'Russian Federation'] = 'Russia'

country_asr$location[country_asr$location == 'United Kingdom'] = 'UK'

country_asr$location[country_asr$location == 'Congo'] = 'Republic of Congo'

country_asr$location[country_asr$location == "Iran (Islamic Republic of)"] = 'Iran'

country_asr$location[country_asr$location == "Democratic People's Republic of Korea"] = 'North Korea'

country_asr$location[country_asr$location == "Taiwan (Province of China)"] = 'Taiwan'

country_asr$location[country_asr$location == "Republic of Korea"] = 'South Korea'

country_asr$location[country_asr$location == "United Republic of Tanzania"] = 'Tanzania'

country_asr$location[country_asr$location == "C?te d'Ivoire"] = 'Saint Helena'

country_asr$location[country_asr$location == "Bolivia (Plurinational State of)"] = 'Bolivia'

country_asr$location[country_asr$location == "Venezuela (Bolivarian Republic of)"] = 'Venezuela'

country_asr$location[country_asr$location == "Czechia"] = 'Czech Republic'

country_asr$location[country_asr$location == "Republic of Moldova"] = 'Moldova'

country_asr$location[country_asr$location == "Viet Nam"] = 'Vietnam'

country_asr$location[country_asr$location == "Lao People's Democratic Republic"] = 'Laos'

country_asr$location[country_asr$location == "Syrian Arab Republic"] = 'Syria'

country_asr$location[country_asr$location == "North Macedonia"] = 'Macedonia'

country_asr$location[country_asr$location == "Micronesia (Federated States of)"] = 'Micronesia'

country_asr$location[country_asr$location == "Macedonia"] = 'North Macedonia'

country_asr$location[country_asr$location == "Trinidad and Tobago"] = 'Trinidad'

country_asr <- rbind(country_asr,country_asr[country_asr$location == "Trinidad",])

country_asr$location[country_asr$location == "Trinidad"] = 'Tobago'

country_asr$location[country_asr$location == "Cabo Verde"] = 'Cape Verde'

country_asr$location[country_asr$location == "United States Virgin Islands"] = 'Virgin Islands'

country_asr$location[country_asr$location == "Antigua and Barbuda"] = 'Antigu'

country_asr <- rbind(country_asr,country_asr[country_asr$location == "Antigu",])

country_asr$location[country_asr$location == "Antigu"] = 'Barbuda'

country_asr$location[country_asr$location == "Saint Kitts and Nevis"] = 'Saint Kitts'

country_asr <- rbind(country_asr,country_asr[country_asr$location == "Saint Kitts",])

country_asr$location[country_asr$location == "Saint Kitts"] = 'Nevis'

country_asr$location[country_asr$location == "Côte d'Ivoire"] = 'Ivory Coast'

country_asr$location[country_asr$location == "Saint Vincent and the Grenadines"] = 'Saint Vincent'

country_asr <- rbind(country_asr,country_asr[country_asr$location == "Saint Vincent",])

country_asr$location[country_asr$location == "Saint Vincent"] = 'Grenadines'

country_asr$location[country_asr$location == "Eswatini"] = 'Swaziland'

country_asr$location[country_asr$location == "Brunei Darussalam"] = 'Brunei'

country_asr$location[country_asr$location == "Türkiye"] = 'Turkey'

unique(country_asr$location)

#write.csv(country_asr,"ASDR_204.1.csv")

total <- full_join(worldData,country_asr,by = c('region'='location'))##把两个数据根据地点合并起来

#mycolor2<-rev(brewer.pal(5, "Spectral") ) ##选择我们觉得好看的颜色，这里使用了brewer.pal

summary(total$EAPC)

quantile(total$EAPC,seq(0.1,1,0.1),na.rm = T)

#######修改

# 加载必要包

library(ggplot2)

library(sf) # 现代地理空间数据处理

#install.packages('rnaturalearth')

library(rnaturalearth) # 获取世界地图数据

# 1. 准备数据（假设已有EAPC数据框名为eapc_results）

# 示例数据结构：

# eapc_results <- data.frame(

# Country = c("China", "United States", ...),

# EAPC = c(2.5, -1.3, ...)

# )

# 2. 获取世界地图数据

world <- ne_countries(scale = "medium", returnclass = "sf")

# 3. 将EAPC数据与地图数据合并（需要匹配国家名称）

# 注意：如果名称不匹配，可以用countrycode包转换ISO代码

library(countrycode)

eapc_results <- eapc_results %>%

mutate(iso_a3 = countrycode(location, "country.name", "iso3c"))

world_data <- world %>%

left_join(eapc_results, by = c("iso_a3" = "iso_a3"))

#-8.08,-6.63,-3.16,0,2.42,6.18,10.69,30

# 4. 绘制地图

ggplot(data = world_data) +

geom_sf(aes(fill = EAPC), color = "white", size = 0.2) + # 填充EAPC值

scale_fill_gradientn(

colors = c("#2c7bb6", "#5aa3c9","#89c6d8","#b3d0c2","#e8c6a1","#ec8a7a","#d94d4d", "#a50026"),

values = scales::rescale(c(-10,-5,0,5,10,15)), # 根据数据范围调整

labels = c("-10", "-5","0", "-5","10","15"),

na.value = "grey90" ) +

labs(

title = "Country Trends of EAPC (1990-2021)"

) +

theme_minimal() +

theme(

plot.title = element_text(hjust = 0.5, size = 16, face = "bold"),

legend.position = "right",

legend.key.width = unit(0.6, "cm"),

panel.grid = element_blank()

)

# 5. 保存图片（可选）

#ggsave("eapc_world_map.png", width = 12, height = 8, dpi = 300)

#infections2------------------------------------------------------------

CDR_2 <- subset(

CDR, # 数据框

cause_name == 'Other infectious diseases' # 筛选条件

)

CDR_2 <- subset(CDR_2,CDR_2$year==2021|1990 &

CDR_2$age_name=='All ages' &

CDR_2$metric_name== 'Rate' &

CDR_2$measure_name=='Deaths'&

CDR_2$sex_name=='Both')

CDR_2 <- CDR_2[,c(4,15,16,17,18)] ###选择位置与数据+95%UI

write.csv(CDR_2,"CDR_2.csv")

library(tidyverse)

# 读取数据

# 加载必要的包

library(dplyr)

# 读取数据

data <- read.csv("CDR_2.csv", stringsAsFactors = FALSE)

# 按国家和年份排序

data_sorted <- data %>%

arrange(location_name, year)

# 计算EAPC函数

calculate_eapc <- function(group) {

# 检查是否有1990和2021年的数据

if (nrow(group) != 2 || !all(c(1990, 2021) %in% group$year)) {

message(sprintf("跳过 %s，数据点不足或年份不完整", group$location_name[1]))

return(NULL)

}

# 提取年份和值

y0 <- group$val[group$year == 1990]

y1 <- group$val[group$year == 2021]

delta_t <- 2021 - 1990 # 时间跨度固定为31年

# 计算对数增长率

beta <- (log(y1) - log(y0)) / delta_t

eapc <- (exp(beta) - 1) * 100 # 转换为百分比

# 返回结果

data.frame(

location = group$location_name[1],

EAPC = eapc,

stringsAsFactors = FALSE

)

}

# 分组计算EAPC

eapc_results <- data_sorted %>%

group_by(location_name) %>%

group_map(~ calculate_eapc(.x), .keep = TRUE) %>%

bind_rows()

# 输出结果

print(eapc_results)

write.csv(eapc_results,"eapc_results2.csv")

##地图绘制---------------

eapc_results$EAPC <- round(eapc_results$EAPC,2) ###率保留2位小数点

#### map for ASMR

worldData <- map_data('world') #####使用map包提取世界·范围内的地图

country_asr <- eapc_results ####

###以下代码的目的是让country_asr$location的国家名称与worldData的国家名称一致

### 这样才能让数据映射到地图上

country_asr$location[country_asr$location == 'United States of America'] = 'USA'

country_asr$location[country_asr$location == 'Russian Federation'] = 'Russia'

country_asr$location[country_asr$location == 'United Kingdom'] = 'UK'

country_asr$location[country_asr$location == 'Congo'] = 'Republic of Congo'

country_asr$location[country_asr$location == "Iran (Islamic Republic of)"] = 'Iran'

country_asr$location[country_asr$location == "Democratic People's Republic of Korea"] = 'North Korea'

country_asr$location[country_asr$location == "Taiwan (Province of China)"] = 'Taiwan'

country_asr$location[country_asr$location == "Republic of Korea"] = 'South Korea'

country_asr$location[country_asr$location == "United Republic of Tanzania"] = 'Tanzania'

country_asr$location[country_asr$location == "C?te d'Ivoire"] = 'Saint Helena'

country_asr$location[country_asr$location == "Bolivia (Plurinational State of)"] = 'Bolivia'

country_asr$location[country_asr$location == "Venezuela (Bolivarian Republic of)"] = 'Venezuela'

country_asr$location[country_asr$location == "Czechia"] = 'Czech Republic'

country_asr$location[country_asr$location == "Republic of Moldova"] = 'Moldova'

country_asr$location[country_asr$location == "Viet Nam"] = 'Vietnam'

country_asr$location[country_asr$location == "Lao People's Democratic Republic"] = 'Laos'

country_asr$location[country_asr$location == "Syrian Arab Republic"] = 'Syria'

country_asr$location[country_asr$location == "North Macedonia"] = 'Macedonia'

country_asr$location[country_asr$location == "Micronesia (Federated States of)"] = 'Micronesia'

country_asr$location[country_asr$location == "Macedonia"] = 'North Macedonia'

country_asr$location[country_asr$location == "Trinidad and Tobago"] = 'Trinidad'

country_asr <- rbind(country_asr,country_asr[country_asr$location == "Trinidad",])

country_asr$location[country_asr$location == "Trinidad"] = 'Tobago'

country_asr$location[country_asr$location == "Cabo Verde"] = 'Cape Verde'

country_asr$location[country_asr$location == "United States Virgin Islands"] = 'Virgin Islands'

country_asr$location[country_asr$location == "Antigua and Barbuda"] = 'Antigu'

country_asr <- rbind(country_asr,country_asr[country_asr$location == "Antigu",])

country_asr$location[country_asr$location == "Antigu"] = 'Barbuda'

country_asr$location[country_asr$location == "Saint Kitts and Nevis"] = 'Saint Kitts'

country_asr <- rbind(country_asr,country_asr[country_asr$location == "Saint Kitts",])

country_asr$location[country_asr$location == "Saint Kitts"] = 'Nevis'

country_asr$location[country_asr$location == "Côte d'Ivoire"] = 'Ivory Coast'

country_asr$location[country_asr$location == "Saint Vincent and the Grenadines"] = 'Saint Vincent'

country_asr <- rbind(country_asr,country_asr[country_asr$location == "Saint Vincent",])

country_asr$location[country_asr$location == "Saint Vincent"] = 'Grenadines'

country_asr$location[country_asr$location == "Eswatini"] = 'Swaziland'

country_asr$location[country_asr$location == "Brunei Darussalam"] = 'Brunei'

country_asr$location[country_asr$location == "Türkiye"] = 'Turkey'

unique(country_asr$location)

#write.csv(country_asr,"ASDR_204.1.csv")

total <- full_join(worldData,country_asr,by = c('region'='location'))##把两个数据根据地点合并起来

mycolor2<-rev(brewer.pal(5, "Spectral") ) ##选择我们觉得好看的颜色，这里使用了brewer.pal

summary(total$EAPC)

quantile(total$EAPC,seq(0.1,1,0.1),na.rm = T)

#######修改

# 加载必要包

library(ggplot2)

library(sf) # 现代地理空间数据处理

#install.packages('rnaturalearth')

library(rnaturalearth) # 获取世界地图数据

# 1. 准备数据（假设已有EAPC数据框名为eapc_results）

# 示例数据结构：

# eapc_results <- data.frame(

# Country = c("China", "United States", ...),

# EAPC = c(2.5, -1.3, ...)

# )

# 2. 获取世界地图数据

world <- ne_countries(scale = "medium", returnclass = "sf")

# 3. 将EAPC数据与地图数据合并（需要匹配国家名称）

# 注意：如果名称不匹配，可以用countrycode包转换ISO代码

library(countrycode)

eapc_results <- eapc_results %>%

mutate(iso_a3 = countrycode(location, "country.name", "iso3c"))

world_data <- world %>%

left_join(eapc_results, by = c("iso_a3" = "iso_a3"))

# 10% 20% 30% 40% 50% 60% 70% 80% 90% 100%

#-4.99 -3.83 -2.62 -1.89 -0.73 -0.37 0.37 1.41 5.24 6.32

#Min. 1st Qu. Median Mean 3rd Qu. Max.

#-9.230 -3.100 -0.730 -0.805 1.010 6.320

# 4. 绘制地图

ggplot(data = world_data) +

geom_sf(aes(fill = EAPC), color = "white", size = 0.2) + # 填充EAPC值

scale_fill_gradientn(

colors = c("#2c7bb6", "#5aa3c9","#70b5d1","#89c6d8" ,"#b3d0c2","#e8c6a1","#d94d4d", "#a50026"),

values = scales::rescale(c(-8,-4,0,4)), # 根据数据范围调整

breaks = c(-8,-4,0,4),

labels = c("-8","-4","0","4"),

na.value = "grey90" ) +

labs(

title = "Country Trends of EAPC (1990-2021)"

) +

theme_minimal() +

theme(

plot.title = element_text(hjust = 0.5, size = 16, face = "bold"),

legend.position = "right",

legend.key.width = unit(0.6, "cm"),

panel.grid = element_blank()

)

# 5. 保存图片（可选）

#ggsave("eapc_world_map.png", width = 12, height = 8, dpi = 300)

#--------top10国家(1)--------------

library(dplyr)

library(ggplot2)

library(scales)

# 读取数据并清洗

eapc <- read.csv("eapc_results1.csv") %>%

filter(EAPC != Inf) # 移除无穷大值

# 定义颜色方案

red_palette <- colorRampPalette(c( "#A50026","#FFD699"))(10) # 红黄渐变

blue_palette <- colorRampPalette(c("#2C7BB6","#ABD9E9"))(10) # 蓝青渐变

# 处理数据

plot_data <- bind_rows(

# Top 10增长国家

eapc %>%

filter(EAPC > 0) %>%

slice_max(EAPC, n = 10) %>%

mutate(color = red_palette,

group = "Increasing"),

# Top 10下降国家

eapc %>%

filter(EAPC < 0) %>%

slice_min(EAPC, n = 10) %>%

mutate(color = blue_palette,

group = "Declining")

) %>%

mutate(label = paste0(round(EAPC, 1), "%"),

group = factor(group, levels = c("Increasing", "Declining")),

location = reorder(location, EAPC))

# 可视化

ggplot(plot_data, aes(EAPC, location, fill = color)) +

geom_col(width = 0.8, show.legend = FALSE) +

geom_text(aes(label = label, x = ifelse(EAPC > 0, -2, 2)),

hjust = "opposite", size = 3) +

scale_fill_identity() +

scale_x_continuous(labels = percent_format(scale = 1)) +

facet_grid(group ~ ., scales = "free_y", space = "free_y") +

labs(title = "Top 10 increasing and declining countries (1990-2021)",

x = "EAPC",

y = NULL) +

theme_minimal(base_size = 12) +

theme(

plot.title = element_text(hjust = 0.5, face = "bold", size = 16),

strip.text = element_text(face = "bold", size = 12),

panel.spacing = unit(1, "lines"),

axis.text.y = element_text(color = "black"),

panel.grid.major.y = element_blank()

)

#

#--------top10国家(2)-----------------

library(dplyr)

library(ggplot2)

library(scales)

# 读取数据并清洗

eapc <- read.csv("eapc_results2.csv") %>%

filter(EAPC != Inf) # 移除无穷大值

# 定义颜色方案

red_palette <- colorRampPalette(c( "#A50026","#FFD699"))(10) # 红黄渐变

blue_palette <- colorRampPalette(c("#2C7BB6","#ABD9E9"))(10) # 蓝青渐变

# 处理数据

plot_data <- bind_rows(

# Top 10增长国家

eapc %>%

filter(EAPC > 0) %>%

slice_max(EAPC, n = 10) %>%

mutate(color = red_palette,

group = "Increasing"),

# Top 10下降国家

eapc %>%

filter(EAPC < 0) %>%

slice_min(EAPC, n = 10) %>%

mutate(color = blue_palette,

group = "Declining")

) %>%

mutate(label = paste0(round(EAPC, 1), "%"),

group = factor(group, levels = c("Increasing", "Declining")),

location = reorder(location, EAPC))

# 可视化

ggplot(plot_data, aes(EAPC, location, fill = color)) +

geom_col(width = 0.8, show.legend = FALSE) +

geom_text(aes(label = label, x = ifelse(EAPC > 0, -0.6, 0.7)),

hjust = "opposite", size = 3) +

scale_fill_identity() +

scale_x_continuous(labels = percent_format(scale = 1)) +

facet_grid(group ~ ., scales = "free_y", space = "free_y") +

labs(title = "Top 10 increasing and declining countries (1990-2021)",

x = "EAPC",

y = NULL) +

theme_minimal(base_size = 12) +

theme(

plot.title = element_text(hjust = 0.5, face = "bold", size = 16),

strip.text = element_text(face = "bold", size = 12),

panel.spacing = unit(1, "lines"),

axis.text.y = element_text(color = "black"),

panel.grid.major.y = element_blank()

)

**PART 10 Decomposition Analysis**

#设置工作空间

setwd("D:/ Decomposition Analysis ")

## Decomposition

library(dplyr)

library(data.table)

library(purrr)

library(tidyr)

library(ggplot2)

library(ggsci)

## 读取疾病数据

Global <- fread('Global.csv')

Global_infections1<-subset(Global,

Global$cause_name =='HIV/AIDS and sexually transmitted infections')

unique(Global_infections1$cause_name)

Global_infections2<-subset(Global,

Global$cause_name =='Other infectious diseases')

unique(Global_infections2$cause_name)

#age1对应IBD——china数据和age2对应总人口数据

age1 <- c("<5 years","5-9 years","10-14 years","15-19 years","20-24 years",

"25-29 years","30-34 years","35-39 years","40-44 years","45-49 years",

"50-54 years","55-59 years","60-64 years","65-69 years","70-74 years",

"75-79 years","80-84 years","85-89 years","90-94 years","95+ years") ###20个年龄组

age2 <- c("<5 years","5-9 years","10-14 years","15-19 years","20-24 years",

"25-29 years","30-34 years","35-39 years","40-44 years","45-49 years",

"50-54 years","55-59 years","60-64 years","65-69 years","70-74 years",

"75-79 years","80-84 years","85-89 years","90-94 years","95+ years") ###20个年龄

var_name <- c("location_name","sex_name","year","age_name","val")

####Global_infections1

## sub函数：将年龄的label,比如"15-19 years"转换成“15 to 19”,以匹配人口学数据和疾病数据

infections1_both<- subset(Global_infections1,

(Global_infections1$age_name %in% age1 ) &

Global_infections1$location_name=='Global')

infections1_both$age_name<-gsub(" years","",infections1_both$age_name)

infections1_both$age_name <- factor(infections1_both$age_name, levels = c("<5", "5-9", "10-14", "15-19",

"20-24", "25-29", "30-34", "35-39", "40-44", "45-49", "50-54",

"55-59", "60-64", "65-69", "70-74", "75-79", "80-84", "85-89",

"90-94", "95+"))

## 读取人口学数据

Global_population <- fread('Global_population.csv')

var_name <- c("location_name","sex_name","year","age_id","age_name","val")

unique(Global_population$age_name)

#Global_population<-Global_population%>% dplyr::select("location_name","sex_name","year","age_name","val") %>%

#filter(Global_population %in% 'Global' & age_name %in% age2)

###

Global_population$age_name<-gsub(" years","",Global_population$age_name)

unique(Global_population$age_name)

Global_population$age_name <- factor(Global_population$age_name, levels = c("<5", "5-9", "10-14", "15-19",

"20-24", "25-29", "30-34", "35-39", "40-44", "45-49", "50-54",

"55-59", "60-64", "65-69", "70-74", "75-79", "80-84", "85-89",

"90-94", "95+"))

Global_population<-subset(Global_population,Global_population$year==1990|Global_population$year==2021)

#str(population)

head(Global_population)

#计算1990年全人群的年龄构成

pop1<-subset(Global_population,Global_population$year==1990 & Global_population$sex_name == "Both")

tot1990<-sum(pop1$val)

pop1$percent <- pop1$val/tot1990

#计算2021年全人群的年龄构成

pop2<-subset(Global_population,Global_population$year==2021 & Global_population$sex_name == "Both")

tot2021<-sum(pop2$val)

pop2$percent <- pop2$val/tot2021

#提取分析所用数据

### a代表年龄占比，p代表总人群数，将数据提取出来

a_1990 <- pop1$percent

a_2021 <- pop2$percent

p_1990 <- tot1990

p_2021 <- tot2021

## 获取率的数据

case_1990 <- infections1_both %>% filter(year == 1990 &

sex_name == "Both" &

location_name == "Global" &

metric_name == 'Rate' &

measure_name == 'DALYs (Disability-Adjusted Life Years)')

case_2021 <- infections1_both %>% filter(year == 2021 &

location_name == 'Global' &

sex_name == "Both" &

metric_name == 'Rate' &

measure_name == 'DALYs (Disability-Adjusted Life Years)')

r_1990 <- as.numeric(case_1990$val)/10^5 ## 单位转换

r_2021 <- as.numeric(case_2021$val)/10^5 ## 单位转换

##### 根据公式计算

a_effect <- round((sum(a_2021*p_1990*r_1990) + sum(a_2021*p_2021*r_2021))/3 +

(sum(a_2021*p_1990*r_2021) + sum(a_2021*p_2021*r_1990))/6 -

(sum(a_1990*p_1990*r_1990) + sum(a_1990*p_2021*r_2021))/3 -

(sum(a_1990*p_1990*r_2021) + sum(a_1990*p_2021*r_1990))/6,3)

p_effect <- round((sum(a_1990*p_2021*r_1990) + sum(a_2021*p_2021*r_2021))/3 +

(sum(a_1990*p_2021*r_2021) + sum(a_2021*p_2021*r_1990))/6 -

(sum(a_1990*p_1990*r_1990) + sum(a_2021*p_1990*r_2021))/3 -

(sum(a_1990*p_1990*r_2021) + sum(a_2021*p_1990*r_1990))/6,3)

r_effect <- round((sum(a_1990*p_1990*r_2021) + sum(a_2021*p_2021*r_2021))/3 +

(sum(a_1990*p_2021*r_2021) + sum(a_2021*p_1990*r_2021))/6 -

(sum(a_1990*p_1990*r_1990) + sum(a_2021*p_2021*r_1990))/3 -

(sum(a_1990*p_2021*r_1990) + sum(a_2021*p_1990*r_1990))/6,3)

overll_differ <- round(a_effect + p_effect + r_effect,2)

# 计算基于绝对值总和的百分比贡献

total_absolute_effect <- abs(a_effect) + abs(p_effect) + abs(r_effect)

if (total_absolute_effect > 0) {

a_percent <- round((a_effect / total_absolute_effect) * 100, 2)

p_percent <- round((p_effect / total_absolute_effect) * 100, 2)

r_percent <- round((r_effect / total_absolute_effect) * 100, 2)

} else {

a_percent <- p_percent <- r_percent <- 0

}

# 验证百分比之和约为100%（考虑方向）

percent_sum <- a_percent + p_percent + r_percent#数据合并，形成一个数据集

temp <- c("Both",overll_differ,a_effect,p_effect,r_effect,a_percent,

p_percent,r_percent) %>% t() %>% as.data.frame()

#生成表头

decomposition_name<-c('sex_name','overll_difference','a_effect','p_effect','r_effect','a_percent',

'p_percent','r_percent')

names(temp) <- decomposition_name

#计算1990年全人群的年龄构成

pop1<-subset(Global_population,Global_population$year==1990 & Global_population$sex_name == "Both")

tot1990<-sum(pop1$val)

pop1$percent <- pop1$val/tot1990

#计算2021年全人群的年龄构成

pop2<-subset(Global_population,Global_population$year==2021 & Global_population$sex_name == "Both")

tot2021<-sum(pop2$val)

pop2$percent <- pop2$val/tot2021

#提取分析所用数据

### a代表年龄占比，p代表总人群数，将数据提取出来

a_1990 <- pop1$percent

a_2021 <- pop2$percent

p_1990 <- tot1990

p_2021 <- tot2021

## 获取率的数据

case_1990 <- IBD_in_both %>% filter(year == 1990 &

sex_name == "Both" &

location_name == "China" &

metric_name == 'Rate' &

measure_name == 'Incidence')

case_2021 <- IBD_in_both %>% filter(year == 2021 &

location_name == 'China' &

sex_name == "Both" &

metric_name == 'Rate' &

measure_name == 'Incidence')

r_1990 <- as.numeric(case_1990$val)/10^5 ## 单位转换

r_2021 <- as.numeric(case_2021$val)/10^5 ## 单位转换

##### 根据公式计算

a_effect <- round((sum(a_2021*p_1990*r_1990) + sum(a_2021*p_2021*r_2021))/3 +

(sum(a_2021*p_1990*r_2021) + sum(a_2021*p_2021*r_1990))/6 -

(sum(a_1990*p_1990*r_1990) + sum(a_1990*p_2021*r_2021))/3 -

(sum(a_1990*p_1990*r_2021) + sum(a_1990*p_2021*r_1990))/6,3)

p_effect <- round((sum(a_1990*p_2021*r_1990) + sum(a_2021*p_2021*r_2021))/3 +

(sum(a_1990*p_2021*r_2021) + sum(a_2021*p_2021*r_1990))/6 -

(sum(a_1990*p_1990*r_1990) + sum(a_2021*p_1990*r_2021))/3 -

(sum(a_1990*p_1990*r_2021) + sum(a_2021*p_1990*r_1990))/6,3)

r_effect <- round((sum(a_1990*p_1990*r_2021) + sum(a_2021*p_2021*r_2021))/3 +

(sum(a_1990*p_2021*r_2021) + sum(a_2021*p_1990*r_2021))/6 -

(sum(a_1990*p_1990*r_1990) + sum(a_2021*p_2021*r_1990))/3 -

(sum(a_1990*p_2021*r_1990) + sum(a_2021*p_1990*r_1990))/6,3)

overll_differ <- round(a_effect + p_effect + r_effect,2)

a_percent <- round(a_effect/overll_differ*100,2)

p_percent <- round(p_effect/overll_differ*100,2)

r_percent <- round(r_effect/overll_differ*100,2)

#数据合并，形成一个数据集

temp <- c("Both",overll_differ,a_effect,p_effect,r_effect,a_percent,

p_percent,r_percent) %>% t() %>% as.data.frame()

#生成表头及分解分析数据集

decomposition_name<-c('sex_name','overll_difference','a_effect','p_effect','r_effect','a_percent',

'p_percent','r_percent')

names(temp) <- decomposition_name

decomposition_data<-temp

# 作图

#数据的整理，宽转长

names(decomposition_data)[2:5] <- c('Overll difference','Aging','Population','Epidemiological change')

decomposition_plot <- decomposition_data[c(1:5)]

decomposition_plot <- decomposition_plot%>%

pivot_longer(3:5, #宽数据转长数据

names_to = "varname",

values_to = "value") %>%

mutate(value=as.numeric(value))

#定义不同组颜色

my_colors <- c("#FFFB73", "#33CCCC", "#FFC773")

#画条形图

p <- ggplot(decomposition_plot, aes(x= sex_name,y=value, fill= varname)) +

geom_bar(stat="identity",position = "stack") +

coord_flip() +

#scale_fill_nejm() +

scale_fill_manual(values = my_colors) +

theme_bw()

p

decomposition_data$`Overll difference` <- as.numeric(decomposition_data$`Overll difference`)

plot <- p+ geom_point(data=decomposition_data, mapping=aes(x=sex_name,y= `Overll difference`),fill='black',color='black',size=3)

plot

# #制作一个空表格

decomposition_name <- c('sex_name','overll_difference','a_effect','p_effect','r_effect','a_percent',

'p_percent','r_percent')

decomposition_data <- as.data.frame(matrix(nrow=0,ncol=length(decomposition_name)))

names(decomposition_data) <- decomposition_name

sex_n <- c("Both","Male","Female")

for (a in sex_n) {

Global_population_1990 <- Global_population %>%

filter(sex_name == a &

year == 1990 &

location_name == 'Global'

)

Global_1990 <- sum(Global_population_1990$val) ## 计算1990人口总数

Global_population_1990$percent <- Global_population_1990$val/Global_1990 ### 获取1990年不同年龄段的占比

## 获取2021年不同年龄段的占比

Global_population_2021 <- Global_population %>%

filter(sex_name == a &

year == 2021 &

location_name == 'Global')

Global_2021 <- sum(Global_population_2021$val) ## 计算2021人口总数

Global_population_2021$percent <- Global_population_2021$val/Global_2021 ### 获取2021年不同年龄段的占比

### a代表年龄占比，p代表总人群数，将数据提取出来

a_1990 <- Global_population_1990$percent

a_2021 <- Global_population_2021$percent

p_1990 <- Global_1990

p_2021 <- Global_2021

## 获取率的数据

case_1990 <- infections1_both %>% filter(year == 1990 &

sex_name == a &

location_name == "Global" &

metric_name == 'Rate' &

measure_name == 'DALYs (Disability-Adjusted Life Years)')

case_2021 <- infections1_both %>% filter(year == 2021 &

location_name == 'Global' &

sex_name == a &

metric_name == 'Rate' &

measure_name == 'DALYs (Disability-Adjusted Life Years)')

r_1990 <- as.numeric(case_1990$val)/10^5 ## 单位转换

r_2021 <- as.numeric(case_2021$val)/10^5 ## 单位转换

# ##### 根据公式计算

a_effect <- round((sum(a_2021*p_1990*r_1990) + sum(a_2021*p_2021*r_2021))/3 +

(sum(a_2021*p_1990*r_2021) + sum(a_2021*p_2021*r_1990))/6 -

(sum(a_1990*p_1990*r_1990) + sum(a_1990*p_2021*r_2021))/3 -

(sum(a_1990*p_1990*r_2021) + sum(a_1990*p_2021*r_1990))/6,3)

p_effect <- round((sum(a_1990*p_2021*r_1990) + sum(a_2021*p_2021*r_2021))/3 +

(sum(a_1990*p_2021*r_2021) + sum(a_2021*p_2021*r_1990))/6 -

(sum(a_1990*p_1990*r_1990) + sum(a_2021*p_1990*r_2021))/3 -

(sum(a_1990*p_1990*r_2021) + sum(a_2021*p_1990*r_1990))/6,3)

r_effect <- round((sum(a_1990*p_1990*r_2021) + sum(a_2021*p_2021*r_2021))/3 +

(sum(a_1990*p_2021*r_2021) + sum(a_2021*p_1990*r_2021))/6 -

(sum(a_1990*p_1990*r_1990) + sum(a_2021*p_2021*r_1990))/3 -

(sum(a_1990*p_2021*r_1990) + sum(a_2021*p_1990*r_1990))/6,3)

overll_differ <- round(a_effect + p_effect + r_effect,2)

a_percent <- round(a_effect/overll_differ*100,2)

p_percent <- round(p_effect/overll_differ*100,2)

r_percent <- round(r_effect/overll_differ*100,2)

temp <- c(a,overll_differ,a_effect,p_effect,r_effect,a_percent,

p_percent,r_percent) %>% t() %>% as.data.frame()

names(temp) <- decomposition_name

decomposition_data <- rbind(decomposition_data,temp)

}

data1 <- read.csv('Global_infections1.csv')

num_1990 <- data1 %>% filter(age_name == 'All ages' &

location_name == 'Global' &

metric_name == 'Number' &

year == 1990 &

measure_name == 'DALYs (Disability-Adjusted Life Years)') %>%

select(sex_name, val) %>%

rename(val_1990 = val)

num_2021 <- data1 %>% filter(age_name == 'All ages' &

location_name == 'Global' &

metric_name == 'Number' &

year == 2021 &

measure_name == 'DALYs (Disability-Adjusted Life Years)') %>%

select(sex_name, val) %>%

rename(val_2021 = val)

decomposition_data <- left_join(decomposition_data,num_1990, by = 'sex_name') %>%

left_join(num_2021, by = 'sex_name')

decomposition_data$diff1 <- decomposition_data$val_2021 - decomposition_data$val_1990

decomposition_data[,2:11] <- decomposition_data[,2:11] %>% apply(c(1,2),as.numeric)

round(decomposition_data$diff) == round(decomposition_data$overll_difference)

write.csv(decomposition_data,"DALYs.csv")

#library(openxlsx)

#decomposition_data <- data.frame(read.xlsx("DALYs.xlsx",sheet = 3) )

## 作图

names(decomposition_data)[2:5] <- c('Overll difference','Aging','Population','Epidemiological change')

decomposition_plot <- decomposition_data[c(1:5)]

decomposition_plot <- decomposition_plot%>%

pivot_longer(3:5,

names_to = "varname",

values_to = "value") %>%

mutate(value=as.numeric(value)) %>%

mutate(sex_name=factor(sex_name,levels = sex_n,ordered = T))

my_colors <- c("#FFFB73", "#33CCCC", "#FFC773")

p <- ggplot(decomposition_plot, aes(x= sex_name,y=value, fill= varname)) +

geom_bar(stat="identity",position = "stack") +

coord_flip() +

scale_fill_nejm() +

scale_fill_manual(values = my_colors) +

theme_bw()

p

decomposition_data$`Overll difference` <- as.numeric(decomposition_data$`Overll difference`)

plot <- p+ geom_point(data=decomposition_data, mapping=aes(x=sex_name,y= `Overll difference`),fill='black',color='black',size=3)

plot

######infactions2

# ####不同性别的分解分析####

## sub函数：将年龄的label,比如"15-19 years"转换成“15 to 19”,以匹配人口学数据和疾病数据

infections2_both<- subset(Global_infections2,

(Global_infections2$age_name %in% age1 ) &

Global_infections2$location_name=='Global')

infections2_both$age_name<-gsub(" years","",infections2_both$age_name)

infections2_both$age_name <- factor(infections2_both$age_name, levels = c("<5", "5-9", "10-14", "15-19",

"20-24", "25-29", "30-34", "35-39", "40-44", "45-49", "50-54",

"55-59", "60-64", "65-69", "70-74", "75-79", "80-84", "85-89",

"90-94", "95+"))

# #制作一个空表格

decomposition_name <- c('sex_name','overll_difference','a_effect','p_effect','r_effect','a_percent',

'p_percent','r_percent')

decomposition_data <- as.data.frame(matrix(nrow=0,ncol=length(decomposition_name)))

names(decomposition_data) <- decomposition_name

sex_n <- c("Both","Male","Female")

for (a in sex_n) {

Global_population_1990 <- Global_population %>%

filter(sex_name == a &

year == 1990 &

location_name == 'Global'

)

Global_1990 <- sum(Global_population_1990$val) ## 计算1990人口总数

Global_population_1990$percent <- Global_population_1990$val/Global_1990 ### 获取1990年不同年龄段的占比

## 获取2021年不同年龄段的占比

Global_population_2021 <- Global_population %>%

filter(sex_name == a &

year == 2021 &

location_name == 'Global')

Global_2021 <- sum(Global_population_2021$val) ## 计算2021人口总数

Global_population_2021$percent <- Global_population_2021$val/Global_2021 ### 获取2021年不同年龄段的占比

### a代表年龄占比，p代表总人群数，将数据提取出来

a_1990 <- Global_population_1990$percent

a_2021 <- Global_population_2021$percent

p_1990 <- Global_1990

p_2021 <- Global_2021

## 获取率的数据

case_1990 <- infections2_both %>% filter(year == 1990 &

sex_name == a &

location_name == "Global" &

metric_name == 'Rate' &

measure_name == 'DALYs (Disability-Adjusted Life Years)')

case_2021 <- infections2_both %>% filter(year == 2021 &

location_name == 'Global' &

sex_name == a &

metric_name == 'Rate' &

measure_name == 'DALYs (Disability-Adjusted Life Years)')

r_1990 <- as.numeric(case_1990$val)/10^5 ## 单位转换

r_2021 <- as.numeric(case_2021$val)/10^5 ## 单位转换

# ##### 根据公式计算

a_effect <- round((sum(a_2021*p_1990*r_1990) + sum(a_2021*p_2021*r_2021))/3 +

(sum(a_2021*p_1990*r_2021) + sum(a_2021*p_2021*r_1990))/6 -

(sum(a_1990*p_1990*r_1990) + sum(a_1990*p_2021*r_2021))/3 -

(sum(a_1990*p_1990*r_2021) + sum(a_1990*p_2021*r_1990))/6,3)

p_effect <- round((sum(a_1990*p_2021*r_1990) + sum(a_2021*p_2021*r_2021))/3 +

(sum(a_1990*p_2021*r_2021) + sum(a_2021*p_2021*r_1990))/6 -

(sum(a_1990*p_1990*r_1990) + sum(a_2021*p_1990*r_2021))/3 -

(sum(a_1990*p_1990*r_2021) + sum(a_2021*p_1990*r_1990))/6,3)

r_effect <- round((sum(a_1990*p_1990*r_2021) + sum(a_2021*p_2021*r_2021))/3 +

(sum(a_1990*p_2021*r_2021) + sum(a_2021*p_1990*r_2021))/6 -

(sum(a_1990*p_1990*r_1990) + sum(a_2021*p_2021*r_1990))/3 -

(sum(a_1990*p_2021*r_1990) + sum(a_2021*p_1990*r_1990))/6,3)

overll_differ <- round(a_effect + p_effect + r_effect,2)

# 计算基于绝对值总和的百分比贡献

total_absolute_effect <- abs(a_effect) + abs(p_effect) + abs(r_effect)

if (total_absolute_effect > 0) {

a_percent <- round((a_effect / total_absolute_effect) * 100, 2)

p_percent <- round((p_effect / total_absolute_effect) * 100, 2)

r_percent <- round((r_effect / total_absolute_effect) * 100, 2)

} else {

a_percent <- p_percent <- r_percent <- 0

}

# 验证百分比之和约为100%（考虑方向）

percent_sum <- a_percent + p_percent + r_percent temp <- c(a,overll_differ,a_effect,p_effect,r_effect,a_percent,

p_percent,r_percent) %>% t() %>% as.data.frame()

names(temp) <- decomposition_name

decomposition_data <- rbind(decomposition_data,temp)

}

data1 <- read.csv('Global_infections2.csv')

num_1990 <- data1 %>% filter(age_name == 'All ages' &

location_name == 'Global' &

metric_name == 'Number' &

year == 1990 &

measure_name == 'DALYs (Disability-Adjusted Life Years)') %>%

select(sex_name, val) %>%

rename(val_1990 = val)

num_2021 <- data1 %>% filter(age_name == 'All ages' &

location_name == 'Global' &

metric_name == 'Number' &

year == 2021 &

measure_name == 'DALYs (Disability-Adjusted Life Years)') %>%

select(sex_name, val) %>%

rename(val_2021 = val)

decomposition_data <- left_join(decomposition_data,num_1990, by = 'sex_name') %>%

left_join(num_2021, by = 'sex_name')

decomposition_data$diff1 <- decomposition_data$val_2021 - decomposition_data$val_1990

decomposition_data[,2:11] <- decomposition_data[,2:11] %>% apply(c(1,2),as.numeric)

round(decomposition_data$diff) == round(decomposition_data$overll_difference)

write.csv(decomposition_data,"DALYs2.csv")

#library(openxlsx)

#decomposition_data <- data.frame(read.xlsx("DALYs.xlsx",sheet = 3) )

## 作图

names(decomposition_data)[2:5] <- c('Overll difference','Aging','Population','Epidemiological change')

decomposition_plot <- decomposition_data[c(1:5)]

decomposition_plot <- decomposition_plot%>%

pivot_longer(3:5,

names_to = "varname",

values_to = "value") %>%

mutate(value=as.numeric(value)) %>%

mutate(sex_name=factor(sex_name,levels = sex_n,ordered = T))

my_colors <- c("#FFFB73", "#33CCCC", "#FFC773")

p <- ggplot(decomposition_plot, aes(x= sex_name,y=value, fill= varname)) +

geom_bar(stat="identity",position = "stack") +

coord_flip() +

scale_fill_nejm() +

scale_fill_manual(values = my_colors) +

theme_bw()

p

decomposition_data$`Overll difference` <- as.numeric(decomposition_data$`Overll difference`)

plot <- p+ geom_point(data=decomposition_data, mapping=aes(x=sex_name,y= `Overll difference`),fill='black',color='black',size=3)

plot

**PART 11 ARIMA**

setwd("D:/ARIMA")

# 安装并加载必要的包

#install.packages("forecast")

#install.packages("readxl")

library(forecast)

library(ggplot2)

library(readxl)

library(ggpubr)

#####ASMR1 导入数据#####

ASMR1<-read_excel("ASMR1.xlsx",sheet=1)

# 将数据转换为时间序列对象

ASMR1_ts <- ts(ASMR1$val, start = 1990, frequency = 1)

# 检查数据

plot(ASMR1_ts)

# 拟合ARIMA模型

fit1 <- auto.arima(ASMR1_ts) ###自动拟合出最佳的p,d,q的值

fit1 ###查看p,d,q的值

summary(fit1) ###查看模型的AIC,BIC的指标

# 进行预测

forecasted_values1 <- forecast(fit1, h = 15) ###预测未来15年数据

# 将预测结果转换为数据框

forecast_df1 <- data.frame(

Year = c(time(ASMR1_ts), time(forecasted_values1$mean)),

Value = c(as.numeric(ASMR1_ts), as.numeric(forecasted_values1$mean)),

Type = c(rep("Actual", length(ASMR1_ts)), rep("Forecast", length(forecasted_values1$mean)))

)

# 绘制预测结果

p1<-ggplot() +

geom_line(data = forecast_df1, aes(x = Year, y = Value, color = Type), size = 1.2) +

geom_line(data = forecast_df1[forecast_df1$Type == "Forecast", ],

aes(x = Year, y = Value, color = Type), size = 1.2) +

geom_point(data = forecast_df1[forecast_df1$Type == "Forecast", ],

aes(x = Year, y = Value, color = Type), size = 2, shape = 21,

fill = "yellow", color = "black", stroke = 0.5) +

geom_ribbon(data = data.frame(

Year = time(forecasted_values1$mean),

ymin = forecasted_values1$lower[,2],

ymax = forecasted_values1$upper[,2]

), aes(x = Year, ymin = ymin, ymax = ymax), fill = "yellow", alpha = 0.2) +

geom_vline(xintercept = 2021, linetype = "dashed", color = "grey", size = 1) +

scale_color_manual(values = c("Actual" = "red", "Forecast" = "yellow")) +

labs(title = "ASMR of Infections1", x = "Year", y = "ASMR") +

ylim(-1.5, 3) +

theme_minimal() +

theme(axis.line = element_line(color = "black"),

plot.title = element_text(hjust = 0, vjust = 1, face = "bold", size = 14),

plot.margin = margin(10, 10, 10, 10))

p1

#####ASMR2导入数据#####

ASMR2<-read_excel("ASMR2.xlsx",sheet=1)

# 将数据转换为时间序列对象

ASMR2_ts <- ts(ASMR2$val, start = 1990, frequency = 1)

# 检查数据

plot(ASMR2_ts)

# 拟合ARIMA模型

fit2 <- auto.arima(ASMR2_ts)

fit2

summary(fit2)

# 进行预测

forecasted_values2 <- forecast(fit2, h = 15)

# 将预测结果转换为数据框

forecast_df2 <- data.frame(

Year = c(time(ASMR2_ts), time(forecasted_values2$mean)),

Value = c(as.numeric(ASMR2_ts), as.numeric(forecasted_values2$mean)),

Type = c(rep("Actual", length(ASMR2_ts)), rep("Forecast", length(forecasted_values2$mean)))

)

# 绘制预测结果

range(forecast_df2$Value, na.rm = TRUE) # 查看y轴实际值范围

p2<-ggplot() +

geom_line(data = forecast_df2, aes(x = Year, y = Value, color = Type), size = 1.2) +

geom_line(data = forecast_df2[forecast_df2$Type == "Forecast", ], aes(x = Year, y = Value, color = Type), size = 1.2) +

geom_point(data = forecast_df2[forecast_df2$Type == "Forecast", ], aes(x = Year, y = Value, color = Type), size = 2, shape = 21, fill = "yellow", color = "black", stroke = 0.5) +

geom_ribbon(data = data.frame(

Year = time(forecasted_values2$mean),

ymin = forecasted_values2$lower[,2],

ymax = forecasted_values2$upper[,2]

), aes(x = Year, ymin = ymin, ymax = ymax), fill = "yellow", alpha = 0.2) +

geom_vline(xintercept = 2021, linetype = "dashed", color = "grey", size = 1) +

scale_color_manual(values = c("Actual" = "red", "Forecast" = "yellow")) +

labs(title = "ASMR of Infections2", x = "Year", y = "ASMR") +

ylim(-0.1, 0.1) +

theme_minimal() +

theme(axis.line = element_line(color = "black"),

plot.title = element_text(hjust = 0, vjust = 1, face = "bold", size = 14),

plot.margin = margin(10, 10, 10, 10))

p2

#####ASDR infaction1 导入数据#####

#library(readxl)

# 读取Excel文件

ASDR1 <- read_excel("ASDR1.xlsx", sheet = 1)

# 将数据转换为时间序列对象

ASDR1_ts <- ts(ASDR1$val, start = 1990, frequency = 1)

# 检查数据

plot(ASDR1_ts)

# 拟合ARIMA模型

fit3 <- auto.arima(ASDR1_ts)

fit3

summary(fit3)

# 进行预测

forecasted_values3 <- forecast(fit3, h = 15)

# 将预测结果转换为数据框

forecast_df3 <- data.frame(

Year = c(time(ASDR1_ts), time(forecasted_values3$mean)),

Value = c(as.numeric(ASDR1_ts), as.numeric(forecasted_values3$mean)),

Type = c(rep("Actual", length(ASDR1_ts)), rep("Forecast", length(forecasted_values3$mean)))

)

# 绘制预测结果

range(forecast_df3$Value, na.rm = TRUE) # 查看y轴实际值范围

p3<-ggplot() +

geom_line(data = forecast_df3, aes(x = Year, y = Value, color = Type), size = 1.2) +

geom_line(data = forecast_df3[forecast_df3$Type == "Forecast", ], aes(x = Year, y = Value, color = Type), size = 1.2) +

geom_point(data = forecast_df3[forecast_df3$Type == "Forecast", ], aes(x = Year, y = Value, color = Type), size = 2, shape = 21, fill = "yellow", color = "black", stroke = 0.5) +

geom_ribbon(data = data.frame(

Year = time(forecasted_values3$mean),

ymin = forecasted_values3$lower[,2],

ymax = forecasted_values3$upper[,2]

), aes(x = Year, ymin = ymin, ymax = ymax), fill = "yellow", alpha = 0.2) +

geom_vline(xintercept = 2021, linetype = "dashed", color = "grey", size = 1) +

scale_color_manual(values = c("Actual" = "red", "Forecast" = "yellow")) +

labs(title = "ASDR of Infections1", x = "Year", y = "ASDR/100 000") +

ylim(-100, 100) +

theme_minimal() +

theme(axis.line = element_line(color = "black"),

plot.title = element_text(hjust = 0, vjust = 1, face = "bold", size = 14),

plot.margin = margin(10, 10, 10, 10))

p3

#####ASDR2 导入数据#####

ASDR2<-read_excel("ASDR2.xlsx",sheet=1)

# 将数据转换为时间序列对象

ASDR2_ts <- ts(ASDR2$val, start = 1990, frequency = 1)

# 检查数据

plot(ASDR2_ts)

# 拟合ARIMA模型

fit4 <- auto.arima(ASDR2_ts)

fit4####查看

summary(fit4)

# 进行预测

forecasted_values4 <- forecast(fit4, h = 15)

# 将预测结果转换为数据框

forecast_df4 <- data.frame(

Year = c(time(ASDR2_ts), time(forecasted_values4$mean)),

Value = c(as.numeric(ASDR2_ts), as.numeric(forecasted_values4$mean)),

Type = c(rep("Actual", length(ASDR2_ts)), rep("Forecast", length(forecasted_values4$mean)))

)

# 绘制预测结果

range(forecast_df4$Value, na.rm = TRUE) # 查看y轴实际值范围

p4<-ggplot() +

geom_line(data = forecast_df4, aes(x = Year, y = Value, color = Type), size = 1.2) +

geom_line(data = forecast_df4[forecast_df4$Type == "Forecast", ], aes(x = Year, y = Value, color = Type), size = 1.2) +

geom_point(data = forecast_df4[forecast_df4$Type == "Forecast", ], aes(x = Year, y = Value, color = Type), size = 2, shape = 21, fill = "yellow", color = "black", stroke = 0.5) +

geom_ribbon(data = data.frame(

Year = time(forecasted_values4$mean),

ymin = forecasted_values4$lower[,2],

ymax = forecasted_values4$upper[,2]

), aes(x = Year, ymin = ymin, ymax = ymax), fill = "yellow", alpha = 0.2) +

geom_vline(xintercept = 2021, linetype = "dashed", color = "grey", size = 1) +

scale_color_manual(values = c("Actual" = "red", "Forecast" = "yellow")) +

labs(title = "ASDR of Infections2", x = "Year", y = "ASDR") +

ylim(-0.3, 3) +

theme_minimal() +

theme(axis.line = element_line(color = "black"),

plot.title = element_text(hjust = 0, vjust = 1, face = "bold", size = 14),

plot.margin = margin(10, 10, 10, 10))

p4

ggarrange(p1,p2,p3,p4,labels = c("A", "B", "C", "D"),

ncol = 2, nrow = 2)

**PART 12 Trends in The All-Age Cases and ASDR, and ASMR of Infectious Diseases by Sex From 1990 to 2021.**

####不同年份疾病负担的变化趋势####

setwd("D:/ ")

library(ggplot2)

library(reshape2)

library(dplyr)

#install.packages('readxl')

library('readxl')

#####第一步 患病率######

#读取数据

IBD_super_region <- read.csv('IBD_super_region.csv',header = T) #事先提取数据

#提取全球传染性疾病数据

Global<-subset(IBD_super_region, ####subset()函数类似excel的筛选功能，用来选数据的子集，也可在Excel完成

IBD_super_region$location_name =='Global' ###全球

)

unique(Global$location_name)

write.csv(Global,"Global.csv")

Global_infections1<-subset(Global,

Global$cause_name =='HIV/AIDS and sexually transmitted infections')

unique(Global_infections1$cause_name)

#提取所需要的数据

data1<- subset(Global_infections1,

Global_infections1$sex_name!="Both"& #提取所有性别

Global_infections1$location_name=='Global'& #提取中国

(Global_infections1$metric_name %in% c('Number','Rate') ) & #提取人数及10万人患病人数

Global_infections1$measure_name =='Deaths') #提取患病率

unique(data1$sex_name)

str(data1)

#筛选所需要的数据

data2 <- data1[,c("sex_name","age_name","metric_name","year","val","upper","lower")]

# 绘制图表

p1 <- ggplot() +

geom_bar(data = subset(data2, age_name =="All ages"),

mapping=aes(x = year, y = val, fill = sex_name),

stat = "identity",

position = position_dodge(width = 0.8),

width = 0.7)+

geom_errorbar(data = subset(data2, age_name =="All ages"),

mapping=aes(x = year, ymin = lower, ymax = upper,

group =sex_name ),

position = position_dodge(width = 0.8),

width = 0.25,

color = "black")+

geom_line(data = subset(data2, age_name =="Age-standardized"),

aes(x = year, y = val*10000, color = sex_name),

size = 1) +

geom_ribbon(data = subset(data2, age_name =="Age-standardized"),

aes(x = year, ymin = lower*10000, ymax = upper*10000,

fill = sex_name),

alpha = 0.2) +

scale_y_continuous(

name = "Deaths Number",

# limits = c(0, 40000),

sec.axis = sec_axis(~ . * 0.0001, name = "Age-standardized Prevalence rate per 100,000")

) +

scale_fill_manual(values = c("#ffaa00", "#1240ab","blue", "red"), name = "Number") +

scale_color_manual(values = c("#ffaa00", "#1240ab"), name = "Rate") +

labs(x = "Year") +

theme_minimal()

print(p1)

#####发生率######

data3<- subset(Global_infections1,

Global_infections1$sex_name!="Both"& #提取所有性别

Global_infections1$location_name=='Global'& #提取中国

(Global_infections1$metric_name %in% c('Number','Rate') ) & #提取人数及10万人患病人数

Global_infections1$measure_name =='DALYs (Disability-Adjusted Life Years)') #发生率

data4 <- data3[,c("sex_name","age_name","metric_name","year","val","upper","lower")]

# 绘制图表

p2 <- ggplot() +

geom_bar(data = subset(data4, age_name =="All ages"),

mapping=aes(x = year, y = val, fill = sex_name),

stat = "identity",

position = position_dodge(width = 0.8),

width = 0.7)+

geom_errorbar(data = subset(data4, age_name =="All ages"),

mapping=aes(x = year, ymin = lower, ymax = upper,

group =sex_name ),

position = position_dodge(width = 0.8),

width = 0.25,

color = "black")+

geom_line(data = subset(data4, age_name =="Age-standardized"),

aes(x = year, y = val*10000, color = sex_name),

size = 1) +

geom_ribbon(data = subset(data4, age_name =="Age-standardized"),

aes(x = year, ymin = lower*10000, ymax = upper*10000,

fill = sex_name),

alpha = 0.2) +

scale_y_continuous(

name = "DALYs Number",

# limits = c(0, 40000),

sec.axis = sec_axis(~ . * 0.0001, name = "Age-standardized Incidence rate per 100,000")

) +

scale_fill_manual(values = c("#ffaa00", "#1240ab","blue", "red"), name = "Number") +

scale_color_manual(values = c("#ffaa00", "#1240ab"), name = "Rate") +

labs(x = "Year") +

theme_minimal()

print(p2)

####Global_infections2 deaths

Global_infections2<-subset(Global,

Global$cause_name =='Other infectious diseases')

unique(Global_infections2$cause_name)

#提取所需要的数据

data1.1<- subset(Global_infections2,

Global_infections2$sex_name!="Both"& #提取所有性别

Global_infections2$location_name=='Global'& #提取中国

(Global_infections2$metric_name %in% c('Number','Rate') ) & #提取人数及10万人患病人数

Global_infections2$measure_name =='Deaths') #提取患病率

unique(data1.1$sex_name)

str(data1.1)

#筛选所需要的数据

data2.2 <- data1.1[,c("sex_name","age_name","metric_name","year","val","upper","lower")]

# 绘制图表

p3 <- ggplot() +

geom_bar(data = subset(data2.2, age_name =="All ages"),

mapping=aes(x = year, y = val, fill = sex_name),

stat = "identity",

position = position_dodge(width = 0.8),

width = 0.7)+

geom_errorbar(data = subset(data2.2, age_name =="All ages"),

mapping=aes(x = year, ymin = lower, ymax = upper,

group =sex_name ),

position = position_dodge(width = 0.8),

width = 0.25,

color = "black")+

geom_line(data = subset(data2.2, age_name =="Age-standardized"),

aes(x = year, y = val*10000, color = sex_name),

size = 1) +

geom_ribbon(data = subset(data2.2, age_name =="Age-standardized"),

aes(x = year, ymin = lower*10000, ymax = upper*10000,

fill = sex_name),

alpha = 0.2) +

scale_y_continuous(

name = "Deaths Number",

# limits = c(0, 40000),

sec.axis = sec_axis(~ . * 0.0001, name = "Age-standardized Prevalence rate per 100,000")

) +

scale_fill_manual(values = c("#ffaa00", "#1240ab","blue", "red"), name = "Number") +

scale_color_manual(values = c("#ffaa00", "#1240ab"), name = "Rate") +

labs(x = "Year") +

theme_minimal()

print(p3)

#####Global_infections2 DALYs######

data3.3<- subset(Global_infections2,

Global_infections2$sex_name!="Both"& #提取所有性别

Global_infections2$location_name=='Global'& #提取中国

(Global_infections2$metric_name %in% c('Number','Rate') ) & #提取人数及10万人患病人数

Global_infections2$measure_name =='DALYs (Disability-Adjusted Life Years)') #发生率

data4.4 <- data3.3[,c("sex_name","age_name","metric_name","year","val","upper","lower")]

# 绘制图表

p4 <- ggplot() +

geom_bar(data = subset(data4.4, age_name =="All ages"),

mapping=aes(x = year, y = val, fill = sex_name),

stat = "identity",

position = position_dodge(width = 0.8),

width = 0.7)+

geom_errorbar(data = subset(data4.4, age_name =="All ages"),

mapping=aes(x = year, ymin = lower, ymax = upper,

group =sex_name ),

position = position_dodge(width = 0.8),

width = 0.25,

color = "black")+

geom_line(data = subset(data4.4, age_name =="Age-standardized"),

aes(x = year, y = val*10000, color = sex_name),

size = 1) +

geom_ribbon(data = subset(data4.4, age_name =="Age-standardized"),

aes(x = year, ymin = lower*10000, ymax = upper*10000,

fill = sex_name),

alpha = 0.2) +

scale_y_continuous(

name = "DALYs Number",

# limits = c(0, 40000),

sec.axis = sec_axis(~ . * 0.0001, name = "Age-standardized Incidence rate per 100,000")

) +

scale_fill_manual(values = c("#ffaa00", "#1240ab","blue", "red"), name = "Number") +

scale_color_manual(values = c("#ffaa00", "#1240ab"), name = "Rate") +

labs(x = "Year") +

theme_minimal()

print(p4)

library(ggpubr)

ggarrange(p1,p2,p3,p4,nrow = 4)

**PART 13 Age-Specific DALYs and Deaths Numbers and ASDR and ASMR of Infectious Diseases in Global, 2021.**

library(ggplot2)

library(reshape2)

library(dplyr)

#install.packages('readxl')

library('readxl')

#设置工作空间，读取数据

setwd("D:/双侧图+折线图")

#读取数据

IBD_super_region <- read.csv('IBD_super_region.csv',header = T) #事先提取数据

#提取全球传染性疾病数据

IBD_Global<-subset(IBD_super_region, ####subset()函数类似excel的筛选功能，用来选数据的子集，也可在Excel完成

IBD_super_region$location_name =='Global' ###全球

)

Global_infections1<-subset(IBD_Global,

IBD_Global$cause_name =='HIV/AIDS and sexually transmitted infections')

unique(Global_infections1$cause_name)

####第一步 不同年龄段患病人数金字塔####

#####分组年龄，一般为5岁一个年龄组

age1 <- c("<5 years","5-9 years","10-14 years","15-19 years","20-24 years",

"25-29 years","30-34 years","35-39 years","40-44 years","45-49 years",

"50-54 years","55-59 years","60-64 years","65-69 years","70-74 years",

"75-79 years","80-84 years","85-89 years","90-94 years","95+ years") ###20个年龄组

#提取患病人数的数据

data1<- subset(Global_infections1,Global_infections1$year==2021& #提取2021年

(Global_infections1$age_name %in% age1 ) & #提取不同年龄段

Global_infections1$sex_name!="Both"& #把男女分开

Global_infections1$location_name=='Global'& #提取中国数据

Global_infections1$metric_name== 'Number' & #提取10人数

Global_infections1$measure_name=='DALYs (Disability-Adjusted Life Years)') #提取患病率数据

str(data1)

#提取分析的数据

data1<-data1[,c("sex_name","age_name","val","upper","lower")]

#年龄数据的处理，替代year 文字

data1$age_name<-gsub(" years","",data1$age_name)

#因子化,按年龄排序

data1$age_name <- factor(data1$age_name, levels = c("<5", "5-9", "10-14", "15-19", "20-24", "25-29", "30-34", "35-39", "40-44", "45-49", "50-54", "55-59", "60-64", "65-69", "70-74", "75-79", "80-84", "85-89", "90-94", "95+"))

# 按照 sex_name 和 age_name 进行排序

data1 <- data1[order(data1$sex_name, data1$age_name),]

#提取整数

data1$val<-round(data1$val,0)

data1$Sex<-as.factor(data1$sex_name)

#设置颜色

custom_colors <- c("Male" = "steelblue", "Female" = "#e31a1c")

p1<-ggplot(data1, aes(x = factor(age_name,levels = unique(age_name)),

y = ifelse(Sex == "Male", val, -val),

fill = Sex)) +

scale_fill_manual(values = custom_colors) +

geom_bar(stat = 'identity')+

coord_flip()+

labs(x = 'Age', y = 'The Numbers of DALYs ')+

geom_text(

aes(label=val, # 显示数值

hjust = ifelse(Sex == "Male", -0.4, 1.1) # 数值的位置

),

size=2)+ ####字体大小

scale_y_continuous(

labels = abs,

expand = expansion(mult = c(0.2, 0.2)) )

p1

##Global_infections2

Global_infections2<-subset(IBD_Global,

IBD_Global$cause_name =='Other infectious diseases')

unique(Global_infections2$cause_name)

####第一步 不同年龄段患病人数金字塔####

#####分组年龄，一般为5岁一个年龄组

age1.1 <- c("<5 years","5-9 years","10-14 years","15-19 years","20-24 years",

"25-29 years","30-34 years","35-39 years","40-44 years","45-49 years",

"50-54 years","55-59 years","60-64 years","65-69 years","70-74 years",

"75-79 years","80-84 years","85-89 years","90-94 years","95+ years") ###20个年龄组

#提取患病人数的数据

data1.1<- subset(Global_infections2,Global_infections2$year==2021& #提取2021年

(Global_infections2$age_name %in% age1.1 ) & #提取不同年龄段

Global_infections2$sex_name!="Both"& #把男女分开

Global_infections2$location_name=='Global'& #提取中国数据

Global_infections2$metric_name== 'Number' & #提取10人数

Global_infections2$measure_name=='DALYs (Disability-Adjusted Life Years)') #提取患病率数据

str(data1.1)

#提取分析的数据

data1.1<-data1.1[,c("sex_name","age_name","val","upper","lower")]

#年龄数据的处理，替代year 文字

data1.1$age_name<-gsub(" years","",data1.1$age_name)

####补充几行空白数据

add <- data.frame(sex_name = c("Male","Female","Male","Female","Male","Female"),

age_name = c("<5","<5","5-9","5-9","10-14","10-14"),

val = c("0","0","0","0","0","0"),

upper = c("0","0","0","0","0","0"),

lower = c("0","0","0","0","0","0"))

add$val <- as.numeric(add$val)

add$upper <- as.numeric(add$upper)

add$lower <- as.numeric(add$lower)

data1.1_combined <- rbind(data1.1, add)

#因子化,按年龄排序

data1.1_combined$age_name <- factor(data1.1_combined$age_name, levels = c("<5", "5-9", "10-14", "15-19", "20-24", "25-29", "30-34", "35-39", "40-44", "45-49", "50-54", "55-59", "60-64", "65-69", "70-74", "75-79", "80-84", "85-89", "90-94", "95+"))

# 按照 sex_name 和 age_name 进行排序

data1.1_combined <- data1.1_combined[order(data1.1_combined$sex_name, data1.1_combined$age_name),]

#提取整数

data1.1_combined$val<-round(data1.1_combined$val,0)

data1.1_combined$Sex<-as.factor(data1.1_combined$sex_name)

#设置颜色

custom_colors <- c("Male" = "steelblue", "Female" = "#e31a1c")

p1.1<-ggplot(data1.1_combined, aes(x = factor(age_name,levels = unique(age_name)),

y = ifelse(Sex == "Male", val, -val),

fill = Sex)) +

scale_fill_manual(values = custom_colors) +

geom_bar(stat = 'identity')+

coord_flip()+

labs(x = 'Age', y = 'The Numbers of DALYs ')+

geom_text(

aes(label=val, # 显示数值

hjust = ifelse(Sex == "Male", -0.4, 1.1) # 数值的位置

),

size=2)+ ####字体大小

scale_y_continuous(

labels = abs,

expand = expansion(mult = c(0.2, 0.2)) )

p1.1

####第二步 不同年龄段deaths人数金字塔####

data2<- subset(Global_infections1,Global_infections1$year==2021&

(Global_infections1$age_name %in% age1 ) &

Global_infections1$sex_name!="Both"&

Global_infections1$location_name=='Global'&

Global_infections1$metric_name== 'Number' &

Global_infections1$measure_name=='Deaths')

#提取分析的数据

data2<-data2[,c("sex_name","age_name","val","upper","lower")]

data2$age_name<-gsub(" years","",data2$age_name)

data2$age_name <- factor(data2$age_name, levels = c("<5", "5-9", "10-14", "15-19", "20-24", "25-29", "30-34", "35-39", "40-44", "45-49", "50-54", "55-59", "60-64", "65-69", "70-74", "75-79", "80-84", "85-89", "90-94", "95+"))

# 按照 sex_name 和 age_name 进行排序

data2 <- data2[order(data2$sex_name, data2$age_name),]

data2$val<-round(data2$val,0)

data2$sex_name<-as.factor(data2$sex_name)

custom_colors <- c("Male" = "#1f78b4", "Female" = "#e31a1c")

data2$Sex<-data2$sex_name

p2<-ggplot(data2, aes(x = factor(age_name,levels = unique(age_name)),

y = ifelse(Sex == "Male", val, -val),

fill = Sex)) +

scale_fill_manual(values = custom_colors) +

geom_bar(stat = 'identity')+

coord_flip()+

labs(x = 'Age', y = 'The Numbers of Deaths ')+

geom_text(

aes(label=val, # 显示数值

hjust = ifelse(Sex == "Male", -0.4, 1.1) # 数值的位置

),

size=2)+ ####字体大小

scale_y_continuous(

labels = abs,

expand = expansion(mult = c(0.2, 0.2)) )

p2

##Global_infections2 deaths

Global_infections2<-subset(IBD_Global,

IBD_Global$cause_name =='Other infectious diseases')

unique(Global_infections2$cause_name)

####第一步 不同年龄段患病人数金字塔####

#####分组年龄，一般为5岁一个年龄组

age1.1 <- c("<5 years","5-9 years","10-14 years","15-19 years","20-24 years",

"25-29 years","30-34 years","35-39 years","40-44 years","45-49 years",

"50-54 years","55-59 years","60-64 years","65-69 years","70-74 years",

"75-79 years","80-84 years","85-89 years","90-94 years","95+ years") ###20个年龄组

#提取患病人数的数据

data2.1<- subset(Global_infections2,Global_infections2$year==2021& #提取2021年

(Global_infections2$age_name %in% age1.1 ) & #提取不同年龄段

Global_infections2$sex_name!="Both"& #把男女分开

Global_infections2$location_name=='Global'& #提取中国数据

Global_infections2$metric_name== 'Number' & #提取10人数

Global_infections2$measure_name=='Deaths') #提取患病率数据

#提取分析的数据

data2.1<-data2.1[,c("sex_name","age_name","val","upper","lower")]

#年龄数据的处理，替代year 文字

data2.1$age_name<-gsub(" years","",data2.1$age_name)

####补充几行空白数据

add <- data.frame(sex_name = c("Male","Female","Male","Female","Male","Female"),

age_name = c("<5","<5","5-9","5-9","10-14","10-14"),

val = c("0","0","0","0","0","0"),

upper = c("0","0","0","0","0","0"),

lower = c("0","0","0","0","0","0"))

add$val <- as.numeric(add$val)

add$upper <- as.numeric(add$upper)

add$lower <- as.numeric(add$lower)

data2.1_combined <- rbind(data2.1, add)

#因子化,按年龄排序

data2.1_combined$age_name <- factor(data2.1_combined$age_name, levels = c("<5", "5-9", "10-14", "15-19", "20-24", "25-29", "30-34", "35-39", "40-44", "45-49", "50-54", "55-59", "60-64", "65-69", "70-74", "75-79", "80-84", "85-89", "90-94", "95+"))

# 按照 sex_name 和 age_name 进行排序

data2.1_combined <- data2.1_combined[order(data2.1_combined$sex_name, data2.1_combined$age_name),]

#提取整数

data2.1_combined$val<-round(data2.1_combined$val,0)

data2.1_combined$Sex<-as.factor(data2.1_combined$sex_name)

#设置颜色

custom_colors <- c("Male" = "steelblue", "Female" = "#e31a1c")

p2.1<-ggplot(data2.1_combined, aes(x = factor(age_name,levels = unique(age_name)),

y = ifelse(Sex == "Male", val, -val),

fill = Sex)) +

scale_fill_manual(values = custom_colors) +

geom_bar(stat = 'identity')+

coord_flip()+

labs(x = 'Age', y = 'The Numbers of DALYs ')+

geom_text(

aes(label=val, # 显示数值

hjust = ifelse(Sex == "Male", -0.4, 1.1) # 数值的位置

),

size=2)+ ####字体大小

scale_y_continuous(

labels = abs,

expand = expansion(mult = c(0.2, 0.2)) )

p2.1

library(ggpubr)

ggarrange(p1,p2,ncol = 1)

**PART 14 Line Chart by Age Group**

####年龄段折线图####

library(ggplot2)

library(reshape2)

library(dplyr)

library(readxl)

#设置工作空间，读取数据

setwd("D:/双侧图+折线图")

#读取数据

IBD_super_region <- read.csv('IBD_super_region.csv',header = T) #事先提取数据

#提取全球传染性疾病数据

IBD_Global<-subset(IBD_super_region, ####subset()函数类似excel的筛选功能，用来选数据的子集，也可在Excel完成

IBD_super_region$location_name =='Global' ###全球

)

unique(IBD_Global$age_name)

Global_infections1<-subset(IBD_Global,

IBD_Global$cause_name =='HIV/AIDS and sexually transmitted infections')

unique(Global_infections1$location_name)

####第一步 不同年龄段患病率折线图####

#分组年龄，一般为5岁一个年龄组

age1 <- c("<5 years","5-9 years","10-14 years","15-19 years","20-24 years",

"25-29 years","30-34 years","35-39 years","40-44 years","45-49 years",

"50-54 years","55-59 years","60-64 years","65-69 years","70-74 years",

"75-79 years","80-84 years","85-89 years","90-94 years","95+ years") ###20个年龄组

#提取患病率的数据

data1<- subset(Global_infections1,Global_infections1$year==2021& #提取2021年

(Global_infections1$age_name %in% age1 ) & #提取不同年龄段

Global_infections1$sex_name!="Both"& #把男女分开

Global_infections1$location_name=='Global'& #提取中国数据

Global_infections1$metric_name== 'Rate' & #提取10人患病人数

Global_infections1$measure_name=='DALYs (Disability-Adjusted Life Years)') #提取患病率数据

str(data1)

#提取分析的数据

data1<-data1[,c("sex_name","age_name","val","upper","lower")]

#年龄数据的处理，替代year 文字

data1$age_name<-gsub(" years","",data1$age_name)

#因子化,按年龄排序

data1$age_name <- factor(data1$age_name, levels = c("<5", "5-9", "10-14", "15-19", "20-24", "25-29", "30-34", "35-39", "40-44", "45-49", "50-54", "55-59", "60-64", "65-69", "70-74", "75-79", "80-84", "85-89", "90-94", "95+"))

# 按照 sex_name 和 age_name 进行排序

data1 <- data1[order(data1$sex_name, data1$age_name),]

#取小2位数点

data1$val<-round(data1$val,2)

#性别进行因子化

data1$Sex<-as.factor(data1$sex_name)

#画不同年龄段折线图

p3 <- ggplot(data = data1,aes(x=data1$age_name,y=data1$val,color=Sex, group = Sex))+

geom_line()+

geom_point(size=1)+

labs(x = 'Age', y = 'The Rate of DALYs ')+

scale_fill_manual(values=c("steelblue","#e31a1c"))+

scale_color_manual(values=c("steelblue","#e31a1c"))+

theme(axis.text.x = element_text(angle = 45,hjust = 1))+

geom_ribbon(aes(ymin=data1$lower,ymax=data1$upper,fill=Sex),alpha=0.1,color=NA)

p3

###infections2

Global_infections2<-subset(IBD_Global,

IBD_Global$cause_name =='Other infectious diseases')

unique(Global_infections2$age_name)

####第一步 不同年龄段患病率折线图####

#分组年龄，一般为5岁一个年龄组

age1.1 <- c("<5 years","5-9 years","10-14 years","15-19 years","20-24 years",

"25-29 years","30-34 years","35-39 years","40-44 years","45-49 years",

"50-54 years","55-59 years","60-64 years","65-69 years","70-74 years",

"75-79 years","80-84 years","85-89 years","90-94 years","95+ years") ###20个年龄组

#提取患病率的数据

data1.1<- subset(Global_infections2,Global_infections2$year==2021& #提取2021年

(Global_infections2$age_name %in% age1.1 ) & #提取不同年龄段

Global_infections2$sex_name!="Both"& #把男女分开

Global_infections2$location_name=='Global'& #提取中国数据

Global_infections2$metric_name== 'Rate' & #提取10人患病人数

Global_infections2$measure_name=='DALYs (Disability-Adjusted Life Years)') #提取患病率数据

str(data1.1)

#提取分析的数据

data1.1<-data1.1[,c("sex_name","age_name","val","upper","lower")]

####补充几行空白数据

add <- data.frame(sex_name = c("Male","Female","Male","Female","Male","Female"),

age_name = c("<5","<5","5-9","5-9","10-14","10-14"),

val = c("0","0","0","0","0","0"),

upper = c("0","0","0","0","0","0"),

lower = c("0","0","0","0","0","0"))

add$val <- as.numeric(add$val)

add$upper <- as.numeric(add$upper)

add$lower <- as.numeric(add$lower)

data1.1_combined <- rbind(data1.1, add)

#年龄数据的处理，替代year 文字

data1.1_combined$age_name<-gsub(" years","",data1.1_combined$age_name)

#因子化,按年龄排序

data1.1_combined$age_name <- factor(data1.1_combined$age_name, levels = c("<5", "5-9", "10-14", "15-19", "20-24", "25-29", "30-34", "35-39", "40-44", "45-49", "50-54", "55-59", "60-64", "65-69", "70-74", "75-79", "80-84", "85-89", "90-94", "95+"))

# 按照 sex_name 和 age_name 进行排序

data1.1_combined <- data1.1_combined[order(data1.1_combined$sex_name, data1.1_combined$age_name),]

#取小2位数点

data1.1_combined$val<-round(data1.1_combined$val,2)

#性别进行因子化

data1.1_combined$Sex<-as.factor(data1.1_combined$sex_name)

#画不同年龄段折线图

p3 <- ggplot(data = data1.1_combined,aes(x=data1.1_combined$age_name,y=data1.1_combined$val,color=Sex, group = Sex))+

geom_line()+

geom_point(size=1)+

labs(x = 'Age', y = 'The Rate of DALYs ')+

scale_fill_manual(values=c("steelblue","#e31a1c"))+

scale_color_manual(values=c("steelblue","#e31a1c"))+

theme(axis.text.x = element_text(angle = 45,hjust = 1))+

geom_ribbon(aes(ymin=data1.1_combined$lower,ymax=data1.1_combined$upper,fill=Sex),alpha=0.1,color=NA)

p3

####第二步 不同年龄段deaths率折线图####

data2<- subset(Global_infections1,Global_infections1$year==2021& #提取2021年

(Global_infections1$age_name %in% age1 ) & #提取不同年龄段

Global_infections1$sex_name!="Both"& #把男女分开

Global_infections1$location_name=='Global'& #提取中国数据

Global_infections1$metric_name== 'Rate' & #提取10人患病人数

Global_infections1$measure_name=='Deaths') #提取deaths率数据

#提取分析的数据

data2<-data2[,c("sex_name","age_name","val","upper","lower")]

data2$age_name<-gsub(" years","",data2$age_name)

data2$age_name <- factor(data2$age_name, levels = c("<5", "5-9", "10-14", "15-19", "20-24", "25-29", "30-34", "35-39", "40-44", "45-49", "50-54", "55-59", "60-64", "65-69", "70-74", "75-79", "80-84", "85-89", "90-94", "95+"))

# 按照 sex_name 和 age_name 进行排序

data2 <- data2[order(data2$sex_name, data2$age_name),]

data2$val<-round(data2$val,2)

data2$Sex<-as.factor(data2$sex_name)

p4 <- ggplot(data = data2,aes(x=data2$age_name,y=data2$val,color=Sex, group = Sex))+

geom_line()+

labs(x = 'Age', y = 'The Rate of Deaths')+

scale_fill_manual(values=c("steelblue","#e31a1c"))+

scale_color_manual(values=c("steelblue","#e31a1c"))+

theme(axis.text.x = element_text(angle = 45,hjust = 1))+

geom_point(size=1.2)+

geom_ribbon(aes(ymin=data2$lower,ymax=data2$upper,fill=Sex),alpha=0.1,color=NA)

p4

###年龄折线图Global infactions2 Deaths

data2.1<- subset(Global_infections2,Global_infections2$year==2021& #提取2021年

(Global_infections2$age_name %in% age1.1 ) & #提取不同年龄段

Global_infections2$sex_name!="Both"& #把男女分开

Global_infections2$location_name=='Global'& #提取数据

Global_infections2$metric_name== 'Rate' & #提取10人患病人数

Global_infections2$measure_name=='Deaths') #提取deaths率数据

#提取分析的数据

data2.1<-data2.1[,c("sex_name","age_name","val","upper","lower")]

####补充几行空白数据

add <- data.frame(sex_name = c("Male","Female","Male","Female","Male","Female"),

age_name = c("<5","<5","5-9","5-9","10-14","10-14"),

val = c("0","0","0","0","0","0"),

upper = c("0","0","0","0","0","0"),

lower = c("0","0","0","0","0","0"))

add$val <- as.numeric(add$val)

add$upper <- as.numeric(add$upper)

add$lower <- as.numeric(add$lower)

data2.1_combined <- rbind(data2.1, add)

data2.1_combined$age_name<-gsub(" years","",data2.1_combined$age_name)

data2.1_combined$age_name <- factor(data2.1_combined$age_name, levels = c("<5", "5-9", "10-14", "15-19", "20-24", "25-29", "30-34", "35-39", "40-44", "45-49", "50-54", "55-59", "60-64", "65-69", "70-74", "75-79", "80-84", "85-89", "90-94", "95+"))

# 按照 sex_name 和 age_name 进行排序

data2.1_combined <- data2.1_combined[order(data2.1_combined$sex_name, data2.1_combined$age_name),]

data2.1_combined$val<-round(data2.1_combined$val,2)

data2.1_combined$Sex<-as.factor(data2.1_combined$sex_name)

p4 <- ggplot(data = data2.1_combined,aes(x=data2.1_combined$age_name,y=data2.1_combined$val,color=Sex, group = Sex))+

geom_line()+

labs(x = 'Age', y = 'The Rate of Deaths')+

scale_fill_manual(values=c("steelblue","#e31a1c"))+

scale_color_manual(values=c("steelblue","#e31a1c"))+

theme(axis.text.x = element_text(angle = 45,hjust = 1))+

geom_point(size=1.2)+

geom_ribbon(aes(ymin=data2.1_combined$lower,ymax=data2.1_combined$upper,fill=Sex),alpha=0.1,color=NA)

p4

####不同图形排列####

library(ggpubr)

ggarrange(p3,p4,ncol = 2)

ggarrange(p1,p3,p2,p4,ncol = 2,nrow=2)

**PART 15 Health Inequality Analysis**

#设置工作空间

setwd("D:/课程材料/代码及实操/实操/09 健康不平等分析")

#install.packages("ggbrace")

# 加载需要的包 ------------------------------------------------------------------

library(tidyverse)

library(data.table)

library(car)# 异方差诊断

library(MASS)# 稳健回归

library(mgcv)# 提供洛伦兹曲线拟合 (样条函数等)

library(splines)# 拟合样条函数

library(broom)

library(ggplot2)

# 数据准备--------------------------------------------------------------------

# 读取并合并三个数据

# 疾病负担数据

data1 <- read.csv("204countryDALYs.csv",header = T,check.names = F)

unique(data1$metric_name)

#########infections1###-----------------

data2 <- subset(data1,data1$measure_name=="DALYs (Disability-Adjusted Life Years)"&

data1$sex_name=="Both"&

data1$age_name=="All ages"&

data1$cause_name=="HIV/AIDS and sexually transmitted infections"&

(data1$year==1990|data1$year==2021))

# sdi 数据

sdi <- read.csv("SDI_2021.csv",header = T,check.names = F)

sdi <- sdi %>% # 宽数据转为长数据

pivot_longer(cols = -Location,names_to = "year") %>%

rename(sdi = value)%>%

dplyr::select(Location,year,sdi)

sdi$year <- as.integer(sdi$year)

names(sdi)[1]<-"location_name"

# 匹配 sdi 与疾病负担, 生成 data

data3 <- left_join(data2,sdi,by=c("location_name","year"))

# 读取人口数据

path <- "GBD_population"

fileName <- list.files(path, pattern = "\\.csv$", full.names = FALSE) # 只匹配csv文件

# 初始化空数据框

population <- data.frame()

# 循环读取并合并

for (k in 1:length(fileName)) {

file_path <- file.path(path, fileName[k])

if (file.exists(file_path)) { # 检查文件是否存在

data <- read.csv(file_path, header = TRUE, stringsAsFactors = FALSE)

population <- rbind(population, data)

} else {

warning(paste("文件不存在:", file_path))

}

}

pop1 <- population %>%

dplyr::select(location_name,sex_name,age_name,year,metric_name,val)

unique(pop1$age_name)

pop1 <- pop1 %>%

filter(age_name=="All ages") %>%

dplyr::select("location_name","sex_name","year","val") %>%

rename(pop=val)

# 合并人口数据, 生成 mydata

mydata <- left_join(data3,pop1,

by=c("location_name","sex_name","year"))

# 斜度指数的可视化 ----------------------------------------------------------------

## 1.绘图数据的准备 -----------------------------------------------------------------

####### 计算总人口---------------

# 检查 pop 列的缺失值数量

sum(is.na(mydata$pop))

# 查看包含 NA 的行

mydata %>% filter(is.na(pop))

#用默认值填充缺失值（如0）

mydata <- mydata %>%

mutate(pop = ifelse(is.na(pop), 0, pop))

a <- mydata %>%

filter(metric_name=="Number") %>%

group_by(year) %>%

summarise(sum=sum(pop))

pop1990 <- a$sum[1]

pop2021 <- a$sum[2]

# 计算加权次序

rank <- mydata %>%

mutate(pop_global=ifelse(year==1990,pop1990,pop2021)) %>%

group_by(year,metric_name) %>%

arrange(sdi) %>%

mutate(cummu=cumsum(pop)) %>% # 计算累积人口

mutate(half=pop/2) %>% # 计算该国家人口的一半

mutate(midpoint=cummu-half) %>% # 累积人口减去该国家人口一半即为人口中点

mutate(weighted_order=midpoint/pop_global) # 人口中点与总人口相比即为改国的相对位置

# 把年份设置为 factor

rank$year <- factor(rank$year)

# 选择数据

temp1 <- rank %>%

filter(metric_name=="Rate") %>%

filter(year==1990)

temp2 <- rank %>%

filter(metric_name=="Rate") %>%

filter(year==2021)

# 建模计算斜度指数

fit1 <- lm(data = temp1,val~weighted_order)

fit2 <- lm(data = temp2,val~weighted_order)

coef(fit1)

coef(fit2)

# 查看是否存在异方差（存在异方差）

ncvTest(fit1)

ncvTest(fit2)

# 使用稳健（robust）回归：重复迭代加权

r.huber1 <- rlm(data = temp1,val~weighted_order)

r.huber2 <- rlm(data = temp2,val~weighted_order)

# 获得系数与截距

coef(r.huber1)

coef(r.huber2)

# 计算稳健回归的 95% 可信区间

confint.default(r.huber1) ####这里weighted_order的系数即为斜率指数

confint.default(r.huber2) ####这里weighted_order的系数即为斜率指数

library(ggpubr)

# 2.绘图 ----------------------------------------------------------------------

color <- c("#6699FF","#990000")

unique(rank$location_name)

colnames(rank)

rank <- rank %>%

mutate(location_name = case_when(

location_name == "United States of America" ~ "USA",

location_name == "Russian Federation" ~ "Russia",

location_name == "People's Republic of China" ~ "China",

TRUE ~ location_name # 保留其他国家的名称不变

))

#rank <- rank %>%

# mutate(

# cause_num = case_when(

# cause_name == "HIV/AIDS and sexually transmitted infections" ~ "1",

# cause_name == "Other infectious diseases" ~ "2",

# TRUE ~ "" # 其他病因留空（根据需求调整）

# ),

# 合并国家名称和病因编号标签（如 "USA 1"）

# label_text = paste(location_name, cause_num)

# )

p1 <- rank %>%

filter(metric_name=="Rate") %>%

ggplot(aes(x=weighted_order,y=val,fill=year,group=year,color=year))+

geom_point(aes(color=year,size=pop/1e6),alpha=0.8,shape=21)+

scale_size_area("Population\n(million)",breaks=c(50,100,150,200,250,300))+

geom_smooth(method = "rlm",size=0.6,alpha=0.1)+

scale_fill_manual(values = color)+

scale_color_manual(values = color)+

#增加水平虚线

geom_segment(x=0.02,xend=0.99,

y=9.453022,yend=9.453022, # coef(fit1)或coef(r.huber1)截距的位置

color="#6699FF",linetype=2,size=0.4,alpha=0.4)+

geom_segment(x=0.02,xend=0.99,

y=16.785257,yend=16.785257, # coef(r.huber2)截距的位置

color="#990000",linetype=2,size=0.4,alpha=0.4)+

# 增加某些国家的标签: 比如中国与印度

geom_text(aes(label=ifelse(location_name=="Russia"|location_name=="China"|location_name=="USA",as.character(location_name),""),

color=year),

hjust=0.8,vjust=2,# 避免点和文字重合

size=3)+

# # 增加斜度指数标签

annotate("text",label="Slope Index of Inequality",x=8.628479,y=-1.351180,size=4,angle=90)+

annotate("text",label="-1.351180",x=1.08,y=9.453022+1.351180/2,size=3.5)+ # coef(r.huber1) 权重的系数即斜率指数

annotate("text",label="8.628479",x=1.08,y=19,size=3.5)+ ##coef(r.huber2) 权重的系数即斜率指数

scale_x_continuous(limits = c(0,1.22),labels = c("0","0.25","0.50","0.75","1.00",""))+

xlab("Relative rank by SDI")+

ylab("Crude DALY rate (per 100,000)")+

theme_bw()

p1

# 集中指数的可视化 ----------------------------------------------------------------

# 1.绘图数据准备 ------------------------------------------------------------------

a <- mydata %>%

filter(metric_name=="Number") %>%

group_by(year) %>%

summarise(sum=sum(val))

daly1990 <- a$sum[1]

daly2021 <- a$sum[2]

ci <- rank %>%

filter(metric_name=="Number") %>%

mutate(total_daly=ifelse(year==1990,daly1990,daly2021)) %>%

group_by(year) %>%

arrange(sdi) %>%

mutate(cummu_daly=cumsum(val)) %>% # 计算累积 daly

mutate(frac_daly=cummu_daly/total_daly) %>% # 计算累积 daly 所占总体的比例

mutate(frac_population=cummu/pop_global) # 计算累积人口所占总体人口的比例

#####计算ci

# 选择数据

temp3 <- ci %>%

filter(metric_name=="Number") %>%

filter(year==1990)

temp4 <- ci %>%

filter(metric_name=="Number") %>%

filter(year==2021)

##计算集中指数

CI_1990 <- 2 * (sum(temp3$frac_daly) / nrow(temp3)) - 1

CI_2021 <- 2 * (sum(temp4$frac_daly) / nrow(temp4)) - 1

# 2.绘图 --------------------------------------------------------------------

#调整国家标签名称

ci <- ci %>%

mutate(location_name = case_when(

location_name == "United States of America" ~ "USA",

location_name == "Russian Federation" ~ "Russia",

location_name == "People's Republic of China" ~ "China",

TRUE ~ location_name # 保留其他国家的名称不变

))

p2 <- ci %>%

ggplot(aes(x=frac_population,y=frac_daly,fill=year,color=year,group=year))+

# 增加 X=0,y=0 两条线段

geom_segment(x=0,xend=1,

y=0,yend=0,

linetype=1,size=1,color="gray")+

geom_segment(x=1,xend=1,

y=0,yend=1,

linetype=1,size=1,color="gray")+

# 对角线

geom_segment(x=0,xend=1,

y=0,yend=1,

color="#CD853F",linetype=1,size=0.7,alpha=1)+

# 散点

geom_point(aes(fill=year,size=pop/1e6),alpha=0.75,shape=21)+

scale_fill_manual(values = color)+

scale_size_area("Population\n(million)",breaks=c(50,100,150,200,250,300))+

# 立方样条函数拟合洛伦兹曲线 (设置节点，边界条件)

geom_smooth(method = "gam", # 这里也可以直接用 geom_line 把点连起来

formula = y ~ ns(x,

knots = c(0.0000000001,0.25,0.5,0.75,0.9999999),# 设置节点为

Boundary.knots = c(0,1)),

linetype=1,size=0.1,alpha=0.6,se=T)+

scale_color_manual(values = color)+

# 增加两个年份的集中指数

annotate("text",label="Concentration Index",x=0.75,y=0.35,size=5)+

annotate("text",label="1990: 0.01",x=0.75,y=0.3,size=4,color="#6699FF")+

annotate("text",label="2021: 0.10",x=0.75,y=0.25,size=4,color="#990000")+

# 增加某些国家的标签，1990 年

# 步骤1：在数据中创建简化的病因编号列

geom_text(aes(label=ifelse(location_name=="USA"&year=="1990"|location_name=="Russia"&year=="1990"|location_name=="China"&year=="1990",

as.character(location_name),"")),

hjust=0.8,vjust=-1.4,

size=3)+

#增加某些国家的标签，2021 年

geom_text(aes(label=ifelse(location_name=="USA"&year=="2021"|location_name=="Russia"&year=="2021"|location_name=="China"&year=="2021",

as.character(location_name),"")),

hjust=0.8,vjust=-1.4,

size=3)+

#增加某些国家标签，人口

geom_text(aes(label=ifelse(location_name%in%a&year=="1990",

as.character(location_name),"")),

hjust=0.8,vjust=-1.4,

size=3)+

geom_text(aes(label=ifelse(location_name%in%a&year=="2021",

as.character(location_name),"")),

hjust=0.8,vjust=-1.4,

size=3)+

# xy 标签

xlab("Cumulative fraction of population ranked by SDI")+

ylab("Cumulative fraction of DALY")+

theme_bw()

p2

#########infections2###-----------------

data2 <- subset(data1,data1$measure_name=="DALYs (Disability-Adjusted Life Years)"&

data1$sex_name=="Both"&

data1$age_name=="All ages"&

data1$cause_name=="Other infectious diseases"&

(data1$year==1990|data1$year==2021))

# sdi 数据

sdi <- read.csv("SDI_2021.csv",header = T,check.names = F)

sdi <- sdi %>% # 宽数据转为长数据

pivot_longer(cols = -Location,names_to = "year") %>%

rename(sdi = value)%>%

dplyr::select(Location,year,sdi)

sdi$year <- as.integer(sdi$year)

names(sdi)[1]<-"location_name"

# 匹配 sdi 与疾病负担, 生成 data

data3 <- left_join(data2,sdi,by=c("location_name","year"))

# 读取人口数据

path <- "GBD_population"

fileName <- list.files(path, pattern = "\\.csv$", full.names = FALSE) # 只匹配csv文件

# 初始化空数据框

population <- data.frame()

# 循环读取并合并

for (k in 1:length(fileName)) {

file_path <- file.path(path, fileName[k])

if (file.exists(file_path)) { # 检查文件是否存在

data <- read.csv(file_path, header = TRUE, stringsAsFactors = FALSE)

population <- rbind(population, data)

} else {

warning(paste("文件不存在:", file_path))

}

}

pop1 <- population %>%

dplyr::select(location_name,sex_name,age_name,year,metric_name,val)

unique(pop1$age_name)

pop1 <- pop1 %>%

filter(age_name=="All ages") %>%

dplyr::select("location_name","sex_name","year","val") %>%

rename(pop=val)

# 合并人口数据, 生成 mydata

mydata <- left_join(data3,pop1,

by=c("location_name","sex_name","year"))

# 斜度指数的可视化 ----------------------------------------------------------------

## 1.绘图数据的准备 -----------------------------------------------------------------

####### 计算总人口---------------

# 检查 pop 列的缺失值数量

sum(is.na(mydata$pop))

# 查看包含 NA 的行

mydata %>% filter(is.na(pop))

#用默认值填充缺失值（如0）

mydata <- mydata %>%

mutate(pop = ifelse(is.na(pop), 0, pop))

a <- mydata %>%

filter(metric_name=="Number") %>%

group_by(year) %>%

summarise(sum=sum(pop))

pop1990 <- a$sum[1]

pop2021 <- a$sum[2]

# 计算加权次序

rank <- mydata %>%

mutate(pop_global=ifelse(year==1990,pop1990,pop2021)) %>%

group_by(year,metric_name) %>%

arrange(sdi) %>%

mutate(cummu=cumsum(pop)) %>% # 计算累积人口

mutate(half=pop/2) %>% # 计算该国家人口的一半

mutate(midpoint=cummu-half) %>% # 累积人口减去该国家人口一半即为人口中点

mutate(weighted_order=midpoint/pop_global) # 人口中点与总人口相比即为改国的相对位置

# 把年份设置为 factor

rank$year <- factor(rank$year)

# 选择数据

temp1 <- rank %>%

filter(metric_name=="Rate") %>%

filter(year==1990)

temp2 <- rank %>%

filter(metric_name=="Rate") %>%

filter(year==2021)

# 建模计算斜度指数

fit1 <- lm(data = temp1,val~weighted_order)

fit2 <- lm(data = temp2,val~weighted_order)

coef(fit1)

coef(fit2)

# 查看是否存在异方差（存在异方差）

ncvTest(fit1)

ncvTest(fit2)

# 使用稳健（robust）回归：重复迭代加权

r.huber1 <- rlm(data = temp1,val~weighted_order)

r.huber2 <- rlm(data = temp2,val~weighted_order)

# 获得系数与截距

coef(r.huber1)

coef(r.huber2)

# 计算稳健回归的 95% 可信区间

confint.default(r.huber1) ####这里weighted_order的系数即为斜率指数

confint.default(r.huber2) ####这里weighted_order的系数即为斜率指数

library(ggpubr)

# 2.绘图 ----------------------------------------------------------------------

color <- c("#6699FF","#990000")

unique(rank$location_name)

colnames(rank)

rank <- rank %>%

mutate(location_name = case_when(

location_name == "United States of America" ~ "USA",

location_name == "Russian Federation" ~ "Russia",

location_name == "People's Republic of China" ~ "China",

TRUE ~ location_name # 保留其他国家的名称不变

))

#rank <- rank %>%

# mutate(

# cause_num = case_when(

# cause_name == "HIV/AIDS and sexually transmitted infections" ~ "1",

# cause_name == "Other infectious diseases" ~ "2",

# TRUE ~ "" # 其他病因留空（根据需求调整）

# ),

# 合并国家名称和病因编号标签（如 "USA 1"）

# label_text = paste(location_name, cause_num)

# )

p1 <- rank %>%

filter(metric_name=="Rate") %>%

ggplot(aes(x=weighted_order,y=val,fill=year,group=year,color=year))+

geom_point(aes(color=year,size=pop/1e6),alpha=0.8,shape=21)+

scale_size_area("Population\n(million)",breaks=c(50,100,150,200,250,300))+

geom_smooth(method = "rlm",size=0.6,alpha=0.1)+

scale_fill_manual(values = color)+

scale_color_manual(values = color)+

#增加水平虚线

geom_segment(x=0.02,xend=0.99,

y=0.6517272,yend=0.6517272, # coef(fit1)或coef(r.huber1)截距的位置

color="#6699FF",linetype=2,size=0.4,alpha=0.4)+

geom_segment(x=0.02,xend=0.99,

y=0.37925700,yend=0.37925700, # coef(r.huber2)截距的位置

color="#990000",linetype=2,size=0.4,alpha=0.4)+

# 增加某些国家的标签: 比如中国与印度

geom_text(aes(label=ifelse(location_name=="Russia"|location_name=="China"|location_name=="USA",as.character(location_name),""),

color=year),

hjust=0.8,vjust=2,# 避免点和文字重合

size=3)+

# # 增加斜度指数标签

annotate("text",label="Slope Index of Inequality",x=-0.06101473,y=-0.1631008,size=4,angle=90)+

annotate("text",label="-0.1631008",x=1.1,y=0.8,size=3.5)+ # coef(r.huber1) 权重的系数即斜率指数

annotate("text",label="-0.06101473",x=1.1,y=0.2,size=3.5)+ ##coef(r.huber2) 权重的系数即斜率指数

scale_x_continuous(limits = c(0,1.22),labels = c("0","0.25","0.50","0.75","1.00",""))+

xlab("Relative rank by SDI")+

ylab("Crude DALY rate (per 100,000)")+

theme_bw()

p1

# 集中指数的可视化 ----------------------------------------------------------------

# 1.绘图数据准备 ------------------------------------------------------------------

a <- mydata %>%

filter(metric_name=="Number") %>%

group_by(year) %>%

summarise(sum=sum(val))

daly1990 <- a$sum[1]

daly2021 <- a$sum[2]

ci <- rank %>%

filter(metric_name=="Number") %>%

mutate(total_daly=ifelse(year==1990,daly1990,daly2021)) %>%

group_by(year) %>%

arrange(sdi) %>%

mutate(cummu_daly=cumsum(val)) %>% # 计算累积 daly

mutate(frac_daly=cummu_daly/total_daly) %>% # 计算累积 daly 所占总体的比例

mutate(frac_population=cummu/pop_global) # 计算累积人口所占总体人口的比例

#####计算ci

# 选择数据

temp3 <- ci %>%

filter(metric_name=="Number") %>%

filter(year==1990)

temp4 <- ci %>%

filter(metric_name=="Number") %>%

filter(year==2021)

##计算集中指数

CI_1990 <- 2 * (sum(temp3$frac_daly) / nrow(temp3)) - 1

CI_2021 <- 2 * (sum(temp4$frac_daly) / nrow(temp4)) - 1

# 2.绘图 --------------------------------------------------------------------

#调整国家标签名称

ci <- ci %>%

mutate(location_name = case_when(

location_name == "United States of America" ~ "USA",

location_name == "Russian Federation" ~ "Russia",

location_name == "People's Republic of China" ~ "China",

TRUE ~ location_name # 保留其他国家的名称不变

))

p2 <- ci %>%

ggplot(aes(x=frac_population,y=frac_daly,fill=year,color=year,group=year))+

# 增加 X=0,y=0 两条线段

geom_segment(x=0,xend=1,

y=0,yend=0,

linetype=1,size=1,color="gray")+

geom_segment(x=1,xend=1,

y=0,yend=1,

linetype=1,size=1,color="gray")+

# 对角线

geom_segment(x=0,xend=1,

y=0,yend=1,

color="#CD853F",linetype=1,size=0.7,alpha=1)+

# 散点

geom_point(aes(fill=year,size=pop/1e6),alpha=0.75,shape=21)+

scale_fill_manual(values = color)+

scale_size_area("Population\n(million)",breaks=c(50,100,150,200,250,300))+

# 立方样条函数拟合洛伦兹曲线 (设置节点，边界条件)

geom_smooth(method = "gam", # 这里也可以直接用 geom_line 把点连起来

formula = y ~ ns(x,

knots = c(0.0000000001,0.25,0.5,0.75,0.9999999),# 设置节点为

Boundary.knots = c(0,1)),

linetype=1,size=0.1,alpha=0.6,se=T)+

scale_color_manual(values = color)+

# 增加两个年份的集中指数

annotate("text",label="Concentration Index",x=0.75,y=0.35,size=5)+

annotate("text",label="1990: 0.18",x=0.75,y=0.3,size=4,color="#6699FF")+

annotate("text",label="2021: 0.28",x=0.75,y=0.25,size=4,color="#990000")+

# 增加某些国家的标签，1990 年

# 步骤1：在数据中创建简化的病因编号列

geom_text(aes(label=ifelse(location_name=="USA"&year=="1990"|location_name=="Russia"&year=="1990"|location_name=="China"&year=="1990",

as.character(location_name),"")),

hjust=0.8,vjust=-1.4,

size=3)+

#增加某些国家的标签，2021 年

geom_text(aes(label=ifelse(location_name=="USA"&year=="2021"|location_name=="Russia"&year=="2021"|location_name=="China"&year=="2021",

as.character(location_name),"")),

hjust=0.8,vjust=-1.4,

size=3)+

#增加某些国家标签，人口

geom_text(aes(label=ifelse(location_name%in%a&year=="1990",

as.character(location_name),"")),

hjust=0.8,vjust=-1.4,

size=3)+

geom_text(aes(label=ifelse(location_name%in%a&year=="2021",

as.character(location_name),"")),

hjust=0.8,vjust=-1.4,

size=3)+

# xy 标签

xlab("Cumulative fraction of population ranked by SDI")+

ylab("Cumulative fraction of DALY")+

theme_bw()

p2

#########Deaths-------------------------------------------------------------

data1 <- read.csv("204countryDeaths.csv",header = T,check.names = F)

unique(data1$metric_name)

#########infections1###-----------------

data2 <- subset(data1,data1$measure_name=="Deaths"&

data1$sex_name=="Both"&

data1$age_name=="All ages"&

data1$cause_name=="HIV/AIDS and sexually transmitted infections"&

(data1$year==1990|data1$year==2021))

# sdi 数据

sdi <- read.csv("SDI_2021.csv",header = T,check.names = F)

sdi <- sdi %>% # 宽数据转为长数据

pivot_longer(cols = -Location,names_to = "year") %>%

rename(sdi = value)%>%

dplyr::select(Location,year,sdi)

sdi$year <- as.integer(sdi$year)

names(sdi)[1]<-"location_name"

# 匹配 sdi 与疾病负担, 生成 data

data3 <- left_join(data2,sdi,by=c("location_name","year"))

# 读取人口数据

path <- "GBD_population"

fileName <- list.files(path, pattern = "\\.csv$", full.names = FALSE) # 只匹配csv文件

# 初始化空数据框

population <- data.frame()

# 循环读取并合并

for (k in 1:length(fileName)) {

file_path <- file.path(path, fileName[k])

if (file.exists(file_path)) { # 检查文件是否存在

data <- read.csv(file_path, header = TRUE, stringsAsFactors = FALSE)

population <- rbind(population, data)

} else {

warning(paste("文件不存在:", file_path))

}

}

pop1 <- population %>%

dplyr::select(location_name,sex_name,age_name,year,metric_name,val)

unique(pop1$age_name)

pop1 <- pop1 %>%

filter(age_name=="All ages") %>%

dplyr::select("location_name","sex_name","year","val") %>%

rename(pop=val)

# 合并人口数据, 生成 mydata

mydata <- left_join(data3,pop1,

by=c("location_name","sex_name","year"))

# 斜度指数的可视化 ----------------------------------------------------------------

## 1.绘图数据的准备 -----------------------------------------------------------------

####### 计算总人口---------------

# 检查 pop 列的缺失值数量

sum(is.na(mydata$pop))

# 查看包含 NA 的行

mydata %>% filter(is.na(pop))

#用默认值填充缺失值（如0）

mydata <- mydata %>%

mutate(pop = ifelse(is.na(pop), 0, pop))

a <- mydata %>%

filter(metric_name=="Number") %>%

group_by(year) %>%

summarise(sum=sum(pop))

pop1990 <- a$sum[1]

pop2021 <- a$sum[2]

# 计算加权次序

rank <- mydata %>%

mutate(pop_global=ifelse(year==1990,pop1990,pop2021)) %>%

group_by(year,metric_name) %>%

arrange(sdi) %>%

mutate(cummu=cumsum(pop)) %>% # 计算累积人口

mutate(half=pop/2) %>% # 计算该国家人口的一半

mutate(midpoint=cummu-half) %>% # 累积人口减去该国家人口一半即为人口中点

mutate(weighted_order=midpoint/pop_global) # 人口中点与总人口相比即为改国的相对位置

# 把年份设置为 factor

rank$year <- factor(rank$year)

# 选择数据

temp1 <- rank %>%

filter(metric_name=="Rate") %>%

filter(year==1990)

temp2 <- rank %>%

filter(metric_name=="Rate") %>%

filter(year==2021)

# 建模计算斜度指数

fit1 <- lm(data = temp1,val~weighted_order)

fit2 <- lm(data = temp2,val~weighted_order)

coef(fit1)

coef(fit2)

# 查看是否存在异方差（存在异方差）

ncvTest(fit1)

ncvTest(fit2)

# 使用稳健（robust）回归：重复迭代加权

r.huber1 <- rlm(data = temp1,val~weighted_order)

r.huber2 <- rlm(data = temp2,val~weighted_order)

# 获得系数与截距

coef(r.huber1)

coef(r.huber2)

# 计算稳健回归的 95% 可信区间

confint.default(r.huber1) ####这里weighted_order的系数即为斜率指数

confint.default(r.huber2) ####这里weighted_order的系数即为斜率指数

library(ggpubr)

# 2.绘图 ----------------------------------------------------------------------

color <- c("#6699FF","#990000")

unique(rank$location_name)

colnames(rank)

rank <- rank %>%

mutate(location_name = case_when(

location_name == "United States of America" ~ "USA",

location_name == "Russian Federation" ~ "Russia",

location_name == "People's Republic of China" ~ "China",

TRUE ~ location_name # 保留其他国家的名称不变

))

#rank <- rank %>%

# mutate(

# cause_num = case_when(

# cause_name == "HIV/AIDS and sexually transmitted infections" ~ "1",

# cause_name == "Other infectious diseases" ~ "2",

# TRUE ~ "" # 其他病因留空（根据需求调整）

# ),

# 合并国家名称和病因编号标签（如 "USA 1"）

# label_text = paste(location_name, cause_num)

# )

p1 <- rank %>%

filter(metric_name=="Rate") %>%

ggplot(aes(x=weighted_order,y=val,fill=year,group=year,color=year))+

geom_point(aes(color=year,size=pop/1e6),alpha=0.8,shape=21)+

scale_size_area("Population\n(million)",breaks=c(50,100,150,200,250,300))+

geom_smooth(method = "rlm",size=0.6,alpha=0.1)+

scale_fill_manual(values = color)+

scale_color_manual(values = color)+

#增加水平虚线

geom_segment(x=0.02,xend=0.99,

y=0.14671181,yend=0.14671181, # coef(fit1)或coef(r.huber1)截距的位置

color="#6699FF",linetype=2,size=0.4,alpha=0.4)+

geom_segment(x=0.02,xend=0.99,

y=0.5595521,yend=0.5595521, # coef(r.huber2)截距的位置

color="#990000",linetype=2,size=0.4,alpha=0.4)+

# 增加某些国家的标签: 比如中国与印度

geom_text(aes(label=ifelse(location_name=="Russia"|location_name=="China"|location_name=="USA"|location_name=="India",as.character(location_name),""),

color=year),

hjust=0.8,vjust=2,# 避免点和文字重合

size=3)+

# # 增加斜度指数标签

annotate("text",label="Slope Index of Inequality",x= -0.2856374,y=0.02630262,size=4,angle=90)+

annotate("text",label="0.02630262",x=1.08,y=0.2,size=3.5)+ # coef(r.huber1) 权重的系数即斜率指数

annotate("text",label=" -0.2856374",x=1.08,y=0.4,size=3.5)+ ##coef(r.huber2) 权重的系数即斜率指数

scale_x_continuous(limits = c(0,1.22),labels = c("0","0.25","0.50","0.75","1.00",""))+

xlab("Relative rank by SDI")+

ylab("Crude DALY rate (per 100,000)")+

theme_bw()

p1

# 集中指数的可视化 ----------------------------------------------------------------

# 1.绘图数据准备 ------------------------------------------------------------------

a <- mydata %>%

filter(metric_name=="Number") %>%

group_by(year) %>%

summarise(sum=sum(val))

daly1990 <- a$sum[1]

daly2021 <- a$sum[2]

ci <- rank %>%

filter(metric_name=="Number") %>%

mutate(total_daly=ifelse(year==1990,daly1990,daly2021)) %>%

group_by(year) %>%

arrange(sdi) %>%

mutate(cummu_daly=cumsum(val)) %>% # 计算累积 daly

mutate(frac_daly=cummu_daly/total_daly) %>% # 计算累积 daly 所占总体的比例

mutate(frac_population=cummu/pop_global) # 计算累积人口所占总体人口的比例

#####计算ci

# 选择数据

temp3 <- ci %>%

filter(metric_name=="Number") %>%

filter(year==1990)

temp4 <- ci %>%

filter(metric_name=="Number") %>%

filter(year==2021)

##计算集中指数

CI_1990 <- 2 * (sum(temp3$frac_daly) / nrow(temp3)) - 1

CI_2021 <- 2 * (sum(temp4$frac_daly) / nrow(temp4)) - 1

# 2.绘图 --------------------------------------------------------------------

#调整国家标签名称

ci <- ci %>%

mutate(location_name = case_when(

location_name == "United States of America" ~ "USA",

location_name == "Russian Federation" ~ "Russia",

location_name == "People's Republic of China" ~ "China",

TRUE ~ location_name # 保留其他国家的名称不变

))

p2 <- ci %>%

ggplot(aes(x=frac_population,y=frac_daly,fill=year,color=year,group=year))+

# 增加 X=0,y=0 两条线段

geom_segment(x=0,xend=1,

y=0,yend=0,

linetype=1,size=1,color="gray")+

geom_segment(x=1,xend=1,

y=0,yend=1,

linetype=1,size=1,color="gray")+

# 对角线

geom_segment(x=0,xend=1,

y=0,yend=1,

color="#CD853F",linetype=1,size=0.7,alpha=1)+

# 散点

geom_point(aes(fill=year,size=pop/1e6),alpha=0.75,shape=21)+

scale_fill_manual(values = color)+

scale_size_area("Population\n(million)",breaks=c(50,100,150,200,250,300))+

# 立方样条函数拟合洛伦兹曲线 (设置节点，边界条件)

geom_smooth(method = "gam", # 这里也可以直接用 geom_line 把点连起来

formula = y ~ ns(x,

knots = c(0.0000000001,0.25,0.5,0.75,0.9999999),# 设置节点为

Boundary.knots = c(0,1)),

linetype=1,size=0.1,alpha=0.6,se=T)+

scale_color_manual(values = color)+

# 增加两个年份的集中指数

annotate("text",label="Concentration Index",x=0.75,y=0.35,size=5)+

annotate("text",label="1990: 0.16",x=0.75,y=0.3,size=4,color="#6699FF")+

annotate("text",label="2021: 0.08",x=0.75,y=0.25,size=4,color="#990000")+

# 增加某些国家的标签，1990 年

# 步骤1：在数据中创建简化的病因编号列

geom_text(aes(label=ifelse(location_name=="USA"&year=="1990"|location_name=="Russia"&year=="1990"|location_name=="China"&year=="1990"|location_name=="India"&year=="1990",

as.character(location_name),"")),

hjust=0.8,vjust=-1.4,

size=3)+

#增加某些国家的标签，2021 年

geom_text(aes(label=ifelse(location_name=="USA"&year=="2021"|location_name=="Russia"&year=="2021"|location_name=="China"&year=="2021"|location_name=="India"&year=="2021",

as.character(location_name),"")),

hjust=0.8,vjust=-1.4,

size=3)+

#增加某些国家标签，人口

geom_text(aes(label=ifelse(location_name%in%a&year=="1990",

as.character(location_name),"")),

hjust=0.8,vjust=-1.4,

size=3)+

geom_text(aes(label=ifelse(location_name%in%a&year=="2021",

as.character(location_name),"")),

hjust=0.8,vjust=-1.4,

size=3)+

# xy 标签

xlab("Cumulative fraction of population ranked by SDI")+

ylab("Cumulative fraction of DALY")+

theme_bw()

p2

#########infections2###-----------------

data2 <- subset(data1,data1$measure_name=="Deaths"&

data1$sex_name=="Both"&

data1$age_name=="All ages"&

data1$cause_name=="Other infectious diseases"&

(data1$year==1990|data1$year==2021))

# sdi 数据

sdi <- read.csv("SDI_2021.csv",header = T,check.names = F)

sdi <- sdi %>% # 宽数据转为长数据

pivot_longer(cols = -Location,names_to = "year") %>%

rename(sdi = value)%>%

dplyr::select(Location,year,sdi)

sdi$year <- as.integer(sdi$year)

names(sdi)[1]<-"location_name"

# 匹配 sdi 与疾病负担, 生成 data

data3 <- left_join(data2,sdi,by=c("location_name","year"))

# 读取人口数据

path <- "GBD_population"

fileName <- list.files(path, pattern = "\\.csv$", full.names = FALSE) # 只匹配csv文件

# 初始化空数据框

population <- data.frame()

# 循环读取并合并

for (k in 1:length(fileName)) {

file_path <- file.path(path, fileName[k])

if (file.exists(file_path)) { # 检查文件是否存在

data <- read.csv(file_path, header = TRUE, stringsAsFactors = FALSE)

population <- rbind(population, data)

} else {

warning(paste("文件不存在:", file_path))

}

}

pop1 <- population %>%

dplyr::select(location_name,sex_name,age_name,year,metric_name,val)

unique(pop1$age_name)

pop1 <- pop1 %>%

filter(age_name=="All ages") %>%

dplyr::select("location_name","sex_name","year","val") %>%

rename(pop=val)

# 合并人口数据, 生成 mydata

mydata <- left_join(data3,pop1,

by=c("location_name","sex_name","year"))

# 斜度指数的可视化 ----------------------------------------------------------------

## 1.绘图数据的准备 -----------------------------------------------------------------

####### 计算总人口---------------

# 检查 pop 列的缺失值数量

sum(is.na(mydata$pop))

# 查看包含 NA 的行

mydata %>% filter(is.na(pop))

#用默认值填充缺失值（如0）

mydata <- mydata %>%

mutate(pop = ifelse(is.na(pop), 0, pop))

a <- mydata %>%

filter(metric_name=="Number") %>%

group_by(year) %>%

summarise(sum=sum(pop))

pop1990 <- a$sum[1]

pop2021 <- a$sum[2]

# 计算加权次序

rank <- mydata %>%

mutate(pop_global=ifelse(year==1990,pop1990,pop2021)) %>%

group_by(year,metric_name) %>%

arrange(sdi) %>%

mutate(cummu=cumsum(pop)) %>% # 计算累积人口

mutate(half=pop/2) %>% # 计算该国家人口的一半

mutate(midpoint=cummu-half) %>% # 累积人口减去该国家人口一半即为人口中点

mutate(weighted_order=midpoint/pop_global) # 人口中点与总人口相比即为改国的相对位置

# 把年份设置为 factor

rank$year <- factor(rank$year)

# 选择数据

temp1 <- rank %>%

filter(metric_name=="Rate") %>%

filter(year==1990)

temp2 <- rank %>%

filter(metric_name=="Rate") %>%

filter(year==2021)

# 建模计算斜度指数

fit1 <- lm(data = temp1,val~weighted_order)

fit2 <- lm(data = temp2,val~weighted_order)

coef(fit1)

coef(fit2)

# 查看是否存在异方差（存在异方差）

ncvTest(fit1)

ncvTest(fit2)

# 使用稳健（robust）回归：重复迭代加权

r.huber1 <- rlm(data = temp1,val~weighted_order)

r.huber2 <- rlm(data = temp2,val~weighted_order)

# 获得系数与截距

coef(r.huber1)

coef(r.huber2)

# 计算稳健回归的 95% 可信区间

confint.default(r.huber1) ####这里weighted_order的系数即为斜率指数

confint.default(r.huber2) ####这里weighted_order的系数即为斜率指数

library(ggpubr)

# 2.绘图 ----------------------------------------------------------------------

color <- c("#6699FF","#990000")

unique(rank$location_name)

colnames(rank)

rank <- rank %>%

mutate(location_name = case_when(

location_name == "United States of America" ~ "USA",

location_name == "Russian Federation" ~ "Russia",

location_name == "People's Republic of China" ~ "China",

TRUE ~ location_name # 保留其他国家的名称不变

))

#rank <- rank %>%

# mutate(

# cause_num = case_when(

# cause_name == "HIV/AIDS and sexually transmitted infections" ~ "1",

# cause_name == "Other infectious diseases" ~ "2",

# TRUE ~ "" # 其他病因留空（根据需求调整）

# ),

# 合并国家名称和病因编号标签（如 "USA 1"）

# label_text = paste(location_name, cause_num)

# )

p1 <- rank %>%

filter(metric_name=="Rate") %>%

ggplot(aes(x=weighted_order,y=val,fill=year,group=year,color=year))+

geom_point(aes(color=year,size=pop/1e6),alpha=0.8,shape=21)+

scale_size_area("Population\n(million)",breaks=c(50,100,150,200,250,300))+

geom_smooth(method = "rlm",size=0.6,alpha=0.1)+

scale_fill_manual(values = color)+

scale_color_manual(values = color)+

#增加水平虚线

geom_segment(x=0.02,xend=0.99,

y=0.016205302,yend=0.016205302, # coef(fit1)或coef(r.huber1)截距的位置

color="#6699FF",linetype=2,size=0.4,alpha=0.4)+

geom_segment(x=0.02,xend=0.99,

y=0.010615743,yend=0.010615743, # coef(r.huber2)截距的位置

color="#990000",linetype=2,size=0.4,alpha=0.4)+

# 增加某些国家的标签: 比如中国与印度

geom_text(aes(label=ifelse(location_name=="Russia"|location_name=="China"|location_name=="USA"|location_name=="India",as.character(location_name),""),

color=year),

hjust=0.8,vjust=2,# 避免点和文字重合

size=3)+

# # 增加斜度指数标签

annotate("text",label="Slope Index of Inequality",x=-0.007340131,y=-0.009046735,size=4,angle=90)+

annotate("text",label="-0.009046735",x=1.1,y=0.02,size=3.5)+ # coef(r.huber1) 权重的系数即斜率指数

annotate("text",label="-0.007340131",x=1.1,y=0.001,size=3.5)+ ##coef(r.huber2) 权重的系数即斜率指数

scale_x_continuous(limits = c(0,1.22),labels = c("0","0.25","0.50","0.75","1.00",""))+

xlab("Relative rank by SDI")+

ylab("Crude Deaths rate (per 100,000)")+

theme_bw()

p1

# 集中指数的可视化 ----------------------------------------------------------------

# 1.绘图数据准备 ------------------------------------------------------------------

a <- mydata %>%

filter(metric_name=="Number") %>%

group_by(year) %>%

summarise(sum=sum(val))

daly1990 <- a$sum[1]

daly2021 <- a$sum[2]

ci <- rank %>%

filter(metric_name=="Number") %>%

mutate(total_daly=ifelse(year==1990,daly1990,daly2021)) %>%

group_by(year) %>%

arrange(sdi) %>%

mutate(cummu_daly=cumsum(val)) %>% # 计算累积 daly

mutate(frac_daly=cummu_daly/total_daly) %>% # 计算累积 daly 所占总体的比例

mutate(frac_population=cummu/pop_global) # 计算累积人口所占总体人口的比例

#####计算ci

# 选择数据

temp3 <- ci %>%

filter(metric_name=="Number") %>%

filter(year==1990)

temp4 <- ci %>%

filter(metric_name=="Number") %>%

filter(year==2021)

##计算集中指数

CI_1990 <- 2 * (sum(temp3$frac_daly) / nrow(temp3)) - 1

CI_2021 <- 2 * (sum(temp4$frac_daly) / nrow(temp4)) - 1

# 2.绘图 --------------------------------------------------------------------

#调整国家标签名称

ci <- ci %>%

mutate(location_name = case_when(

location_name == "United States of America" ~ "USA",

location_name == "Russian Federation" ~ "Russia",

location_name == "People's Republic of China" ~ "China",

TRUE ~ location_name # 保留其他国家的名称不变

))

p2 <- ci %>%

ggplot(aes(x=frac_population,y=frac_daly,fill=year,color=year,group=year))+

# 增加 X=0,y=0 两条线段

geom_segment(x=0,xend=1,

y=0,yend=0,

linetype=1,size=1,color="gray")+

geom_segment(x=1,xend=1,

y=0,yend=1,

linetype=1,size=1,color="gray")+

# 对角线

geom_segment(x=0,xend=1,

y=0,yend=1,

color="#CD853F",linetype=1,size=0.7,alpha=1)+

# 散点

geom_point(aes(fill=year,size=pop/1e6),alpha=0.75,shape=21)+

scale_fill_manual(values = color)+

scale_size_area("Population\n(million)",breaks=c(50,100,150,200,250,300))+

# 立方样条函数拟合洛伦兹曲线 (设置节点，边界条件)

geom_smooth(method = "gam", # 这里也可以直接用 geom_line 把点连起来

formula = y ~ ns(x,

knots = c(0.0000000001,0.25,0.5,0.75,0.9999999),# 设置节点为

Boundary.knots = c(0,1)),

linetype=1,size=0.1,alpha=0.6,se=T)+

scale_color_manual(values = color)+

# 增加两个年份的集中指数

annotate("text",label="Concentration Index",x=0.75,y=0.35,size=5)+

annotate("text",label="1990: 0.32",x=0.75,y=0.3,size=4,color="#6699FF")+

annotate("text",label="2021: 0.41",x=0.75,y=0.25,size=4,color="#990000")+

# 增加某些国家的标签，1990 年

# 步骤1：在数据中创建简化的病因编号列

geom_text(aes(label=ifelse(location_name=="USA"&year=="1990"|location_name=="Russia"&year=="1990"|location_name=="China"&year=="1990"|location_name=="India"&year=="1990",

as.character(location_name),"")),

hjust=0.8,vjust=-1.4,

size=3)+

#增加某些国家的标签，2021 年

geom_text(aes(label=ifelse(location_name=="USA"&year=="2021"|location_name=="Russia"&year=="2021"|location_name=="China"&year=="2021"|location_name=="India"&year=="2021",

as.character(location_name),"")),

hjust=0.8,vjust=-1.4,

size=3)+

#增加某些国家标签，人口

geom_text(aes(label=ifelse(location_name%in%a&year=="1990",

as.character(location_name),"")),

hjust=0.8,vjust=-1.4,

size=3)+

geom_text(aes(label=ifelse(location_name%in%a&year=="2021",

as.character(location_name),"")),

hjust=0.8,vjust=-1.4,

size=3)+

# xy 标签

xlab("Cumulative fraction of population ranked by SDI")+

ylab("Cumulative fraction of DALY")+

theme_bw()

p2

######斜率指数的年份图：

####利用上述方法计算出各个年份的SII数据

# 示例数据

sii_data <- data.frame(

year = 1990:2019,

SII = c(-120, -110, -110, -102, -100, -95, -90, -85, -80, -75,

-70, -65, -20, -50, -50, -40, -40, -35, -30, -25,

-20, -15, -10, -5, 0, 5, 10, 25, 40, 47) #####这里的SII数值是瞎编的可以行按之前的方法计算

)

# 绘制SII趋势图

ggplot(sii_data, aes(x = year, y = SII)) +

geom_point() + # 绘制数据点

geom_smooth(method = "lm", se = TRUE) + # 添加回归线和置信区间

labs(x = "Year", y = "SII") + # 设置轴标签

theme_minimal() # 使用简洁主题

**PART 16 Relationship Between SDI and ASMR/ASDR of Infectious Diseases Attributable to Drug Use Risk Factors by Global and 21 GBD Regions from 1990 to 2021.**

setwd("D:/ SDI 22地区")

library(reshape)

library(ggplot2)

library(ggrepel)

library(readxl)

IBD <- read.csv('IBD_region.csv',header = T) # 读取21个地区的数据

unique(IBD_22$cause_name)

IBD_super <-read.csv('IBD_super_region.csv',header = T) ##读取超级地区及全球数据

IBD_Global<-subset(IBD_super,IBD_super$location_name=="Global")###提取全球数据

IBD_22 <- rbind(IBD,IBD_Global) ###把全球数据与21个地区的数据合并

order_SDI <- read.csv('order_SDI.csv',header = F)

SDI_2021<-read.csv("SDI_2021.csv",header = T)

SDI_2021<-SDI_2021[,-1]

## 用到reshape包，将SDI数据格式从宽数据格式转换为长数据格式

SDI_2021 <- melt(SDI_2021,id.vars ='Location')

SDI_2021$variable <- as.numeric(gsub('\\X',replacement = '', SDI_2021$variable))

names(SDI_2021) <- c('location','year','SDI')

#改变量名，SDI与GBD保持一致

SDI_2021$location[which(SDI_2021$location =='Central sub-Saharan Africa')] <-'Central Sub-Saharan Africa'

SDI_2021$location[which(SDI_2021$location =='Eastern sub-Saharan Africa')] <-'Eastern Sub-Saharan Africa'

SDI_2021$location[which(SDI_2021$location =='Southern sub-Saharan Africa')] <-'Southern Sub-Saharan Africa'

SDI_2021$location[which(SDI_2021$location =='Western sub-Saharan Africa')] <-'Western Sub-Saharan Africa'

### ASMR1#####

ASMR1 <- subset(IBD_22, IBD_22$age_name=='Age-standardized' &

IBD_22$metric_name== 'Rate' &

IBD_22$measure_name=='Deaths'&

IBD_22$cause_name=='HIV/AIDS and sexually transmitted infections'&

IBD_22$sex_name=="Both")

ASMR1 <- ASMR1[,c(5,16,17)] ###选择location year val

names(ASMR1)[3] <- 'ASMR'

names(ASMR1)[1] <- 'location'

### 合并SDI与ASR数据

ASMR1_SDI <- merge(ASMR1,SDI_2021,by=c('location','year'))

ASMR1_SDI$location <- factor(ASMR1_SDI$location,

levels=order_SDI$V1,

ordered=TRUE) ## location图例按照我们的顺序排列

write.csv(ASMR1_SDI,"ASMR1_SDI.csv")

##开始作图，主变量为ASR以及SDI,图形的颜色和形状根据不同区域来调整即可

#同时以所有数据画出拟合曲线

size_breaks <- seq(min(ASMR1_SDI$year), max(ASMR1_SDI$year), by = 5)

size_labels <- size_breaks

ggplot(ASMR1_SDI, aes(SDI,ASMR)) + geom_point(aes(color = location, shape= location,size=year))+

scale_shape_manual(values = 1:22) +

labs(x = "Socio-Demographic Index",

y = "ASMR(per 100000)") +

geom_smooth(colour='black',stat = "smooth",method='loess',se=F,span=0.5)+

scale_size_continuous(breaks = size_breaks, labels = size_labels,range = c(1, 3))

result <- cor.test(ASMR1_SDI$ASMR,ASMR1_SDI$SDI , method = "spearman")

summary(result)

######Global+21地区ASMR-SDI.2------------------------------------------

### ASMR2#####

unique(IBD_22$cause_name)

ASMR2 <- subset(IBD_22, IBD_22$age_name=='Age-standardized' &

IBD_22$metric_name== 'Rate' &

IBD_22$measure_name=='Deaths'&

IBD_22$cause_name=='Other infectious diseases'&

IBD_22$sex_name=="Both")

ASMR2 <- ASMR2[,c(5,16,17)] ###选择location year val

names(ASMR2)[3] <- 'ASMR'

names(ASMR2)[1] <- 'location'

### 合并SDI与ASR数据

ASMR2_SDI <- merge(ASMR2,SDI_2021,by=c('location','year'))

ASMR2_SDI$location <- factor(ASMR2_SDI$location,

levels=order_SDI$V1,

ordered=TRUE) ## location图例按照我们的顺序排列

write.csv(ASMR2_SDI,"ASMR2_SDI.csv")

##开始作图，主变量为ASR以及SDI,图形的颜色和形状根据不同区域来调整即可

#同时以所有数据画出拟合曲线

size_breaks <- seq(min(ASMR2_SDI$year), max(ASMR2_SDI$year), by = 5)

size_labels <- size_breaks

ggplot(ASMR2_SDI, aes(SDI,ASMR)) + geom_point(aes(color = location, shape= location,size=year))+

scale_shape_manual(values = 1:22) +

labs(x = "Socio-Demographic Index",

y = "ASMR(per 100000)" )+

geom_smooth(colour='black',stat = "smooth",method='loess',se=F,span=0.5)+

scale_size_continuous(breaks = size_breaks, labels = size_labels,range = c(1, 3))

result <- cor.test(ASMR2_SDI$ASMR,ASMR2_SDI$SDI , method = "spearman")

summary(result)

#######ASDR 1------------------------------------------------------------

ASDR1 <- subset(IBD_22, IBD_22$age_name=='Age-standardized' &

IBD_22$metric_name== 'Rate' &

IBD_22$measure_name=='DALYs (Disability-Adjusted Life Years)'&

IBD_22$cause_name=='HIV/AIDS and sexually transmitted infections'&

IBD_22$sex_name=="Both")

ASDR1 <- ASDR1[,c(5,16,17)] ###选择location year val

names(ASDR1)[3] <- 'ASDR'

names(ASDR1)[1] <- 'location'

### 合并SDI与ASR数据

ASDR1_SDI <- merge(ASDR1,SDI_2021,by=c('location','year'))

ASDR1_SDI$location <- factor(ASDR1_SDI$location,

levels=order_SDI$V1,

ordered=TRUE) ## location图例按照我们的顺序排列

write.csv(ASDR1_SDI,"ASDR1_SDI.csv")

##开始作图，主变量为ASR以及SDI,图形的颜色和形状根据不同区域来调整即可

#同时以所有数据画出拟合曲线

size_breaks <- seq(min(ASDR1_SDI$year), max(ASDR1_SDI$year), by = 5)

size_labels <- size_breaks

ggplot(ASDR1_SDI, aes(SDI,ASDR)) + geom_point(aes(color = location, shape= location,size=year))+

scale_shape_manual(values = 1:22) +

labs(x = "Socio-Demographic Index",

y = "ASDR(per 100000)") +

geom_smooth(colour='black',stat = "smooth",method='loess',se=F,span=0.5)+

scale_size_continuous(breaks = size_breaks, labels = size_labels,range = c(1, 3))

result <- cor.test(ASDR1_SDI$ASDR,ASDR1_SDI$SDI , method = "spearman")

summary(result)

#######ASDR 2------------------------------------------------------------

ASDR2 <- subset(IBD_22, IBD_22$age_name=='Age-standardized' &

IBD_22$metric_name== 'Rate' &

IBD_22$measure_name=='DALYs (Disability-Adjusted Life Years)'&

IBD_22$cause_name=='Other infectious diseases'&

IBD_22$sex_name=="Both")

ASDR2 <- ASDR2[,c(5,16,17)] ###选择location year val

names(ASDR2)[3] <- 'ASDR'

names(ASDR2)[1] <- 'location'

### 合并SDI与ASR数据

ASDR2_SDI <- merge(ASDR2,SDI_2021,by=c('location','year'))

ASDR2_SDI$location <- factor(ASDR2_SDI$location,

levels=order_SDI$V1,

ordered=TRUE) ## location图例按照我们的顺序排列

write.csv(ASDR2_SDI,"ASDR2_SDI.csv")

##开始作图，主变量为ASR以及SDI,图形的颜色和形状根据不同区域来调整即可

#同时以所有数据画出拟合曲线

size_breaks <- seq(min(ASDR2_SDI$year), max(ASDR2_SDI$year), by = 5)

size_labels <- size_breaks

ggplot(ASDR2_SDI, aes(SDI,ASDR)) + geom_point(aes(color = location, shape= location,size=year))+

scale_shape_manual(values = 1:22) +

labs(x = "Socio-Demographic Index",

y = "ASDR(per 100000)") +

geom_smooth(colour='black',stat = "smooth",method='loess',se=F,span=0.5)+

scale_size_continuous(breaks = size_breaks, labels = size_labels,range = c(1, 3))

result <- cor.test(ASDR2_SDI$ASDR,ASDR2_SDI$SDI , method = "spearman")

summary(result)
