# Supplementary material for: Global burden and forecast of infectious diseases attributable to drug use: evidence from GBD 2021
Source: Front Public Health. 2025 Dec 15;13:1706764. doi: 10.3389/fpubh.2025.1706764 (PMC12745377; doi:10.3389/fpubh.2025.1706764)
Supplement: Supplementary file 3 [file Data_Sheet_3.DOCX]

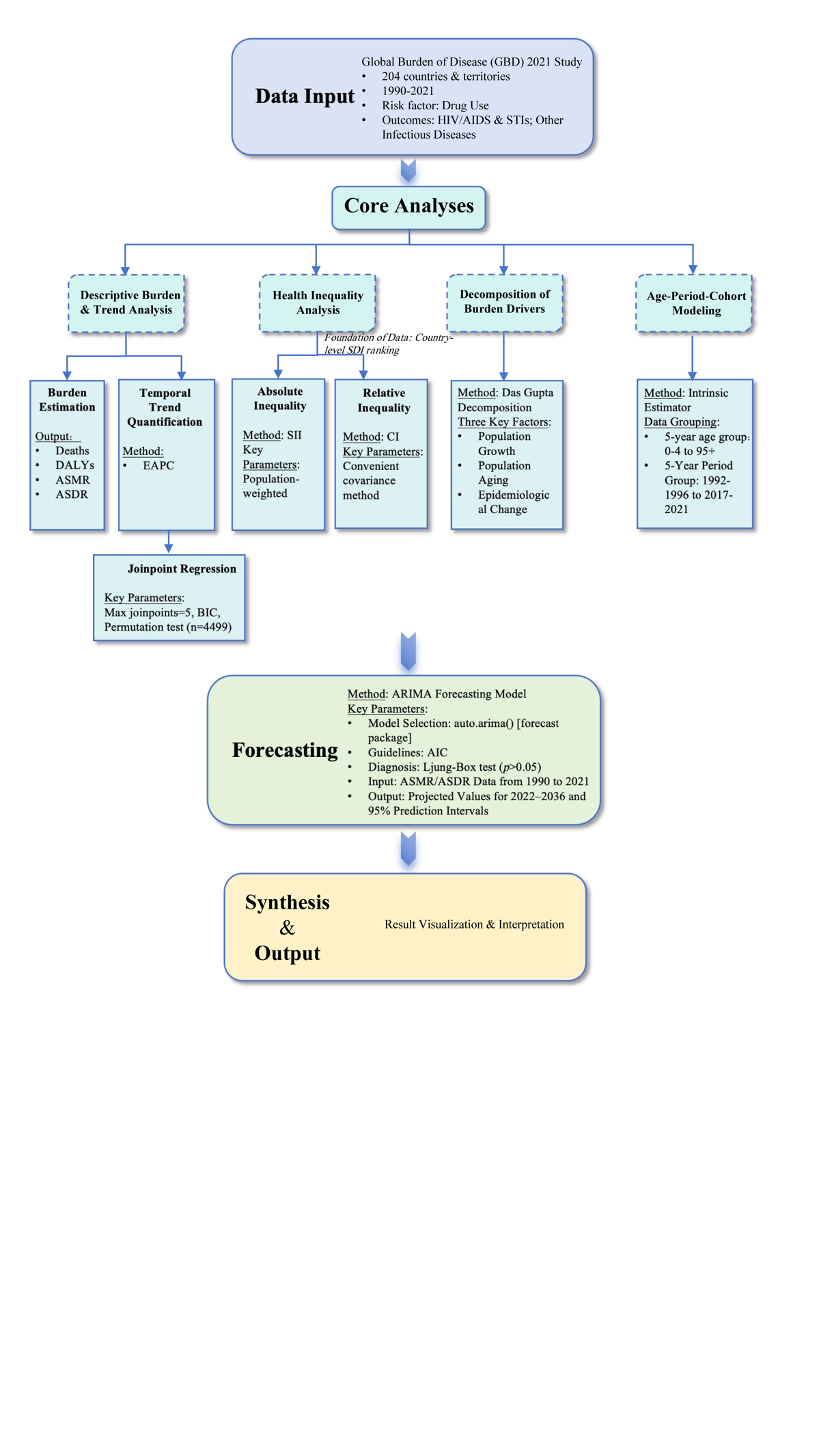


**Supplementary Figure 1.** Analytical Framework for Assessing the Global Burden of Infectious Diseases Attributable to Drug Use. DALYs: Disability-Adjusted Life Years. ASMR: Age-standardized Mortality Rate. ASDR: Age-standardized Disability-adjusted Life Years Rate. EAPC: Estimated Annual Percentage Change. SII: Slope Inequality Index. CI: Concentration Index. BIC: Bayesian Information Criterion. AIC: Akaike Information Criterion**.**

**Supplementary Table 1. The APC and AAPC by Joinpoint Regression Analysis of the Burden of Infectious Diseases Attributable to Drug Use Risk Factors, 1990-2021**

| **Gender** | **Age-standardized DALYs** | | |  | **Age-standardized Deaths** | | |
| --- | --- | --- | --- | --- | --- | --- | --- |
|  | **Period** | **APC (95% CI)** | **AAPC (95% CI)** |  | **Period** | **APC (95% CI)** | **AAPC (95% CI)** |
| **HIV/AIDS and sexually transmitted infections** | | | | | | | |
| **Both** | 1990-1994 | 21.961 *  (20.9703~22.9598) | 2.7091 *  (2.4558~2.9629) |  | 1990-1994 | 22.3792 *  (21.3879~23.3786) | 2.8212 *  (2.5739~3.069) |
|  | 1994-2000 | 7.6637 *  (6.9998~8.3317) |  |  | 1994-2000 | 8.16 *  (7.5001~8.8239) |  |
|  | 2000-2005 | 2.5935 *  (1.7223~3.4721) |  |  | 2000-2005 | 2.7313 *  (1.8711~3.5987) |  |
|  | 2005-2013 | -3.9166 *  (-4.1985~-3.6338) |  |  | 2005-2013 | -4.1909 *  (-4.4685~-3.9125) |  |
|  | 2013-2018 | -1.6606 *  (-2.2905~-1.0266) |  |  | 2013-2018 | -1.3347 *  (-1.9382~-0.7276) |  |
|  | 2018-2021 | -4.3433 *  (-5.387~-3.2881) |  |  | 2018-2021 | -4.5931 *  (-5.5596~-3.6167) |  |
| **Male** | 1990-1994 | 19.9381 *  (18.9216~20.9633) | 2.379 *  (2.121 ~2.6376) |  | 1990-1994 | 20.4242 *  (19.3109~21.548) | 2.5611 *  (2.273~2.8501) |
|  | 1994-2000 | 6.0666 *  (5.3968~6.7408) |  |  | 1994-2000 | 6.4978 *  (5.7636~7.237) |  |
|  | 2000-2006 | 1.4367 *  (0.8068~2.0705) |  |  | 2000-2005 | 2.322 *  (1.3264~3.3273) |  |
|  | 2006-2013 | -3.3234 *  (-3.6988~-2.9465) |  |  | 2005-2013 | -3.2529 *  (-3.5756~-2.9292) |  |
|  | 2013-2017 | -0.7595  (-1.7676~0.2589) |  |  | 2013-2017 | -0.3743  (-1.45~0.7132) |  |
|  | 2017-2021 | -4.1673 *  (-4.8517~-3.478) |  |  | 2017-2021 | -4.2179 *  (-4.9354~-3.495) |  |
| **Female** | 1990-1994 | 28.7306 *  (26.5942~30.9031) | 3.9268 *  (3.3734~4.4831) |  | 1990-1994 | 29.2704 *  (26.8441~31.7432) | 3.9231 *  (3.3142~4.5356) |
|  | 1994-1999 | 12.5378 *  (10.6592~14.4483) |  |  | 1994-1999 | 12.8317 *  (10.7443~14.9584) |  |
|  | 1999-2003 | 6.307 *  (3.747 ~8.9303) |  |  | 1999-2003 | 6.5682 *  (3.7644~9.4477) |  |
|  | 2003-2007 | -1.7071  (-3.8112~0.4431) |  |  | 2003-2007 | -1.9411  (-4.2279~0.4004) |  |
|  | 2007-2014 | -6.1038 *  (-6.7108~-5.4927) |  |  | 2007-2014 | -6.4865 *  (-7.1436~-5.8248) |  |
|  | 2014-2021 | -2.004 *  (-2.591~-1.4136) |  |  | 2014-2021 | -2.0399 *  (-2.6733~-1.4024) |  |
| **Other infectious diseases** | | | | | | | |
| **Both** | 1990-1994 | -1.9072 *  (-2.5477~-1.2624) | -2.3479 *  (-2.5763~2.1189) |  | 1990-1994 | -2.0578 *  (-2.5641~-1.5488) | -2.7065 *  (-2.8356~-2.5773) |
|  | 1994-2001 | -3.5246 *  (-3.8396~-3.2085) |  |  | 1994-2001 | -3.9781 *  (-4.2244~-3.7313) |  |
|  | 2001-2005 | -4.4325 *  (-5.3159~-3.5409) |  |  | 2001-2005 | -5.7291 *  (-6.4081~-5.0451) |  |
|  | 2005-2012 | -1.3282 *  (-1.6418~-1.0137) |  |  | 2005-2015 | -1.7927 *  (-1.9231~-1.6622) |  |
|  | 2012-2015 | -2.2913 *  (-4.0139~-0.5377) |  |  | 2015-2021 | -1.1000 *  (-1.3213~-0.8781) |  |
|  | 2015-2021 | -1.0584 *  (-1.3435~-0.7724) |  |  |  |  |  |
| **Male** | 1990-1994 | -3.5290 *  (-4.1311~-2.9231) | -3.3472 *  (-3.5127~-3.18141) |  | 1990-1994 | -3.6293 *  (-4.4756~-2.7756) | -3.7738 *  (-3.9725~-3.5746) |
|  | 1994-2001 | -5.7646 *  (-6.0425~-5.4859) |  |  | 1994-2001 | -6.1106 *  (-6.5021~-5.7174) |  |
|  | 2001-2005 | -7.2805 *  (-7.9790~-6.5766) |  |  | 2001-2005 | -8.3528 *  (-9.3327~-7.3624) |  |
|  | 2005-2010 | -1.3255 *  (-1.8143~-0.8342) |  |  | 2005-2015 | -2.2433 *  (-2.4281~-2.0582) |  |
|  | 2010-2015 | -2.1556 *  (-2.6096~-1.6994) |  |  | 2015-2021 | -0.4571 *  (-0.7927~-0.1203) |  |
|  | 2015-2021 | -0.2999 *  (-0.5411~-0.0582) |  |  |  |  |  |
| **Female** | 1990-1995 | 2.4098 *  (1.7580~3.0659) | -0.4978 *  (-0.6744~-0.32091) |  | 1990-1995 | 2.1704 *  (1.6504~2.6932) | -0.7253 *  (-0.9113~-0.5391) |
|  | 1995-2002 | 0.4161  (-0.0702~0.9048) |  |  | 1995-2001 | -0.0426  (-0.5355~0.4528) |  |
|  | 2002-2012 | -1.1720 *  (-1.4353~-0.9080) |  |  | 2001-2008 | -1.9767 *  (-2.3527~-1.5993) |  |
|  | 2012-2021 | -2.0339 *  (-2.2839~-1.7832) |  |  | 2008-2013 | -0.9288 *  (-1.6274~-0.2253) |  |
|  |  |  |  |  | 2013-2021 | -1.7847 *  (-2.0218~-1.5471) |  |

* means *P*<0.05

**Supplementary Table 2. Global Trends in HIV/AIDS and Sexually Transmitted Infections: Changes in DALYs and Deaths by Population-Level Determinants and Etiological Causes, 1990–2021**

|  | **Overll difference** | **Change due to Population-level determinants**  **(% contribute to the total changes)** | | |
| --- | --- | --- | --- | --- |
|  |  | **Aging** | **Population** | **Epidemiological change** |
| **DALYs** |  |  |  |  |
| Both | 2621088.04 | 150262.918(5.73%) | 820905.576(31.32%) | 1649919.548(62.95%) |
| Male | 1790682.1 | 119664.698(6.68%) | 599596.331(33.48%) | 1071421.068(59.83%) |
| Female | 838988.97 | 31230.467(3.72%) | 218818.226(26.08%) | 588940.275(70.2%) |
| **Deaths** |  |  |  |  |
| Both | 48243.82 | 5938.815(12.31%) | 15011.611(31.12%) | 27293.399(56.57%) |
| Male | 33319.43 | 4432.619(13.3%) | 11065.39(33.21%) | 17821.418(53.49%) |
| Female | 14935.97 | 1488.919(9.97%) | 3883.59(26%) | 9563.46(64.03%) |

**Supplementary Table 3.** **Global Trends in Other Infectious Diseases: Changes in DALYs and Deaths by Population-Level Determinants and Etiological Causes, 1990–2021**

|  | **Overll difference** | **Change due to Population-level determinants**  **(% contribute to the total changes)** | | |
| --- | --- | --- | --- | --- |
|  |  | **Aging** | **Population** | **Epidemiological change** |
| **DALYs** |  |  |  |  |
| Both | -7405.32 | 13678.687(12.54%) | 37176.854(34.07%) | -58260.859(-53.39%) |
| Male | -21019.96 | 8535.281(9.56%) | 25594.771(28.67%) | -55150.013(-61.77%) |
| Female | 13074.6 | 4483.757(22.12%) | 12190.687(60.13%) | -3599.848(-17.76%) |
| **Deaths** |  |  |  |  |
| Both | -1267.03 | 281.073(5.96%) | 1444.454(30.62%) | -2992.554(-63.43%) |
| Male | -1554.25 | 231.275(5.74%) | 1005.797(24.97%) | -2791.325(-69.29%) |
| Female | 251.75 | 70.398(8.86%) | 452.949(56.98%) | -271.601(-34.17%) |

**Supplementary Table 4. Age-Period-Cohort Effects of Infectious Diseases Attributable to Drug Use Risk** **Mortality Rate in Global**

| **Factors** | **Mortality Rate** | | |
| --- | --- | --- | --- |
|  | **RR** | **95% CI** | |
|  |  | **Lower** | **Upper** |
| **Age** |  |  |  |
| 0-4 | 0 | 0 | 0.077 |
| 5-9 | 0 | 0 | 0.035 |
| 10-14 | 0 | 0 | 0.022 |
| 15-19 | 0.029 | 0.024 | 0.036 |
| 20-24 | 0.185 | 0.17 | 0.202 |
| 25-29 | 0.979 | 0.935 | 1.024 |
| 30-34 | 2.217 | 2.139 | 2.298 |
| 35-39 | 3.194 | 3.098 | 3.293 |
| 40-44 | 3.554 | 3.455 | 3.656 |
| 45-49 | 3.002 | 2.917 | 3.091 |
| 50-54 | 2.091 | 2.02 | 2.164 |
| 55-59 | 1.423 | 1.363 | 1.487 |
| 60-64 | 1.035 | 0.978 | 1.095 |
| 65-69 | 0.905 | 0.842 | 0.973 |
| 70-74 | 0.64 | 0.579 | 0.708 |
| 75-79 | 0.543 | 0.476 | 0.619 |
| 80-84 | 0.599 | 0.507 | 0.706 |
| 85-89 | 0.528 | 0.41 | 0.678 |
| 90-94 | 0.501 | 0.324 | 0.775 |
| 95-100 | 0.461 | 0.188 | 1.128 |
| **Period** |  |  |  |
| 1994.5 | 0.576 | 0.443 | 0.75 |
| 1999.5 | 0.843 | 0.738 | 0.964 |
| 2004.5 | 1 | 1 | 1 |
| 2009.5 | 0.91 | 0.796 | 1.04 |
| 2014.5 | 0.786 | 0.604 | 1.023 |
| 2019.5 | 0.752 | 0.507 | 1.115 |
| **Cohort** |  |  |  |
| 1897 | 0.226 | 0.001 | 48.058 |
| 1902 | 0.276 | 0.042 | 1.829 |
| 1907 | 0.34 | 0.146 | 0.793 |
| 1912 | 0.45 | 0.29 | 0.698 |
| 1917 | 0.631 | 0.482 | 0.827 |
| 1922 | 0.673 | 0.557 | 0.812 |
| 1927 | 0.713 | 0.623 | 0.816 |
| 1932 | 0.752 | 0.678 | 0.834 |
| 1937 | 0.767 | 0.708 | 0.832 |
| 1942 | 0.805 | 0.756 | 0.857 |
| 1947 | 0.828 | 0.789 | 0.869 |
| 1952 | 0.915 | 0.882 | 0.95 |
| 1957 | 1 | 1 | 1 |
| 1962 | 1.036 | 1.005 | 1.069 |
| 1967 | 0.987 | 0.956 | 1.018 |
| 1972 | 0.989 | 0.957 | 1.022 |
| 1977 | 0.989 | 0.953 | 1.026 |
| 1982 | 0.933 | 0.894 | 0.974 |
| 1987 | 0.787 | 0.744 | 0.833 |
| 1992 | 0.723 | 0.66 | 0.792 |
| 1997 | 0.828 | 0.682 | 1.004 |
| 2002 | 0.861 | 0.523 | 1.417 |
| 2007 | 0.809 | 0 | 38568.273 |
| 2012 | 0.844 | 0 | 473125.013 |
| 2017 | 0.895 | 0 | 106257001.784 |
| **local drifts with net drift** | **Percent per year** |  |  |
| 0-4 | 0.608 | -46.176 | 88.056 |
| 5-9 | 0.414 | -35.987 | 57.516 |
| 10-14 | -0.177 | -26.638 | 35.828 |
| 15-19 | -0.648 | -2.121 | 0.847 |
| 20-24 | -1.135 | -1.712 | -0.555 |
| 25-29 | -1.303 | -1.582 | -1.023 |
| 30-34 | -0.877 | -1.055 | -0.698 |
| 35-39 | -0.276 | -0.42 | -0.131 |
| 40-44 | 0.173 | 0.03 | 0.317 |
| 45-49 | 0.658 | 0.488 | 0.829 |
| 50-54 | 1.021 | 0.802 | 1.24 |
| 55-59 | 1.295 | 1.014 | 1.578 |
| 60-64 | 1.139 | 0.777 | 1.501 |
| 65-69 | 0.911 | 0.445 | 1.379 |
| 70-74 | 0.818 | 0.185 | 1.454 |
| 75-79 | 0.956 | 0.068 | 1.853 |
| 80-84 | 1.877 | 0.471 | 3.303 |
| 85-89 | 3.14 | 0.504 | 5.845 |
| 90-94 | 4.155 | -1.582 | 10.227 |
| 95-100 | 4.8 | -10.466 | 22.668 |
| **Coefficients** |  | | |
| **Intercept** | -13.773 | | |
| **LAT** | 0.083 | | |
| **NetDrift** | 0.006 | | |
| **CAT** | 0.077 | | |
